# Structural Activity Relationship Analysis of New Diphenyl PFI-3 Analogues Targeting for the Treatment of Glioblastoma

**DOI:** 10.3390/ph18050608

**Published:** 2025-04-23

**Authors:** Dong-Jin Hwang, Chuanhe Yang, Yinan Wang, Hannah Kelso, Satyanarayana Pochampally, Lawrence M. Pfeffer, Duane D. Miller

**Affiliations:** 1 Department of Pharmaceutical Sciences, College of Pharmacy, University of Tennessee Health Science Center, Memphis, TN 38163, USA; dhwang@uthsc.edu (D.-J.H.); spochamp@uthsc.edu (S.P.); 2 Department of Pathology and Laboratory Medicine, College of Medicine, University of Tennessee Health Science Center, Memphis, TN 38103, USA; cyang@uthsc.edu (C.Y.); ywang127@uthsc.edu (Y.W.); hanrkels@uthsc.edu (H.K.); lpfeffer@uthsc.edu (L.M.P.); 3 The Center for Cancer Research, College of Medicine, University of Tennessee Health Science Center, Memphis, TN 38103, USA

**Keywords:** bromodomain, glioblastoma (GBM), therapeutic enhancing drug (TED), anti-GBM activity, structure–activity relationship (SAR), PFI-3 analog, temozolomide (TMZ)

## Abstract

**Background/Objectives**: Human glioblastoma (GBM) is the most aggressive brain cancer in adults and a highly treatment-refractory malignancy. The overall prognosis for the GBM is extremely poor, with a median survival of 12–14 months after initial diagnosis. Many GBM patients initially respond to the DNA alkylating agent temozolomide (TMZ), but patients often become therapy-resistant, and tumors recur. We previously reported that treatment with PFI-3, which is a small molecule inhibitor of the bromodomain of the BRG1 subunit of the SW1/SNF chromatin remodeling complex, enhanced the sensitivity of GBM cells to TMZ in vitro and in vivo GBM animal models. Our general objective was to perform an SAR study of new diphenyl PFI-3 analogs. **Methods**: New structural analogs of PFI-3 were developed, synthesized, and tested for their ability to enhance TMZ-induced GBM cell death by ELISA. **Results**: Following on the enhanced activity of compounds **2a** and **2b**, new diphenyl PFI-3 analogs with specific structural adjustments were made to better understand the structural requirements to optimize function. Additionally, several new structurally different candidates (e.g., **4a**, **4b**, and **5**) showed much better efficacy in sensitizing GBM cells to TMZ-induced GBM cell death. **Conclusions**: Four series of PFI-3 analogs (**2**, **3**, **4**, and **5**) were designed, synthesized, and tested for the ability to sensitize GBM cells to TMZ-induced cell death. Series 2 optimized the A-ring and R-isomer chirality. Series 3 used a 5-membered linker with weak activity. Series 4’s di-phenyl urea compounds showed better bromodomain inhibition. Series 5’s methoxyphenyl-B-ring analogs were exceptionally strong inhibitors.

## 1. Introduction

**Figure 1:**
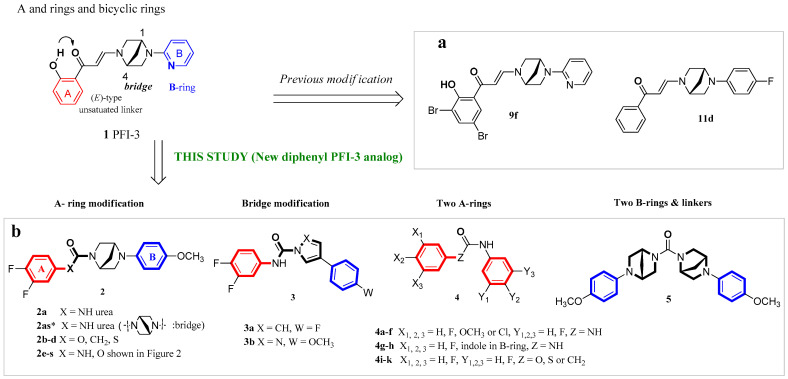
Design and optimization of new diphenyl PFI-3 derivatives and representatives of the modified analogs. *: Compound **2as** (*S*, *S*-type) is the isomer of **2a** (*R*, *R*) of the bicyclic bridge part.

Glioblastoma (GBM) is the deadliest form of brain cancer, and GBM patient survival has not improved for decades. The standard approach for treating GBM patients includes surgical resection, followed by ionizing radiation and adjuvant chemotherapy with the DNA alkylating agent temozolomide (TMZ) [1,2]. However, the use of TMZ alone has limited activity against GBM due to the development of therapeutic resistance [3,4]. We previously found that PFI-3 ((*E*)-1-(2-hydroxyphenyl)-3-((1*R*,4*R*)-5-(pyridin-2-yl)-2,5-diazabicyclo[2.2.1]heptan-2-yl)prop-2-en-1-one) enhances the action of TMZ [5]. PFI-3 (**1** in Figure 1) is a small molecule inhibitor that was developed to target the bromodomains in the BRG1 and BRM catalytic subunits of the SWI/SNF chromatin remodeling complex [6]. The SWI/SNF complex is an evolutionarily conserved multi-subunit complex that is critical for gene regulation, differentiation, DNA repair, and development [7]. Both BRG1 and BRM contain a bromodomain at their carboxy-terminus. Bromodomains are ~110 amino acid domains found in many proteins that recognize acetylated lysine residues, such as those on the *N*-terminal tails of histones, and are responsible for transducing the signal carried by acetylated lysine residues to regulate the cell phenotype [8,9].

**Figure 2:**
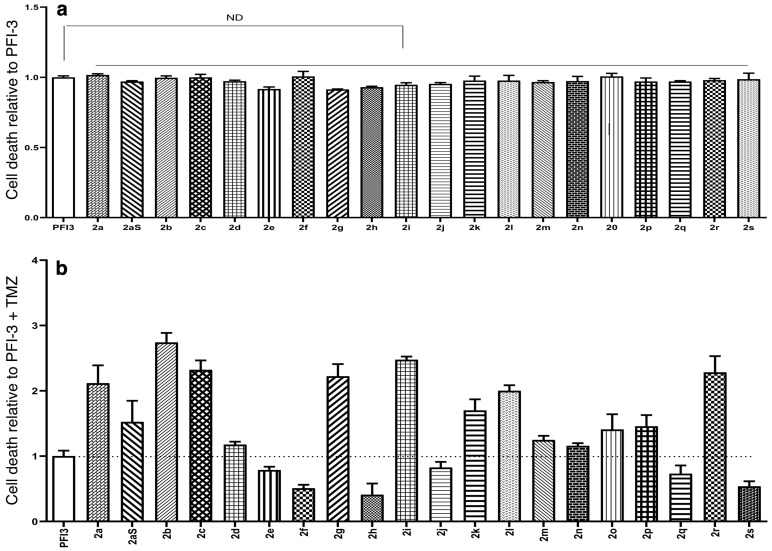
The activity of series **2** with PFI-3 to sensitize GBM cells to TMZ-induced cell death. (**a**) With TMZ. (**b**) Without TMZ. LN229 GBM cells were treated with PFI-3 or the indicated series **2** (20 μM) in combination with TMZ (200 μM). Control cells were treated with vehicle (DMSO). Treatment with TEDs alone had no effect on cell viability. Cell death was determined by ELISA at 72 h, which quantifies histone-complexed DNA fragments. Results were normalized to the GBM cell death-inducing activity of PFI-3 alone (a**A**), or PFI-3 in combination with TMZ (**b**).

**Figure 3:**
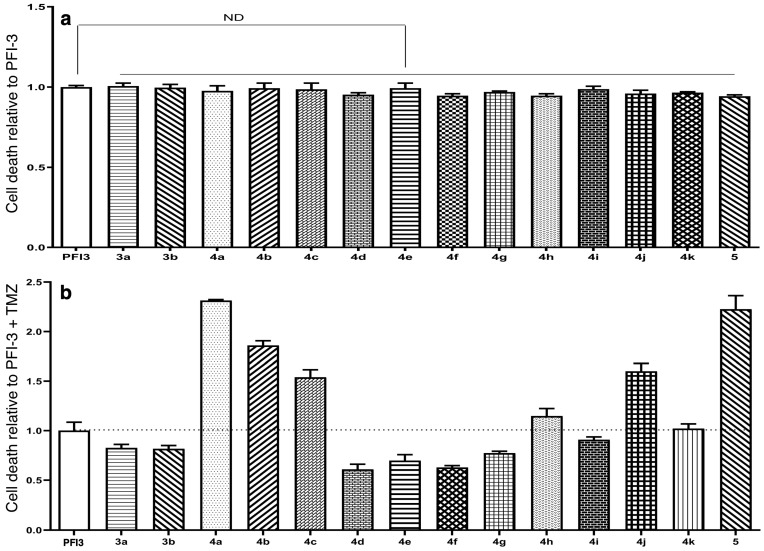
The activity of series **3**, **4**, and **5** to sensitize GBM cells to TMZ-induced cell death. (**a**) Without TMZ. (**b**) With TMZ. LN229 GBM cells were treated with PFI-3 (20 μM) or the indicated series **3**, **4**, or **5** (20 μM) in combination with TMZ (200 μM). Control cells were treated with vehicle (DMSO). Treatment with TEDs alone had no effect on cell viability. Cell death was determined by ELISA at 72 h, which quantifies histone-complexed DNA fragments. Results were normalized to the cell death-inducing activity of the PFI-3 analogs alone (**a**) or in the presence of TMZ (**b**).

We initially developed PFI-3 analogs (denoted as therapeutic enhancing drugs, TEDs) by modifying the PFI-3 structure and we markedly enhanced the TMZ-induced GBM cell death [10]. As described herein, we have made considerable progress in designing and preparing new TEDs that has led to a better understanding of the importance of chemical and structural changes in their ability to enhance TMZ’s anti-GBM activity. Modification of the parent molecule PFI-3 resulted in potent analogs, like **9f** and **11d** [11] (Figure 1a) and **2a** and **2b** [5] (Figure 1b), that were more effective than PFI-3 in enhancing TMZ-induced cell death in GBM cells. Our goal was to better understand the importance of the A- and B-rings, and linker portion of PFI-3 on the ability of these compounds to enhance the anti-GBM activity of TMZ (Figure 1b). We also investigated the chiral bicyclic ring compound **2a** (R-type) and compared it with **2as** (S-type) as shown in Figure 1 on TMZ-induced DNA damage in GBM cells. We then made methylene-amide **2c**, carbamate **2b**, and carbamothioate **2d** and compared their activity to urea **2a** on TMZ-induced death in GBM cells. We then examined the importance of different chemical substitutions on both the A- and B-rings by making compounds **2e**–**2s** and assessed their activity in enhancing TMZ-induced cell death as compared to PFI-3 (**1**, shown in Figure 2). As skeleton diversification on the bridged portion of PFI-3, five-membered rings were investigated in TMZ-induced GBM cell death. The 5-membered bridged compounds **3a** and **3b** had similar activities to PFI-3. To further investigate the SAR of PFI-3 (**1**), two A-ring moieties without a linker and B-ring (**4a**–**k**, in Figure 1b) and two B-ring compounds without an A-ring and linker (**5**, in Figure 1b) were designed and synthesized, which showed extremely potent activity as compared to PFI-3 (Figure 3). Overall, the goal of this study was to find more potent agents and provide new insights into the anti-glioma activity of these newly developed therapeutic-enhancing drugs (TEDs).

## 2. Results and Discussion

### 2.1. Chemistry

We have designed and synthesized new urea, carbamate methylene-amide, and carbamothioate-type PFI-3 derivatives series **2** (including reported compounds **2a**, **2b** [12]), **3**, **4**, and **5**. Based on our previous findings with the first generation of TEDs [5], we now performed additional SAR, which led to a generation of new analogs as shown in Figure 1. We describe herein the design and synthetic methods to understand the structure needed for targeting the bromodomains of brain cells. Structural modifications were made in the A-ring and B-rings and in the linker, which included part A in **2**, **3**, and **4** in Figure 1b and B-rings, the linker, and bridged bicyclic fragments.

**Scheme 1:**
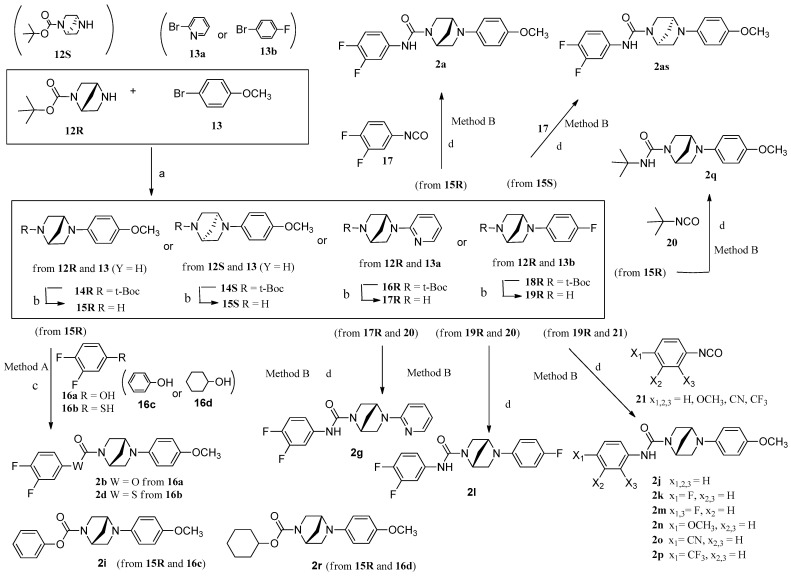
General synthetic route for the preparation of target compounds **2a**, 2**b**, and **2h**–**q**. Reagents and conditions: (a) BINAP, Pd(OAc)_2_, *t*-BuONa, toluene, reflux; (b) i. EtOH, AcCl, 0 °C-rt, ii. Et_3_N, DCM, rt; (c) bis(trichloromethyl) carbonate (BTC, Triphosgene), (3,4-difluoroaniline (for **2a**), 3,4-difluorophenol (for **2b**), or 3,4-difluorobenzenethiol (for **2s**), Et_3_N, DCM, 0 °C-rt; (d) DCM, Et_3_N, 0 °C-rt, final yield between 50% and 88%.

As shown in Figure 1a, we made analogs of the PFI-3 bromodomain inhibitor by modification of the substituted phenol A-ring part and pyrido-B-ring, which resulted in improved GBM cell death-inducing activity of TMZ in compounds **9f** and **11d** when compared to PFI-3 [6]. Based on the findings gained from the structural biology study, we conducted re-scaffolding studies and discovered a new generation of bromodomain inhibitors. In this study, we developed new TEDs, whose modifications are described below:

- Modification of the A-ring part of PFI-3 to convert to a substituted benzene ring.
- Modification of the linker to give urea (for **2a**), carbamate (for **2b**), methylene-amide (for **2c**), and carbamothioate (for **2d**) instead of an unsaturated linker in PFI-3.
- Modification of the bicyclic (1*R*, 4*R*)-type isomer (for **2a**) bridge with a (1*S*, 4*S*) isomer (for **2as**) shown at the bottom of Scheme 1.
- Multi- (or mono-) substitution of the A- or B-rings shown in Figure 1b.
- Skeleton diversification; we focused on a 5-membered ring on the bridge part of PFI-3 for compounds **3a** and **3b** and two A-rings (for **4a**–**k**) and two B-ring derivatives (for **5**).

Urea (i.e., **2a**, **2e**–**h**, etc.), carbamate (i.e., **2b**, **2i**, **2r**), and carbamothioate **2d** were synthesized as shown in Scheme 1. Two synthetic methods, A and B, from free amine **15R** with corresponding aromatic isocyanate **17** or aromatic aniline **16** via bis(trichloromethyl) carbonate (BTC) mediated with 3,4-difluoroaniline were utilized to prepare urea **2a**, as shown in Scheme 1. **2as** (isomer of **2a**) was prepared with the initialization of *S*-isomer **12S** through chiral auxiliary by the same method as the **2a** procedure. The combination of substituted phenol **16a** and **15R** was employed to produce carbamate (**2b**, **2i**, and **2r**). Carbamothioate **2d** is produced from **15R** and commercial 3,4-difluorobenzenethiol via BTC activation in the same way.

**Scheme 2:**
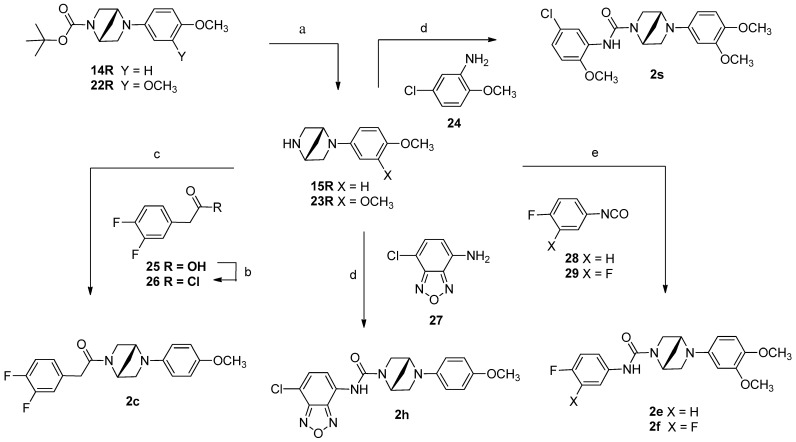
General synthetic route for the preparation of target compounds **2c**, **2e**, 2**f**, and **2s**. Reagents and conditions: (a) i. EtOH, AcCl, 0 °C-rt, ii. Et_3_N, DCM, rt; (b) SOCl_2_, THF, 0 °C to rt; (c) Et_3_N, DCM, rt, 0 °C-rt; (d) bis(trichloromethyl) carbonate (BTC or Triphosgene), Et_3_N (or pyridine), DCM, rt; (e) DCM, Et_3_N (or pyridine), rt final yield between 53 and 72%.

The essential intermediate **15R** was synthesized from protected chiral **14R** by the acid-condition deprotected synthetic method. The protected **14R** was prepared by the Buchwald–Hartwig reaction using Pd(OAc)_2_ and BINAP to catalyze the cross-coupling of protected amine **12R** with aryl bromide **13** [13]. The preparation of aliphatic A-ring compounds (**2q** and **2r**) was conducted by using 2-isocyanato-2-methylpropane (for **2q**) and cyclohexanol (for **2r**) instead of **17** or **16** as reactants each through the same procedure as shown in Scheme 1. Methylene amide **2c** and several ureas (i.e., **2e**, **2f**, **2h**, and **2s**) were summarized in Scheme 2. Protected amines **14R** or **22R**, prepared by the Buchwald–Hartwig reaction, were used as starting materials to produce designed compounds (**2c**, **2e**, **2f**, and **2s**), as shown in Scheme 2. The protected amine **14R** or **22R** produced free amine **25R** or **26R** by deprotection mediated acidic solution. The reaction of compound **15R** with activated acid chloride **26** generated target methylene amide **2c** under basic conditions. For compounds **2h** and **2s**, bis(trichloromethyl) carbonate-mediated reaction of free amine **14R** and corresponding anilines **24** and **27** produced the target compounds **2h** and **2s** under basic conditions using triethylamine or pyridine at room temperature. The synthesis of **2e** and **2f** was achieved through isocyanate 28 or 29 reacting with corresponding aniline 15R or 23R at room temperature.

**Synthesis of compound 2**

**Scheme 3:**
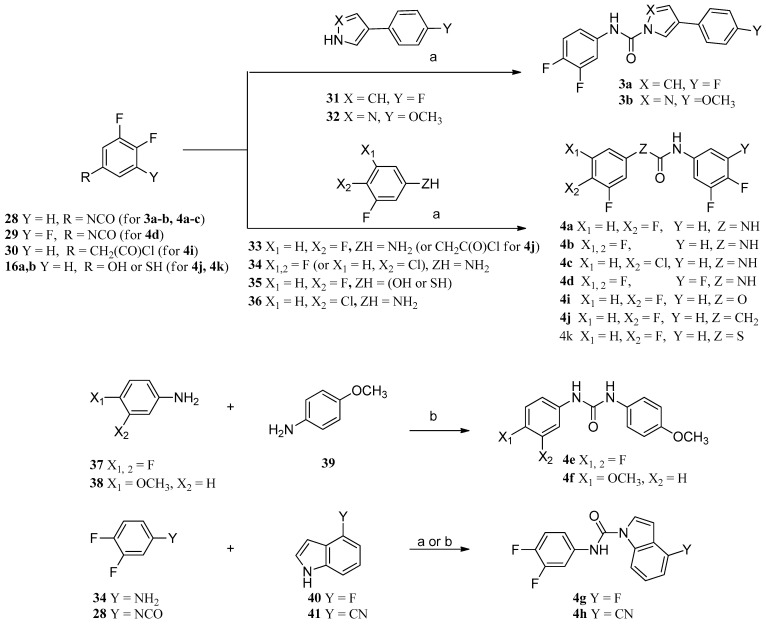
General synthetic route for the preparation of target compounds **3a**,**b** and **4a**–**k**. Reagents and conditions: (a) DCM, Et_3_N, rt.; (b) bis(trichloromethyl) carbonate (BTC or Triphosgene), Et_3_N, DCM, rt final yield between 67 and 89%.

**Scheme 4:**
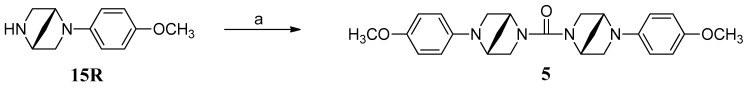
General synthetic route for the preparation of target compound **5**. Reagents and conditions: (a) bis(trichloromethyl) carbonate, Et_3_N, DCM, rt, yield 73%.

Finally, the preparation of modified linker derivates **3a**,**b** and bis-aromatic analogs **4a**–**h** is shown in Scheme 3. The synthetic approach of **3a**,**b** and **4a**–**d** was performed with the isocyanates **28** and **29** with corresponding anilines **31**–**36** in basic conditions under an anhydrous atmosphere. And in using bis(trichloromethyl) carbonate (BTC), two different anilines (**34** and/or **37**–**40**) generated **4e**–**g** as asymmetric urea-type compounds. The reaction of indole **41** with isocyanate **28** produced urea-type compound **4h**. The compounds **4i**, **4j**, and **4k** were generated by reaction of substituted phenol (or thiol) **30** or acid chloride **35** (for **4j**) with corresponding carboxylic acid by treating SOCl_2_ as shown in Scheme 3. In Scheme 4, the mediated reaction of bis(trichloromethyl) carbonate (BTC) with **37R** produced **5** as a symmetric product with **5a** as a byproduct.

### 2.2. Structure–Activity Relationship (SAR) Exploration of Series ***2***, ***3***, ***4***, and ***5***

**Figure 4:**
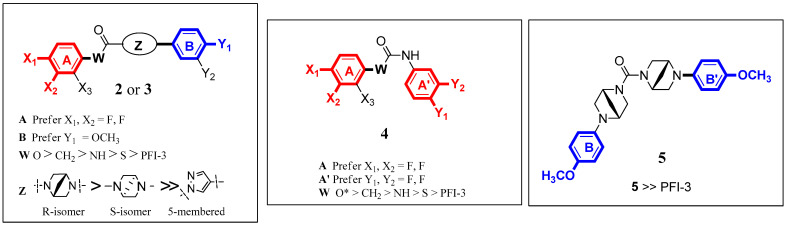
Summary of SAR study and for the ability of the optimized structures of the series **2**, **3**, **4**, and **5** to sensitize GBM cells to TMZ-induced cell death. *: Comparison of R-type bromodomain inhibitor activity to the S-type linker.

We explored the structural activity relationship (SAR) of scaffolded analogs **2**, **3**, **4**, and **5**, as demonstrated in Figure 2 and Figure 3. Like PFI-3, none of the newly designed analogs had any effect on GBM cell death (Figure 2a and Figure 3a). However, when treated in combination with TMZ, some of the newly designed analogs of (**2**, **3**, **4**, and **5**) markedly increased GBM sensitivity to the cell-death-inducing activity of TMZ (Figure 2b and Figure 3b). We have previously shown that 200 μM TMZ had only a minimal effect on GBM cell viability [13]. It is important to note that compounds of series **2** (**2a**, **2b**, **2c**, **2d**, **2g**, **2i**, **2k**, **2l**, and **2r**), **4** (**4a**, **4b**, **4c**, **4g**, and **4j**), and **5** were found to be superior to PFI-3 in enhancing the activity of TMZ to induce GBM cell death, as summarized in Figure 4. Showing the importance of chirality in the bromodomain inhibitors, the R-isomer **2a** (Figure 1) had greater activity than the S-isomer **2as** (Scheme 1), showing the highest binding for the R-isomer. Further refinements of the structure by inserting a 5-membered ring in compounds **3a** and **3b** (Scheme 3) showed their activity in GBM cells to be less in the TMZ-induced cell relative to PFI-3. Treatment of GBM cells with dimeric compound **5** resulted in an increase in the activity of TMZ-induced cell death of two times compared to PFI-3 (**1**). Moreover, removal of the bridge portion in compounds (urea **4a** and carbamate **4b**) resulted in compounds with high sensitivity to death-inducing activity with TMZ.

In summary, the structure–activity relationship studies of TED series **2**, **3**, **4**, and **5** specified that modification (i.e., A- and B-ring, bicyclic bridge, A-ring only, and B-ring only) suggests that there are certain indispensable structural motifs that must be retained to enhance TMZ-induced cell death. On the structural modifications, the structural requirement in these new TEDs to sensitize GBM cells to TMZ-induced death as new bromodomain inhibitor scaffolds are summarized as shown in Figure 4.

The SAR study and considerations for the series of analogs of **2**, **3**, **4**, and **5** to sensitize GBM cells to TMZ-induced cell death are summarized as below:

- Optimizing A-ring (Series **2**, Y_1_ = OCH_3_):
  3,4-di-fluorophenyl (for **2a**) > unsubstituted phenyl (**2i**) > cyclohexyl (**2r**) >> PFI-3 (**1**)
- Linker modification (Series **2**):
  carbamate **2b** (W = O) > methylene-amide **2c** (W = CH_2_) > urea **2a** (W = NH) > carbamothioate **2d** (W = S) > PFI-3 (**1**).
- Bridge Z:
  bicyclic R-isomer **2a** > bicyclic S-isomer **2as** > PFI-3 **1** > 5-member ring **3a**, **3b**.
- Two A-rings (Series **4**):
  2-fluoro substituents in each A- and B-ring (X_1_, X_2_, Y_1_, and Y_2_ = F, for **4a**) > 5-fluoro substituents in both A- and B-rings (X_1_, X_2_, X_3_, Y_1_, and Y_2_ = F, for **4b**) > 3-fluoro substituents in both A- and B-rings (X_1_, X_2_, and Y_1_ = F, Y_2_ = Cl, for **4c**) > 4-cyanoindole in the B-ring (for **4h**), and PFI-3 (**1**) > mono-fluoro or methoxy substituents (for **4e**, **4f**).
- Interestingly, dimeric bicyclic compound **5** (i.e., two B-rings) presented very strong activity (2.5 times stronger than PFI-3 as shown in Figure 4. It seems to be influenced by “a symmetric bicyclic B-ring” that fits well to the target bromodomain binding site.

### 2.3. Calculated Properties of PFI-3 and Compounds in the Series ***2***, ***3***, ***4***, and ***5***

We next formed in silico analysis for the drug properties of compounds in series **2**, **3**, **4**, and **5**, which included high gastrointestinal (GI) absorption, penetration in the blood–brain barrier (BBB), and optimized physical properties in computer simulation, as shown in Table 1 and Supplementary Materials. The constituents of the new modified PFI-3 analog series **2**, **3**, **4**, and **5** were examined by the Swiss ADME computer-aided prediction model [14] to predict drug-like properties. The synthesized series **2**, **3**, **4**, and **5** calculated physical properties, such as ADME (Absorption, Distribution, Metabolism, and Excretion) and other drug-like properties shown in Table 1 and Figure S1 (in the Supplementary Materials). In Figure S1, the round yellow yolk-like sphere represents the BBB (blood–brain barrier) permeation region, and the oval white part represents the HIA (Human Intestinal Absorption) region. The gray region represents the low absorption and limited brain permeation region. The right-hand side box provides the option to show the molecule modified PFI-3 analogs **2**–**5**, legends of the BOILED-Egg model, and other remarks. The query of modified PFI-3 analogs **2**–**5** is visualized as a red/blue hollow sphere located in the white part (HIA). The red/blue color represents PGP+/− (P-glycoprotein positive/negative) based on its property located in the white region, resulting also in penetrable properties in BBB and optimized performance in physical properties. The designed series **2**–**5** are the Bioavailability Radar calculated in several physical properties as marked as LIPO (Lipophilicity), SIZE, POLAR (Polarity), INSOLU (Insolubility), INSATU (Instauration; 0.25 < Fraction Csp3 < 1), and FLEX as shown in Figure S1. Table 1 showed the key factors of physical properties for drug-likeness, such as physicochemical, pharmaceutical, and drug-likeness properties of modified PFI-3 analogs selected **2**–**5**, especially high GI absorption and penetrable properties on BBB with high bioavailability matched with Lipinski’s Rules of Five [15].

### 2.4. Drug-likeness: Computer-Aided Predictability of Selected ***2a***, ***4a***, and ***5***

Compared to the drug properties of compounds **2a** and **2b** (new-PFI-3), **4a** (two A-rings), and **5** (two B-rings), we evaluated the selected molecules, **2a**, **2b**, **4a**, and **5**, through computational methods in the SwissADME [14] program.

**Figure 5:**
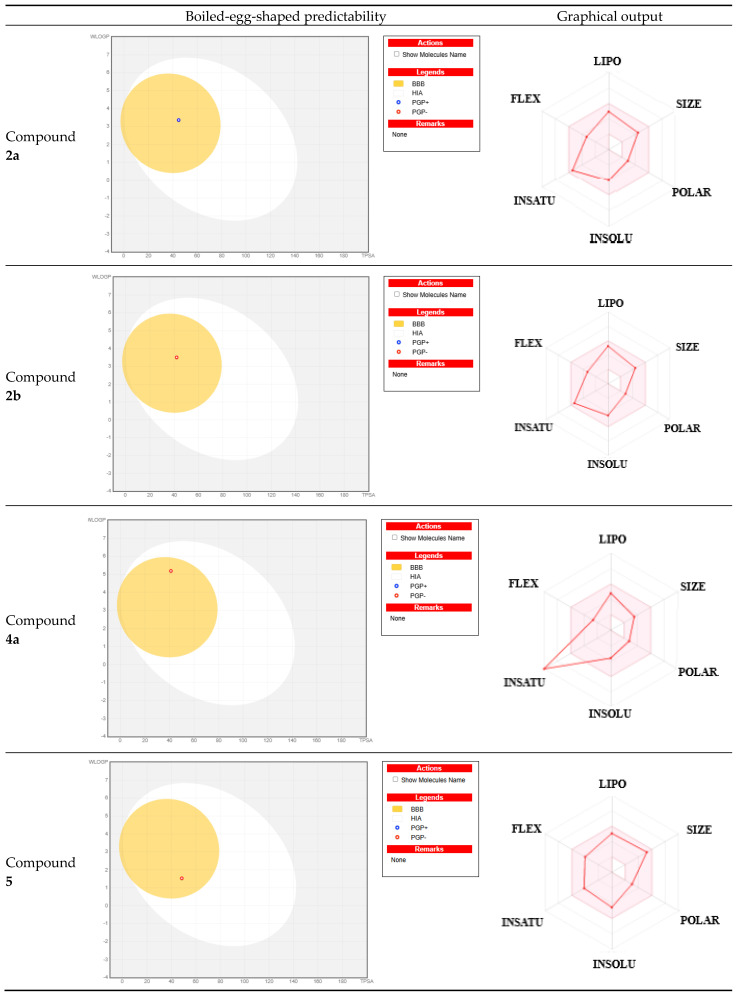
BOILED-Egg model of representative compounds **2a**, **2b**, **4a**, and **5** and graphical output of the calculated ADME studies by SwissADME (for **2a**, **2b**, **4a**, and **5**).

The compounds **2a**, **2b**, **4a**, and **5** calculated and expected physical properties, called ADME (Absorption, Distribution, Metabolism, and Excretion) and other drug likenesses to use the pharmaceutical aids as shown in Table 1 and Figure 5. As calculated, compound **2a** was predicted to be effluxed from the central nervous system by the p-glycoprotein (PGP+; blue spot). However, compounds **2b**, **4a**, and **5** were not predicted to be effluxed by the p-glycoprotein (PGP−; red spot), as shown in Figure 5. Consequently, the results of physicochemical, pharmacokinetic, and drug-likeness properties of especially compounds **2a**, **2b**, **4a**, and **5** showed great drug properties as GBM treatment, as shown in Table 1 and Figure 5.

## 3. Materials and Methods

### 3.1. Biological Reagents and Cell Cultures

U87 and LN229 (ATCC, Manassas, VA, USA) GBM cell lines were grown in DMEM containing 10% fetal bovine serum (Hyclone, Logan, UT, USA) supplemented with penicillin (100 IU/mL) and streptomycin (100 μg/mL) at 37 °C with 5% CO_2_. The cells were authenticated by short-tandem repeat analysis.

### 3.2. Cell Death Assays

For cell death assays, cells were plated into 48-well plates (1 × 10^4^ cells/well), and after 2 days of drug treatment, the levels of apoptosis in the attached cells were determined according to the instructions using the cell death ELISA^PLUS^ assay (Roche, Basel, Switzerland), which measures cytoplasmic histone-associated DNA fragments [16].

### 3.3. General Chemistry Methods

All chemicals for synthesis were purchased from Sigma-Aldrich Chemical Co., Fisher Scientific (Pittsburgh, PA, USA), Ambeed, Inc. (Arlington Heights, IL, USA), Combi-Blocks, Inc. (San Diego, CA, USA), 1Pluschem Product List (San Diego, CA, USA), etc., and used without further purification. Moisture-sensitive reactions were carried out under an argon atmosphere. Analytical thin-layer chromatography (TLC) was performed on pre-coated silica gel (Merck Kieselgel 60 F254 layer thickness 0.25 mm, Rahway, NJ, USA). A Bruker Avance III 400 (Billerica, MA, USA) spectrometer obtained NMR spectra. Chemical shifts are observed as parts per million (ppm) relative to TMS in CDCl_3_ or DMSO-d_6_. The structure of synthesized compounds was also utilized by 1H-1H 2D-COSY and 2D-NOE NMR analytic methods. The use of silica gel (230–400 mesh, Merck) for flash column chromatography was utilized. A Bruker Esquire-LC/MS system (Bruker Daltonics, Billerica, MA, USA) equipped with an electrospray/ion trap instrument in positive and negative ion modes (ESI source). The purity of the final compounds was examined by an Agilent 1100 HPLC system (Santa Clara, CA, USA). HPLC conditions: 45% acetonitrile at a flow rate of 1.0 mL/min using a LUNA 5 μ C18 100A column (250 × 4.60 mm) purchased from Phenomenex (Torrance, CA, USA) at ambient temperature. The UV detection was set at 340 nm or 245 nm. The properties of the compounds were established by careful integration of areas for all peaks detected and determined as more than 95% for all compounds tested for biological study.

### 3.4. Synthesis of Compound Series ***2***–***5***

Method A.

A 100 mL, oven-dried, two-necked, round-bottomed flask is charged with a Teflon-coated magnetic oval stir bar and coupled with a 50 mL dropping funnel. Both the dropping funnel and the round-bottomed flask are sealed with a rubber septum. Under an argon atmosphere, to a solution of triphosgene (520 mg, 1.75 mmol) in 10 mL of dry THF, **15S** (325 mg, 1.6 mmol) in 3 mL of THF was added slowly at 0 °C. The resulting mixture was stirred at the same temperature for 10 min and allowed to stir at room temperature for another 30 min. After completion of the reaction, the solution of substituted aniline (1.48 mmol) with 1 mL of Et_3_N was added to the mixture and stirred overnight at room temperature. The solution was concentrated under reduced pressure and poured into EtOAc, then washed with saturated NaHCO_3_ solution and water, dried over anhydrous MgSO_4_, and concentrated under reduced pressure to purify by silica gel chromatography (EtOAc/n-hexane = 1:1) or (hexane/acetone = 3:1, *v*/*v*) to afford the designed compound.

Method B.

Under a nitrogen atmosphere, to a solution of isocyanate (**23**, **24**, or **25**, 2.2 mmol) in 10 mL of dry DCM, aniline (**15R**, **18R**, **26**, or **27**, 2 mmol) in 3 mL of DCM and triethylamine (0.3 mL) were added slowly at 0 °C. The resulting mixture was stirred at the same temperature for 10 min and allowed to stir at room temperature for another 30 min. After completion of the reaction, the reaction mixture was added to crushed ice and extracted with DCM. The organic layer was dried over anhydrous MgSO_4_, and concentrated under reduced pressure to purify by silica gel chromatography (EtOAc/n-hexane = 1:2) or (acetone/hexane = 1:3, *v*:*v*) to afford the target compound.

Method C.

To a solution of (1*R*,4*R*)-*tert*-butyl 2,5-diazabicyclo[2.2.1]heptane-2carboxylate (**12R**, 5 mmol) in anhydrous toluene (30 mL) was added substituted bromobenzene (**13**, 10 mmol), sodium *tert*-butoxide (5 mol), Pd(OAc)_2_ (0.25 mmol), and (*R*)(+)-2,2′-bis(diphenylphosphino)-1,1′-binaphthalene (BINAP, 0.25 mmol) at room temperature under the argon atmosphere. The resulting reaction mixture was heated at reflux for 4–5 h under an argon atmosphere. After the end of the reaction was monitored by TLC, the reaction was quenched by water and extracted with ethyl acetate. The organic layer was dried with anhydrous MgSO_4_, filtered, concentrated under reduced pressure, and purified by column chromatography using hexanes and ethyl acetate (1:1, *v*/*v*) as eluent to afford the desired compound.

Method D.

The solution of **14R** (40.36 mmol) was dissolved in anhydrous ethanol (30 mL) in a 100 mL round-bottomed flask. To this, 5 mL of acetyl chloride was dropwise added in an ice-water bath and was stirred overnight at room temperature under argon conditions. The reaction was monitored by TLC using an ethyl acetate and hexane (2:3, *v*/*v*) system. Stirring was continued until TLC indicated the completion of the reaction. The solution was reduced off under reduced pressure. The solvent was removed completely under vacuum to obtain compound **15R**.

Synthesis of compounds **3a** and **3b**.

A 100 mL, oven-dried, two-necked, round-bottomed flask is charged with a Teflon-coated magnetic oval stir bar and coupled with a 50 mL dropping funnel. Both the dropping funnel and the round-bottomed flask are sealed with a rubber septum. Under a nitrogen atmosphere, to a solution of 1,2-difluoro-4-isocyanatobenzene (**28**, 320 mg, 2.06 mmol) in 10 mL of dry DCM, 3-(4-fluorophenyl)-1*H*-pyrrole (**31**, for **3a**) or 4-(4-methoxyphenyl)-1*H*-pyrazole (**32**, for **3b**) (1.6 mmol) in 3 mL of DCM and triethylamine (0.2 mL) was added slowly at 0 °C. The resulting mixture was stirred at the same temperature for 10 min and allowed to stir at room temperature for another 30 min. After completion of the reaction, the reaction mixture was added to crushed ice and extracted with DCM. The organic layer was dried over anhydrous MgSO_4_ and concentrated under reduced pressure to purify by silica gel chromatography (acetone/hexane = 1:4, *v*/*v*) to afford the target compounds.

**(1*R*,4*R*)-*tert*-Butyl 5-(pyridin-2-yl)-2,5-diazabicyclo[2.2.1]heptane-2-carboxylate (16R)**

Prepared by Method C.

MS (ESI) *m*/*z* 276.20 [M + H]^+^; LCMS (ESI) *m*/*z* calcd for C_15_H_22_N_3_O_2_: 276.1712 [M + H]^+^, found: 276.1716 [M + H]^+^. ^1^H NMR (400 MHz, DMSO-d_6_) δ 8.06 (d, *J* = 4.8 Hz, 1H, ArH), 7.51–7.47 (m, 1H, ArH), 6.60–6.57 (m, 1H, ArH), 6.54–6.51 (m, 1H, ArH), 4.77 (d, *J* = 8.8 Hz, 1H, CH), 3.51–3.45 (m, 1H, CH), 3.44–3.30 (m, 2H, CH), 3.26–3.33 (m, 1H, CH), 3.18–3.15 (m, 1H, CH), 1.90–1.87 (m, 2H, CH), 1.39 (s, 6H, 2xCH_3_), 1.34 (s, 3H, CH_3_).

**(1*R*,4*R*)-2-(pyridin-2-yl)-2,5-diazabicyclo[2.2.1]heptane (17R)**

Prepared by Method D.

MS (ESI) *m*/*z* 176.06 [M + H]^+^, 198.09 [M + Na]^+^. ^1^H NMR (400 MHz, DMSO-d_6_) δ 8.13 (d, *J* = 4.8 Hz, 1H, ArH), 7.48–7.43 (m, 1H, ArH), 6.63–6.58 (m, 1H, ArH), 6.37–6.32 (m, 1H, ArH), 5.03(d, *J* = 7.8 Hz, 1H, CH), 4.54–4.50 (m, 1H, CH), 3.68–3.65 (m, 1H, CH), 3.61–3.49 (m, 2H, CH), 3.44–3.38 (m, 1H, CH), 2.12–1.91(m, 2H, CH).

**(1*R*,4*R*)-*tert*-Butyl 5-(4-methoxyphenyl)-2,5-diazabicyclo[2.2.1]heptane-2-carboxylate (14R)**

Prepared by Method C.

White solid. Yield (75%). UV max: 249.45, 315.45 nm. Purity (LC: *t_R_* 3.60 min): 97.54%. MS (ESI) *m*/*z* 305.15 [M + H]^+^. LCMS (ESI) *m*/*z* calcd for C_17_H_24_N_2_O_3_: 305.1865 [M + H]^+^, found: 305.1853 [M + H]^+^. ^1^H NMR (400 MHz, CDCl_3_) δ 6.84 (t, *J* = 8.4 Hz, 2H, ArH), 6.52 (dd, *J* = 8.4, 6.0 Hz, 2H, ArH), 4.50 (d, *J* = 58.4 Hz, 1H), 4.31 (s, 1H), 3.76 (s, 3H, OCH3), 3.58 (m, 1H), 3.49 (dd, *J* = 31.6, 10.0 Hz, 1H), 3.35 (t, *J* = 12.8 Hz, 1H), 3.14 (dd, *J* = 38.0, 8.4 Hz, 1H), 1.98 (m, 1H), 1.90 (m, 1H), 1.44 (s, 3H), 1.40 (s, 6H).

**(1*R*,4*R*)-*tert*-Butyl 5-(3,4-dimethoxyphenyl)-2,5-diazabicyclo[2.2.1]heptane-2-carboxylate (** **22R)**

Prepared by Method C.

White-off solid. Yield 70%. MS (ESI) *m*/*z* 335.12 [M + H]^+^; LCMS (ESI) *m*/*z* calcd for C_18_H_26_N_2_O_4_: 335.1971 [M + H]^+^, found: 335.1989 [M + H]^+^. ^1^H NMR (400 MHz, DMSO-d_6_) δ 6.78 (d, *J* = 8.4 Hz, 1H, ArH), 6.22 (d, *J* = 2.4 Hz, 1H, ArH), 6.06 (dd, *J* = 8.4, 2.4 Hz, 1H), 4.41 (bs, 1H, NH), 4.39 (m, 1H), 3.73 (s, 3H, OCH_3_), 3.63 (s, 3H, OCH_3_), 3.51 (m, 1H), 3.28 (m, 1H), 3.23 (m, 1H), 2.90 (t, *J* = 9.8 Hz, 1H), 1.88 (m, 1H), 1.87 (m, 1H), 1.40 (s, 6H), 1.32 (s, 3H). ^13^C NMR (100 MHz, DMSO-d_6_) δ 153.92, 150.60, 142.72, 140.80, 114.87, 104.14, 99.18, 79.07, 58.09, 57.54, 56.96, 56.53, 55.91, 51.21, 37.55, 28.58.

**(1*R*,4*R*)-2-(3,4-Dimethoxyphenyl)-2,5-diazabicyclo[2.2.1]heptane (23R)**

Prepared by Method D.

Light brown solid. Yield 95%. LCMS (ESI) *m*/*z* calcd for C_13_H_18_N_2_O_2_: 235.1447 Found: 235.1502 [M + H]^+^. ^1^H NMR (400 MHz, DMSO-d_6_) δ 9.80 (bs, 1H, NH), 9.02 (bs, 1H, NH), 6.82 (d, *J* = 8.8 Hz, 1H, ArH), 6.29 (d, *J* = 2.0 Hz, 1H, ArH), 6.12 (dd, *J* = 8.8, 2.0 Hz, 1H), 3.72 (s, 3H, OCH_3_), 3.78 (m, 1H), 3.64 (s, 3H, OCH_3_), 3.64–3.52 (m, 2H), 3.26 (d, *J* = 10.0 Hz, 1H), 3.17–3.12 (m, 2H), 2.08 (d, *J* = 10.4 Hz), 1.93 (d, *J* = 10.4 Hz, 1H). ^13^C NMR (100 MHz, DMSO-d_6_) δ 150.41, 141.58, 141.52, 114.67, 104.88, 99.81, 57.65, 56.88, 56.06, 52.89, 48.69, 35.95.

**(1*R*,4*R*)-*N*-(3,4-Difluorophenyl)-5-(4-methoxyphenyl)-2,5-diazabicyclo[2.2.1]heptane-2-carboxamide (2a)**

Prepared by Method A and B.

White off solid. Yield 80%. UV max: 195.45, 230.45. Purity (LC, *t_R_* 3.10 min) 97.09%. MS (ESI) *m*/*z* 360.15 [M + H]^+^; 358.23 [M − H]^−^. LCMS (ESI) *m*/*z* calcd for C_19_H_19_F_2_N_3_O_2_: 360.1524 [M + H]^+^; Found: 360.1525 [M + H]^+^; ^1^H NMR (400 MHz, DMSO-d_6_) δ 8.43 (bs, 1H, NH), 7.65–7.59 (m, 1H, ArH), 7.03 (*q*, *J* = 9.60 Hz, ArH), 7.21 (m, 1H, ArH), 6.79 (*d*, *J* = 8.8 Hz, 2H, ArH), 6.56 (*d*, *J* = 8.8 Hz, 2H, ArH), 4.67(s, 1H), 4.49 (s, 1H), 4.49 (s, 1H), 3.64 (s, 3H, OCH_3_), 3.55 (d, *J* = 8.0 Hz, 1H), 3.39 (s, 2H), 2.94 (d, *J* = 8.8 Hz, 1H), 1.98 (d, *J* = 10.8 Hz, 1H), 1.91 (d, *J* = 8.8 Hz, 1H). ^19^F NMR (400 MHz, DMSO-d_6_) δ -136.52, -145.34.

**(1*R*,4*R*)-3,4-Difluorophenyl 5-(4-methoxyphenyl)-2,5-diazabicyclo[2.2.1]heptane-2-carboxylate (2b)**

Prepared by Method A.

Light yellowish solid. Yield 51%. MS (ESI) *m*/*z* 361.12 [M + H]^+^. UV max: 240.45, 315.45 nm. Purity (LC, *t_R_* 3.64 min) 98.77%. LCMS (ESI) *m*/*z* calcd for C_19_H_18_F_2_N_2_O_3_: 361.1364 [M + H]^+^, found: 361.1353 [M + H]^+^. ^1^H NMR (400 MHz, DMSO-d_6_) δ 7.46–7.40 (m, 1H, ArH), 7.38–7.32 (m, 1H, ArH), 7.05–6.95 (m, 1H, ArH), 6.82 (d, *J* = 8.4 Hz, 1H), 6.62 (d, *J* = 8.4 Hz, 1H), 4.69 (s, 1H), 4.55 (d, *J* = 6.0 Hz, 1H), 3.65 (s, 3H, OCH_3_), 3.62 (m, 2H), 3.58–3.32 (m, 3H), 3.16 (d, *J* = 8.8 Hz, 1H), 2.02 (m, 1H), 1.98 (m, 1H). ^19^F NMR (400 MHz, DMSO-d_6_) δ -136.50, -142.66

**2-(3,4-Difluorophenyl)-1-((1*R*,4*R*)-5-(4-methoxyphenyl)-2,5-diazabicyclo[2.2.1]heptan-2-yl)ethenone (2c)**

Under a nitrogen atmosphere, thionyl chloride (18.4 mL, 0.252 mol) was added dropwise to a cooled solution (less than 4 °C) of 2-(3,4-difluorophenyl)acetic acid (206 mg, 1.2 mmol) in 7 mL of THF under an argon atmosphere. The resulting mixture was stirred for 3 h under the same conditions. The solution was added dropwise to a solution of **15R** (204 mg, 1 mmol) in DCM/Pyridine (2 mL/5 mL) and stirred overnight at rt. The solvent was removed under reduced pressure and diluted with EtOAc (20 mL), washed with H_2_O, 5% HCl solution, and brine (300 mL). The organic layer was dried over anhydrous MgSO_4_ and concentrated under reduced pressure to give a crude solid, which was purified by column chromatography using acetone/hexane (1/4, *v*/*v*) to give a solid.

Light yellowish solid. Yield 53%. MS (ESI) *m*/*z* 359.12 [M + H]^+^. UV max: 190.45, 209.45, 249.45 nm. Purity (LC, *t_R_* 3.06 min) 98.73%. LCMS (ESI) *m*/*z* calcd for C_20_H_20_F_2_N_2_O_2_: 359.1571 [M + H]^+^; Found: 359.1578 [M + H]^+^. ^1^H NMR (400 MHz, DMSO-d_6_) δ 7.35 (m, 1H, ArH), 7.24 (m*,* 1H, ArH), 7.15 (m*,* 1H, ArH), 6.78 (m, 2H, ArH), 6.52 (m, 2H, ArH), 4.78 (d, *J* = 25.6 Hz, 1H), 4.51 (d, *J* = 37.6 Hz, 1H), 3.76 (d, *J* = 15.6 Hz, 1H), 3.65 (s, 3H, OCH_3_), 3.55 (m, 1H), 3.51–3.44 (m, 2H), 3.29 (s, 1H), 2.87 (t, *J* = 10.2 Hz, 1H), 2.01 (d, *J* = 9.6 Hz, 1H), 1.93 (d, *J* = 9.6 Hz, 1H). ^19^F NMR (400 MHz, DMSO-d_6_) δ -139.59, -142.26.

**(1*R*,4*R*)-*S*-(3,4-Difluorophenyl) 5-(4-methoxyphenyl)-2,5-diazabicyclo[2.2.1]heptane-2-carbothioate (** **2d)**

Under an argon atmosphere, to a solution of triphosgene (445 mg, 1.5 mmol) in 10 mL of anhydrous THF, 3,4-difluorothiophenol (219 mg, 1.5 mmol) in 3 mL of THF was added slowly at 0 °C to the solution, and triethylamine (0.28 mL, 2 mmol) was added dropwise to the solution. The resulting mixture was stirred at the same temperature for 10 min and allowed to heat to reflux for 30 min. After cooling the flask, the solution of **15R** (306 mg, 1.5 mmol) in 2 mL anhydrous THF was added to the mixture and stirred overnight at room temperature. The reaction mixture was concentrated under reduced pressure and then dissolved into 30 mL of ethyl acetate and washed with water (30 mL) brine (30 mL). The organic layer was dried over anhydrous MgSO_4,_ and concentrated under reduced pressure and purified with flash column chromatography using EtOAc/Hex (1:2, *v*/*v*) to give a white solid. Yield 53%. MS (ESI) *m*/*z* 377.10 [M + H]^+^. UV max: 192.45, 248.45, 249.45 nm. Purity (LC, *t_R_* 3.53 min): 98.39%. LCMS (ESI) *m*/*z* calcd for C_19_H_18_F_2_N_2_O_2_S: 377.1135 [M + H]^+^; Found: 359.1136 [M + H]^+^. ^1^H NMR (400 MHz, DMSO-d_6_) δ 7.60–7.57 (m, 1H, ArH), 7.47–7.43 (m*,* 1H, ArH), 7.29 (m*,* 1H, ArH), 6.78 (m, 2H, ArH), 6.52 (m, 2H, ArH), 4.78 (m, 1H), 4.56 (s, 1H), 3.66 (s, 3H, OCH_3_), 3.54 (t, *J* = 9.2 Hz, 1H), 3.39 (m, 2H), 3.05 (d, *J* = 9.2 Hz, 1H), 2.04 (m, 1H), 1.98 (m, 1H). ^19^F NMR (400 MHz, DMSO-d_6_) δ -13.82, -136.08.

**(1*R*,4*R*)-5-(3,4-Dimethoxyphenyl)-*N*-(4-fluorophenyl)-2,5-diazabicyclo[2.2.1]heptane-2-carboxamide (2e)**

Prepared by Method A.

White-off solid. Yield 59%. Purity: (LC, *t* _min_ = 3.08) 99.46%. UV max: 210.45, 238.45, 307.45.

MS (ESI) *m*/*z* 372.23 [M + H]^+^; 370.29 [M − H]^−^. LCMS (ESI) *m*/*z* calcd for C_20_H_22_FN_3_O_3_: 370.1567 [M − H]^−^, found: 370.1573 [M − H]^−^. ^1^H NMR (400 MHz, DMSO-d_6_) δ 8.26 (bs, 1H, NH), 7.45 (m, 2H, ArH), 7.02 (dt, *J* = 8.8, 2.0 Hz,2H, ArH), 6.79 (d, *J* = 8.8 Hz, 1H), 6.24 (d, *J* = 2.4 Hz, 1H), 6.08 (dd, *J* = 8.8, 2.4 Hz, 1H), 4.68 (s, 1H), 4.52 (s, 1H), 3.73 (s, 3H, OCH_3_), 3.62 (s, 3H, OCH_3_), 3.55 (dd, *J* =8.8, 1.6 Hz, 1H), 3.41 (s, 2H), 3.01 (d, *J* = 8.8 Hz, 1H), 1.96 (d, *J* = 9.2 Hz, 1H), 1.91 (d, *J* = 9.2 Hz, 1H); ^19^F NMR (400 MHz, DMSO-d_6_) δ -121.36. ^13^C NMR (100 MHz, DMSO-d_6_) δ 152.29 (d, *J*_F_ = 370 Hz), 150.14 (C=O), 150.44, 142.78, 140.80, 137.08, 121.39 (d, *J*_F_ = 8.0 Hz), 115.31 (d, *J*_F_ = 22.0 Hz), 114.92, 104.21, 99.23, 57.88, 57.39, 56.99, 56.64, 55.87, 51.25, 37.30.

**(1*R*,4*R*)-*N*-(3,4-Difluorophenyl)-5-(3,4-dimethoxyphenyl)-2,5-diazabicyclo[2.2.1]heptane-2-carboxamide (2f)**

Prepared by Method A.

Light brown solid. Yield 72%. Purity: (LC, *t* _min_ = 3.21) 99.27%. UVmax: 210.45, 237.45, 309.45. MS (ESI) *m*/*z* 390.19 [M + H]^+^; 388.30 [M − H]^−^. LCMS (ESI) *m*/*z* calcd for C_20_H_21_F_2_N_3_O_3_: 388.1473 [M − H]^−^, found: 388.1482 [M − H]^−^. ^1^H NMR (400 MHz, DMSO-d_6_) δ 8.43 (bs, 1H, NH), 7.63 (m, 1H, ArH), 7.27 (q, *J* = 9.2 Hz, 1H, ArH), 7.21 (m, 1H, ArH), 6.78 (d, *J* = 8.8 Hz, 1H), 6.24 (d, *J* = 2.4 Hz, 1H), 6.09 (dd, *J* = 8.8, 2.4 Hz, 1H), 4.69 (s, 1H), 4.53 (s, 1H), 3.73 (s, 3H, OCH_3_), 3.62 (s, 3H, OCH_3_), 3.56 (dd, *J* = 8.8, 1.6 Hz, 1H), 3.42 (s, 2H), 3.01 (d, *J* = 8.8 Hz, 1H), 1.97 (d, *J* = 9.6 Hz, 1H), 1.91 (d, *J* = 9.6 Hz, 1H). ^19^F NMR (400 MHz, DMSO-d_6_) δ -138.12, -147.64. ^13^C NMR (100 MHz, DMSO-d_6_) δ 153.79 (C=O), 150.44, 148.06 (d, *J*_F_ = 13.0 Hz), 142.73, 140.84, 138.06, 148.06 (d, *J*_F_ = 9.0 Hz), 117.40 (d, *J*_F_ = 18.0 Hz), 115.42 (d, *J*_F_ = 6.0 Hz), 114.90, 108.34 (d, *J*_F_ = 22.0 Hz), 104.23, 99.26, 57.85, 57.36, 56.98, 56.69, 55.87, 51.25, 37.25.

**(1*R*,4*R*)-*N*-(3,4-Difluorophenyl)-5-(pyridin-2-yl)-2,5-diazabicyclo[2.2.1]heptane-2-carboxamide (** **2g)**

Prepared by Method B.

Yield 62% as a white foam.

Purity: (LC, *t* _min_ = 2.08) 97.61%; UV max: 204.45, 239.45, 319.45; MS (ESI) *m*/*z* 329.19 [M − H]^−^; 331.13 [M + H]^+^; LCMS (ESI) *m*/*z* calcd for C_17_H_16_F_2_N_4_O: 331.1370 [M + H]^+^, found: 331.1370 [M + H]^+^; ^1^H NMR (400 MHz, DMSO_6_) δ 8.48 (bs, 1H, NH), 8.07 (dd, *J* = 3.0, 0.6 Hz, 1H, ArH), 7.68 (dq, *J* = 7.6, 2.4 Hz, 1H, ArH), 7.52–7.48 (m, 1H, ArH), 7.31 (q, *J* = 9.2 Hz, 1H, ArH), 7.24 (m, H, ArH), 6.61 (dd, *J* = 6.4, 5.2 Hz, 1H, ArH), 6.55 (d, *J* = 8.4 Hz, 1H, ArH), 4.86 (s, 1H, CH), 4.76 (s, 1H, CH), 3.54–3.49 (m, 2H, CH), 3.38–3.31 (m, 2H, CH), 2.00–1.96 (m, 2H, CH); ^13^C NMR (100 MHz, DMSO-d_6_) δ 157.36 (C=O), 153.92, 148.33, 148.06 (dq, *J*_F_ = 241.0, 13.0 Hz), 138.02 (q, *J*_F_ = 2.0 Hz), 137.74, 117.40 (d, *J*_F_ = 17.0 Hz), 115.55 (q, *J*_F_ = 2.0 Hz), 112.55, 108.42 (d, *J*_F_ = 22.0 Hz), 107.60, 56.76, 56.55, 55.82, 53.06, 37.06; ^19^F NMR (400 MHz, DMSO-d_6_) δ -138.11, -147.56.

**(1*R*,4*R*)-*N*-(7-Chlorobenzo[*c*][1,2,5]oxadiazol-4-yl)-5-(4-methoxyphenyl)-2,5-diazabicyclo[2.2.1]heptane-2-carboxamide (2h)**

Prepared by Method A.

Yellow solid. Yield 70%. MS (ESI) *m*/*z* 398.27 [M − H]^−^. LCMS (ESI) *m*/*z* calcd for C_19_H_18_ClN_5_O_3_: 400.1176 [M + H]^+^, found: 400.1176 [M + H]^+^; 398.1020 [M − H]^−^, found: 398.1046 [M − H]^−^. ^1^H NMR (400 MHz, DMSO-d_6_) δ 7.41 (d, *J* = 8.0 Hz, 1H, ArH), 6.81 (d, *J* = 8.8 Hz, 2H, ArH), 6.79 (bs, 1H, NH), 6.59 (d, *J* = 8.8 Hz, 1H, ArH), 6.28 (d, *J* = 8.0 Hz, 1H, ArH), 4.75 (m, 1H), 4.52 (m, 1H), 3.65 (s, OCH_3_, 3H), 3.63–3.54 (m, 1H), 3.05–3.38 (m, 1H), 3.42 (m, 1H), 3.06–2.99 (m, 1H), 2.08–2.05 (m, 1H), 2.01–1.99 (m, 1H). ^13^C NMR (100 MHz, DMSO-d_6_) δ 151.59 (C=O), 148.95, 145.50, 137.18, 134.87, 115.24 (2C), 114.68 (2C), 114.33, 114.30, 104.86, 102.14, 62.51, 59.96, 56.94, 56.55, 37.08.

**(1*R*,4*R*)-Phenyl 5-(4-methoxyphenyl)-2,5-diazabicyclo[2.2.1]heptane-2-carboxylate (** **2i)**

Prepared by Method A.

Yield 88%, pinkish solid, purity: (LC, *t* _min_ = 3.35) 97.23%; UV max: 191.45, 249.45; MS (ESI) *m*/*z* 325.11 [M + H]^+^; LCMS (ESI) *m*/*z* calcd for C_19_H_20_N_2_O_3_: 325.1552 [M + H]^+^, found: 325.1549 [M + H]^+^; ^1^H NMR (400 MHz, DMSO_6_) δ 7.39–7.32 (m, 2H, ArH), 7.21–7.18 (m, 1H, ArH), 7.13 (d, *J* = 7.6 Hz, 1H, ArH), 7.07 (d, *J* = 7.6 Hz, 1H, ArH), 6.83 (d, *J* = 8.8 Hz, 2H, ArH), 6.60 (d, *J* = 8.8 Hz, 2H, ArH), 4.71 (s, 1H), 4.54 (d, *J* = 3.6 Hz, 1H), 3.67 (s, 3H, OCH_3_), 3.13 (d, J = 8.8 Hz, 1H), 2.03–1.99 (m, 2H, CH); ^13^C NMR (100 MHz, DMSO-d_6_) δ 152.24 (C=O), 151.39, 141.75, 129.70 (2C), 125.71, 122.71 (2C), 122.19 (2C), 115.23 (2C), 114.23, 58.22, 57.54, 56.77, 55.75, 51.41, 37.63.

**(1*R*,4*R*)-5-(4-Methoxyphenyl)-N-phenyl-2,5-diazabicyclo[2.2.1]heptane-2-carboxamide (** **2j)**

Prepared by Method B.

Yield 82%, white solid. Purity: (LC, *t* _min_ = 2.92) 98.59%; UV max: 191.45, 201.45, 241.45; MS (ESI) *m*/*z* 322.86 [M = H]^−^; 324.15 [M + H]^+^; LCMS (ESI) *m*/*z* calcd for C_19_H_21_N_3_O_2_: 324.1712 [M + H]^+^, found: 324.1715 [M + H]^+^; ^1^H NMR (400 MHz, DMSO_6_) δ 8.22 (bs, 1H, NH), 7.45 (d, *J* = 7.8 Hz, 2H, ArH), 7.18 (t, *J* = 7.8 Hz, 2H, ArH), 6.89 (t, *J* = 7.2 Hz, 1H, ArH), 6.81 (d, *J* = 9.2 Hz, 2H, ArH), 6.58 (d, *J* = 9.2 Hz, 2H, ArH), 4.69 (s, 1H), 4.49 (s, 1H), 3.65 (s, 3H, OCH_3_), 3.54 (dd, J = 9.0, 1.2 Hz, 1H), 3.41 (s, 2H), 2.98 (d, *J* = 9.2 Hz, 1H), 1.99 (d, *J* = 9.6 Hz, 1H), 1.92 (d, *J* = 9.6 Hz, 1H); ^13^C NMR (100 MHz, DMSO-d_6_) δ 154.16 (C=O), 151.28, 141.96, 140.76, 128.73 (2C), 122.05, 119.66 (2C), 115.21 (2C), 114.19 (2C), 58.03, 57.36, 56.63, 55.74, 51.04, 37.30.

**(1*R*,4*R*)-*N*-(*tert*-Butyl)-5-(4-methoxyphenyl)-2,5-diazabicyclo[2.2.1]heptane-2-carboxamide (** **2k)**

Prepared by Method B.

Yield 50%, off-white solid. Purity: (LC, *t* _min_ = 2.97) 95.91%; UV max: 199.45, 236.45; MS (ESI) *m*/*z* 340.27 [M − H]^−^; 342.11 [M + H]^+^; LCMS (ESI) *m*/*z* calcd for C_19_H_20_FN_3_O_2_: 342.1618 [M + H]^+^, found: 342.1617 [M + H]^+^; ^1^H NMR (400 MHz, DMSO_6_) δ 8.27 (bs, 1H, NH), 7.47 (m, 2H, ArH), 7.03 (t, *J* = 8.8 Hz, 2H, ArH), 6.81 (d, *J* = 8.8 Hz, 2H, ArH), 6.58 (d, *J* = 8.8 Hz, 2H, ArH), 4.68 (s, 1H, CH), 4.49 (s, 1H, CH), 3.57 (dd, *J* = 9.2, 2.0 Hz, 1H, CH), 3.40 (s, 2H), 2.97 (d, J = 8.8 Hz, 1H), 1.99 (d, *J* = 9.6 Hz, 1H), 1.92 (d, *J* = 9.6 Hz, 1H); ^13^C NMR (100 MHz, DMSO-d_6_) δ 158.85 (d, *J*_F_ = 237 Hz), 154.16 (C=O), 151.28, 141.94, 137.10 (d, *J*_F_ = 3.0 Hz), 121.38 (d, *J*_F_ = 8.0 Hz), 115.31 (2C), 115.21, 115.09, 114.19 (2C), 58.01, 57.35, 56.62, 55.73, 51.01, 37.29; ^19^F NMR (400 MHz, DMSO-d_6_) δ -121.86.

**(1*R*,4*R*)-*N*-(3,4-Difluorophenyl)-5-(4-fluorophenyl)-2,5-diazabicyclo[2.2.1]heptane-2-carboxamide (** **2l)**

Prepared by Method B.

Yield 67%, off-white solid. Purity: (LC, *t* _min_ = 3.27) 96.89%; UV max: 204.45, 240.45; MS (ESI) *m*/*z* 346.20 [M − H]^−^; 348.09 [M + H]^+^; LCMS (ESI) *m*/*z* calcd for C_18_H_16_F_3_N_3_O: 348.1324 [M + H]^+^, found: 348.1326 [M + H]^+^; ^1^H NMR (400 MHz, DMSO_6_) δ 8.49 (bs, 1H, NH), 7.65 (dq, *J* = 7.6, 2.4 Hz, 1H, ArH), 7.26 (q, *J* = 10.4 Hz, 1H, ArH), 7.23 (m, 1H, ArH), 7.02 (t, *J* = 9.2 Hz, 2H, ArH), 6.62 (m, 2H, ArH), 4.72 (s, 1H, CH), 4.57 (s, 1H, CH), 3.58 (dd, *J* = 9.2, 1.6 Hz, 1H, CH), 3.46–3.40 (m, 2H), 3.02 (d, *J* = 8.8 Hz, 1H), 2.00 (d, *J* = 9.2 Hz, 1H), 1.94 (d, *J* = 9.2 Hz, 1H); ^19^F NMR (400 MHz, DMSO-d_6_) δ -129.38, -138.11, -147.59.

**(1*R*,4*R*)-2-(4-Fluorophenyl)-2,5-diazabicyclo[2.2.1]heptane (19R)**

Yield 47%. Yellowish foam. Purity: (LC, *t* _min_ = 3.27) 96.89%; UV max: 204.45, 240.45; MS (ESI) *m*/*z* 346.20 [M − H]^−^; 348.09 [M + H]^+^; LCMS (ESI) *m*/*z* calcd for C_18_H_16_F_3_N_3_O: 348.1324 [M + H]^+^, found: 348.1326 [M + H]^+^; ^1^H NMR (400 MHz, DMSO_6_) δ 8.49 (bs, 1H, NH), 7.65 (dq, *J* = 7.6, 2.4 Hz, 1H, ArH), 7.26 (q, *J* = 9.2 Hz, 1H, ArH), 7.24–7.22 (m, 1H, AH), 7.02 (t, *J* = 6.4 Hz, ArH), 6.64–6.60 (m, 2H, ArH), 4.72 (s, 1H, CH), 4.60 (s, 1H, CH), 3.58 (dd, *J* = 9.2, 1.6 Hz, 1H, CH), 3.46–3.40 (m, 2H), 3.02 (d, *J* = 8.8 Hz, 1H), 2.00 (d, *J* = 9.2 Hz, 1H), 1.95 (d, *J* = 9.2 Hz, 1H); ^13^C NMR (100 MHz, DMSO-d_6_) δ 156.05 (C=O), 153.82, 150.46 (dd, *J*_F_ = 240, 13 Hz), 145.95 (dd, *J*_F_ = 238, 13 Hz), 144.18, 138.00 (d, *J*_F_ = 3 Hz), 117.22 (d, *J*_F_ = 18 Hz), 116.05 (2C), 115.83 (2C), 115.46 (q, *J*_F_ = 4 Hz), 113.96 (d, *J*_F_ = 22 Hz), 57.87, 57.34, 56.68, 37.29; ^19^F NMR (400 MHz, DMSO-d_6_) δ -129.38, -138.11, -147.59.

**(1*R*,4*R*)-*N*-(2,4-Difluorophenyl)-5-(4-methoxyphenyl)-2,5-diazabicyclo[2.2.1]heptane-2-carboxamide (** **2m)**

Prepared by Method B.

Yield 87%, pinkish solid. Purity: (LC, *t* _min_ = 2.97) 99.34%; UV max: 196.45, 231.45; MS (ESI) *m*/*z* 358.15 [M − H]^−^; 360.12 [M + H]^+^; LCMS (ESI) *m*/*z* calcd for C_18_H_16_F_3_N_3_O: 360.1524 [M + H]^+^, found: 360.1493 [M + H]^+^; ^1^H NMR (400 MHz, DMSO_6_) δ 8.49 (bs, 1H, NH), 7.65 (dq, J = 7.6, 2.4 Hz, 1H, ArH), 7.26 (q, *J* = 9.2 Hz, 1H, ArH), 7.24–7.22 (m, 1H, AH), 7.02 (t, J = 6.4 Hz, ArH), 6.64–6.60 (m, 2H, ArH), 4.72 (s, 1H, CH), 4.60 (s, 1H, CH), 3.58 (dd, *J* = 9.2, 1.6 Hz, 1H, CH), 3.46–3.40 (m, 2H), 3.02 (d, *J* = 8.8 Hz, 1H), 2.00 (d, *J* = 9.2 Hz, 1H), 1.95 (d, *J* = 9.2 Hz, 1H); ^13^C NMR (100 MHz, DMSO-d_6_) δ 154.31 (C=O), 151.27, 141.91, 128.15 (d, *J*_F_ = 10 Hz), 124.08 (dd, *J*_F_ = 12.0, 3.0 Hz), 115.21 (2C), 114.14 (2C), 111.24 (dd, *J*_F_ = 22.0, 3.0 Hz), 104.36 (t, *J*_F_ = 26 Hz), 57.28, 56.83, 55.73, 51.05, 37.39; ^19^F NMR (400 MHz, DMSO-d_6_) δ -115.56, -117.97.

**(1*R*,4*R*)-*N*,5-bis(4-Methoxyphenyl)-2,5-diazabicyclo[2.2.1]heptane-2-carboxamide (2n)**

Prepared by Method B.

Yield 60%. off-white solid. Purity: (LC, *t* _min_ = 2.86) 97.29%; UV max: 199.45, 244.45; MS (ESI) *m*/*z* 352.80 [M − H]^−^; 354.17 [M + H]^+^; LCMS (ESI) *m*/*z* calcd for C_20_H_23_N_3_O_3_: 360.1524 [M + H]^+^, found: 354.1818 [M + H]^+^; ^1^H NMR (400 MHz, DMSO_6_) δ 8.07 (bs, 1H, NH), 7.65 (d, *J* = 9.2 Hz, 2H, ArH), 6.80 (d, *J* = 9.2 Hz, 1H, ArH), 6.77 (d, *J* = 9.2 Hz, 1H, ArH), 6.58 (d, *J* = 9.2 Hz, 1H, ArH), 4.66 (s, 1H, CH), 4.48 (s, 1H, CH), 3.68 (s, 3H, OCH_3_), 3.65 (s, 3H, OCH_3_), 3.55 (dd, *J* = 9.0, 1.6 Hz, 1H, CH), 3.38 (m, 2H), 2.96 (d, *J* = 8.8 Hz, 1H), 1.97 (d, *J* = 8.8 Hz, 1H), 1.91 (d, *J* = 9.2 Hz, 1H); ^13^C NMR (100 MHz, DMSO-d_6_) δ 154.74 (C=O), 154.40, 151.26, 141.97, 133.74, 121.57 (2C), 115.21, 114.17, 113.90 (2C), 58.02, 57.38, 56.55, 55.74, 55.51, 50.97, 37.32.

**(1*R*,4*R*)-*N*-(4-Cyanophenyl)-5-(4-methoxyphenyl)-2,5-diazabicyclo[2.2.1]heptane-2-carboxamide (2o)**

Prepared by Method B.

Yield 90.0%. off-white solid. Purity: (LC, *t* _min_ = 2.95) 98.71%; UV max: 199.45, 268.45; MS (ESI) *m*/*z* 347.24 [M − H]^−^; 349.14 [M + H]^+^; LCMS (ESI) *m*/*z* calcd for C_20_H_20_N_4_O_2_: 349.1665 [M + H]^+^, found: 349.1663 [M + H]^+^; ^1^H NMR (400 MHz, DMSO_6_) δ 8.71 (bs, 1H, NH), 7.69 (d, *J* = 8.8 Hz, 2H, ArH), 7.64 (d, *J* = 8.8 Hz, 2H, ArH), 6.81 (d, *J* = 8.8 Hz, 2H, ArH), 6.59 (d, *J* = 8.8 Hz, 2H, ArH), 4.73 (s, 1H, CH), 4.51 (s, 1H, CH), 3.65 (s, 3H, OCH_3_), 3.57 (dd, *J* = 8.8, 1.6 Hz, 1H, CH), 3.37 (m, 2H), 2.98 (d, *J* = 9.2 Hz, 1H), 1.99 (d, *J* = 7.6 Hz, 1H), 1.93 (d, *J* = 7.6 Hz, 1H); ^13^C NMR (100 MHz, DMSO-d_6_) δ 153.40 (C=O), 151.34, 145.39, 141.86, 133.29 (2C), 119.87, 119.10 (2C), 115.21 (2C), 114.22 (2C), 103.33, 57.99, 57.28, 56.83, 55.73, 51.14, 37.22.

**(1*R*,4*R*)-5-(4-Methoxyphenyl)-*N*-(4-(trifluoromethyl)phenyl)-2,5-diazabicyclo[2.2.1]heptane-2-carboxamide (** **2p)**

Prepared by Method B.

Yield 89%. off-white foam. Purity: (LC, *t* _min_ = 3.36) 98.47%; UV max: 204.45, 251.45; MS (ESI) *m*/*z* 390.13 [M − H]^−^; 392.13 [M + H]^+^; LCMS (ESI) *m*/*z* calcd for C_20_H_20_F_3_N_3_O_2_: 392.1586 [M + H]^+^, found: 392.1597 [M + H]^+^; ^1^H NMR (400 MHz, DMSO_6_) δ 8.63 (bs, 1H, NH), 7.71 (d, *J* = 8.8 Hz, 2H, ArH), 7.57 (d, *J* = 8.8 Hz, 2H, ArH), 6.82 (d, *J* = 9.2 Hz, 2H, ArH), 6.59 (d, *J* = 9.2 Hz, 2H, ArH), 4.74 (s, 1H, CH), 4.51 (s, 1H, CH), 3.66 (s, 3H, OCH_3_), 3.58 (dd, *J* = 9.2, 1.6 Hz, 1H, CH), 3.45 (m, 2H), 2.99 (d, *J* = 8.8 Hz, 1H), 1.99 (d, *J* = 9.2 Hz, 1H), 1.94 (d, *J* = 9.2 Hz, 1H); ^13^C NMR (100 MHz, DMSO-d_6_) δ 153.68 (C=O), 151.38, 144.63, 141.90, 126.06, 126.03, 125.06 (q, *J* = 270 Hz), 122.04 (q, *J* = 31 Hz), 118.98, 115.21, 114.21, 58.01, 57.31, 56.77, 55.73, 51.11, 37.11; ^19^F NMR (400 MHz, DMSO-d_6_) δ -60.03.

**(1*R*,4*R*)-*N*-(*tert*-Butyl)-5-(4-methoxyphenyl)-2,5-diazabicyclo[2.2.1]heptane-2-carboxamide (** **2q)**

Prepared by Method B.

Yield 85.0%, white solid.

Purity: (LC, *t* _min_ = 2.86) 99.36%; UV max: 201.45, 249.45; MS (ESI) *m*/*z* 304.14 [M + H]^+^; LCMS (ESI) *m*/*z* calcd for C_17_H_25_N_3_O_2_: 304.2025 [M + H]^+^, found: 304.2027 [M + H]^+^; ^1^H NMR (400 MHz, DMSO_6_) δ 6.80 (d, *J* = 8.8 Hz, 2H, ArH), 6.53 (d, *J* = 8.8 Hz, 2H, ArH), 5.41 (bs, 1H, NH), 4.52 (s, 1H, CH), 4.39 (s, 1H, CH), 3.65 (s, 3H, OCH_3_), 3.47 (dd, *J* = 8.8, 1.6 Hz, 1H, CH), 3.22 (s, 2H), 2.85 (d, *J* = 8.8 Hz, 1H), 1.88 (d, *J* = 8.8 Hz, 1H), 1.79 (d, *J* = 8.8 Hz, 1H), 1.20 (s, 9H, (CH_3_)_3_); ^13^C NMR (100 MHz, DMSO-d_6_) δ 156.31 (C=O), 151.14, 142.06, 115.18 (2C), 114.04 (2C), 57.84, 57.35, 56.22, 55.73, 50.65, 50.29, 37.41, 29.67 (3C).

**(1*R*,4*R*)-Cyclohexyl 5-(4-methoxyphenyl)-2,5-diazabicyclo[2.2.1]heptane-2-carboxylate (** **2r)**

Prepared by Method A.

Yield 96.0%, white solid.

Purity: (LC, *t* _min_ = 3.66) 98.75%; UV max: 249.45; MS (ESI) *m*/*z* 331.19 [M + H]^+^; LCMS (ESI) *m*/*z* calcd for C_19_H_26_N_2_O_3_: 331.2022 [M + H]^+^, found: 331.2023 [M + H]^+^; ^1^H NMR (400 MHz, DMSO_6_) δ 6.80 (d, *J* = 8.8 Hz, 2H, ArH), 6.56 (d, *J* = 8.8 Hz, 2H, ArH), 4.51 (m, 1H, CH), 4.40 (s, 1H, CH), 3.65 (s, 3H, OCH_3_), 3.47 (m, 1H, CH), 3.29 (m, 2H), 2.88 (m, 1H), 1.92 (m, 2H), 1.73–1.62 (m, 2H), 1.65–1.52 (m, 2H), 1.43–1.28 (m, 6H); ^13^C NMR (100 MHz, DMSO-d_6_) δ 154.15 (C=O), 151.30, 141.82, 115.18 (2C), 114.12 (2C), 72.44, 58.18, 57.68, 57.29, 56.89, 56.75, 55.72, 50.96, 37.52, 31.94, 25.40, 23.51.

**(1*R*,4*R*)-*N*-(5-Chloro-2-methoxyphenyl)-5-(3,4-dimethoxyphenyl)-2,5-diazabicyclo[2.2.1] heptane-2-carboxamide (2s)**

Prepared by Method A.

Off-white solid. Yield 63%. Purity: (LC, *t* _min_ = 3.43) 96.14%. UV max: 214.45, 248.45, 289.45. MS (ESI) *m*/*z* 418.19 [M + H]^+^; 416.30 [M − H]^−^. LCMS (ESI) *m*/*z* calcd for C_21_H_24_ClN_3_O_4_: 416.1377 [M − H]^−^, found: 416.1356 [M − H]^−^. ^1^H NMR (400 MHz, DMSO-d_6_) δ 7.87 (bs, 1H, NH), 7.35 (m, 1H, ArH), 6.97 (m, 2H), ArH), 6.78 (d, *J* = 9.2 Hz, 1H, ArH), 7.21 (m, 1H, ArH), 6.78 (d, *J* = 8.8 Hz, 1H), 6.24 (d, *J* = 2.4 Hz, 1H), 6.09 (dd, *J* = 8.8, 2.4 Hz, 1H), 4.65 (s, 1H), 4.52 (s, 1H), 3.77 (s, 3H, OCH_3_), 3.72 (s, 3H, OCH_3_), 3.62 (s, 3H, OCH_3_), 3.54 (d, J =7.6 Hz, 1H), 3.47 (d, J =8.8 Hz, 1H), 3.39 (d, *J* = 9.2 Hz, 1H), 3.03 (d, *J* = 9.2 Hz, 1H), 1.96 (d, J = 9.2 Hz, 1H), 1.92 (d, *J* = 9.2 Hz, 1H). ^13^C NMR (100 MHz, DMSO-d_6_) δ 153.46 (C=O), 150.45, 148.10, 142.63, 140.83, 130.18, 124.32, 122.31, 120.05, 114.89, 112.45, 104.16, 99.25, 57.59, 57.42, 56.97, 56.80, 56.52, 55.89, 51.05, 37.37.

***N*** **-(3,4-Difluorophenyl)-3-(4-fluorophenyl)-1*H*-pyrrole-1-carboxamide (3a)**

Prepared by Method A.

Yield 82%. Off-white solid. Purity: (LC, *t* _min_ = 3.72) 98.64%. UV max: 222.45. MS (ESI) *m*/*z* 317.10 [M + H]^+^; 315.34 [M − H]^−^. LCMS (ESI) *m*/*z* calcd for C_17_H_11_F_3_N_2_O: 317.0902 [M + H]^+^; Found: 317.0922 [M + H]^+^. ^1^H NMR (400 MHz, CDCl_3_) δ 7.53–7.50 (*m*, 1H, ArH), 7.45–7.42 (*m*, 3H, ArH), 7.32 (bs, 1H, NH), 7.26 (*m*, 1H, ArH), 7.10–7.08 (m, 2H), 7.01 (*t*, *J* = 8.8 Hz, 2H, ArH), 6.55 (dd, *J* = 3.2, 1.6 Hz, 1H, ArH). ^19^F NMR (400 MHz, CDCl_3_) δ -115.55, -134.87, -141.50.

***N*-(3,4-Difluorophenyl)-4-(4-methoxyphenyl)-1*H*-pyrazole-1-carboxamide (3b)**

Prepared by Method A.

Yield 67%. White solid. Purity: (LC, *t* _min_ = 2.67) 97.06%. UV max: 252.45. MS (ESI) *m*/*z* 319.13 [M − H]^−^. ^1^H NMR (400 MHz, CDCl_3_) δ 10.76 (bs, 1H, NH), 8.83 (s, 1H), 8.38 (s, 1H), 7.92–7.87 (*m*, 1H, ArH), 7.75 (d, *J* = 8.4 Hz, 2H, ArH), 7.66–7.64 (*m*, 1H, ArH), 7.51–7.44 (*m*, 1H, ArH), 6.99 (d, *J* = 8.4 Hz, 2H, ArH), 3.79 (s, 3H, OCH_3_). ^19^F NMR (400 MHz, CDCl_3_) δ -137.23, -143.97.

**1,3-bis(3,4-Difluorophenyl)urea (4a)**

Prepared by Method A and B.

Yield 86%. White solid. MS (ESI) *m*/*z* 285.02 [M + H]^+^; 283.10 [M − H]^−^. LCMS (ESI) *m*/*z* calcd for C_19_H_19_F_2_N_3_O_2_: 360.1524 [M + H]^+^; Found: 360.1536 [M + H]^+^ and 358.1447 [M − H]^−^. ^1^H NMR (400 MHz, CDCl_3_) δ 8.961 (bs, 2H, NH), 7.66–7.06 (m, 2H, ArH), 7.35 (*q*, *J* = 9.2 Hz, 2H, ArH), 7.14–7.11 (m, 2H, ArH). ^13^C NMR (100 MHz, CDCl_3_) δ 152.85 (N-*C*O-N), 150.79 (dd, *J* = 450, 12 Hz, 2C), 148.38 (dd, *J* = 488, 13 Hz, 2C), 137.03 (q, *J* = 2 Hz, 2C), 107.94 (d, *J* = 17 Hz, 2C), 137.03 (q, *J* = 3 Hz, 2C), 107.96 (d, *J* = 21 Hz, 2C). ^19^F NMR (CDCl*_3_*, 400 MHz) δ -137.44, -146.83.

**1-(3,4-Difluorophenyl)-3-(3,4,5-trifluorophenyl)urea (4b)**

Prepared by Method A.

Yield 82%. White solid. Purity: (LC, *t* _min_ = 3.68) 99.35%; MS (ESI) *m*/*z* 303.09 [M + H]^+^; 301.16 [M − H]^−^; LCMS (ESI) *m*/*z* calcd for C_13_H_7_ F_6_N_2_O: 303.0557 [M + H]^+^, found: 303.0566 [M + H]^+^. ^1^H NMR (400 MHz, CDCl_3_) δ 9.19 (bs, 1H, NH), 9.16 (bs, 1H, NH), 7.69–7.61 (m, 1H, ArH), 7.40–7.37 (m, 3H), 7.15–7.12 (m, 1H). ^13^C NMR (100 MHz, CDCl_3_) δ 152.71 (C=O), 148.38, 140.67, 140.56, 130.92, 117.98, 117.79, 116.73, 111.87, 108.07, 107.85, 106.99, 106.74.

**1-(4-Chloro-3-fluorophenyl)-3-(3,4-difluorophenyl)urea (4c)**

Prepared by Method A.

Yield 82%. White-off solid. Purity (LC, *t* _min_ = 3.70) 98.65%. UV 210.45, 257.45 nm. MS (ESI) *m*/*z* 301.06 [M + H]^+^; 299.18 [M − H]^−^. LCMS (ESI) *m*/*z* calcd for C_13_H_6_ClFN_2_O: 301.0356. Found: 301.0373 [M + H]^+^. ^1^H NMR (400 MHz, CDCl_3_) δ 9.11 (bs, 1H, NH), 9.02 (bs, 1H, NH), 7.66–7.60 (m, 2H, ArH), 7.46 (t, *J* = 8.6 Hz, 1H), 738–7.31 (m, 1H), 7.18 (dd, *J* = 8.8, 1.6 Hz, 1H), 7.17 (m, 1H). ^13^C NMR (100 MHz, CDCl_3_) δ 152.71 (C=O), 148.38, 140.67, 140.56, 130.92, 117.98, 117.79, 116.73, 111.87, 108.07, 107.85, 106.99, 106.74.

**1,3-bis(3,4,5-Trifluorophenyl)urea (4d)**

Prepared by Method A.

Yield 80%. White solid. Purity: (LC, *t* _min_ = 3.62) 97.27%. UV max: 253.45, 190.45; MS (ESI) *m*/*z* 321.04 [M + H]^+^; 319.20 [M − H]^−^. LCMS (ESI) *m*/*z* calcd for C13H6F6N2O: 319.0306 [M − H]^−^, found: 319.0314 [M − H]^−^. ^1^H NMR (400 MHz, DMSO-d_6_) δ 9.22 (bs, 2H, NH), 7.37 (dd, *J* = 10.4, 6.4 Hz,4H, ArH). ^13^C NMR (100 MHz, DMSO-d_6_) δ 152.60 (NH*C*(=O)NH), 151.83 (m, 2C), 149.40 (m, 2C), 136.31 (2C), 136.16 (m, 2C), 103.38 (d, *J* = 24 Hz, 4C). ^19^F NMR (DMSO-d_6_, 400 MHz) δ -135.09 (4F), -169.90 (2F).

**1-(3,4-Difluorophenyl)-3-(4-methoxyphenyl)urea (4e)**

Prepared by Method A.

Yield 71%. Light brown solid. UV max: 190.45, 223.45. Purity (LC, *t* _min_ = 3.08): 95.57%.

MS (ESI) *m*/*z* 279.17 [M + H]^+^; 277.25 [M − H]^−^. LCMS (ESI) *m*/*z* calcd for C_14_H_12_F_2_N_2_O_2_: 277.0789 [M − H]^−^; Found: 277.0796 [M − H]^–^; 279.0957 [M + H]^+^. ^1^H NMR (400 MHz, DMSO-d_6_) δ 8.80 (bs, 1H, NH), 8.53 (bs, 1H, NH), 7.65 (*m*, 1H, ArH), 7.08 (d, *J* = 9.0 Hz, 2H, ArH), 7.07 (m, 1H, ArH), 7.09 (m, 1H, ArH), 6.85 (d, *J* = 9.0 Hz, 2H, ArH), 3.70 (s, 3H, OCH3). ^19^F NMR (DMSO-d_6_, 400 MHz) δ -137.57, -147.50.

**1,3-bis(4-Methoxyphenyl)urea (4f)**

Prepared by Method A.

Yield 81%. White-off solid. Purity: (LC, *t* _min_ = 2.80) 97.78%. UV max: 190.45, 223.45. MS (ESI) *m*/*z* 273.17 [M + H]^+^; 295.14 [M + Na]^+^. LCMS (ESI) *m*/*z* calcd for C_15_H_16_N_2_O_3_: 271.1083 [M − H]^−^; Found: 271.1084 [M − H]^–^; 273.1024 [M + H]^+^. ^1^H NMR (400 MHz, CDCl_3_) δ 9.34 (bs, 2H, NH), 7.22 (d, *J* = 8.4 Hz, 4H, ArH), 6.88 (d, *J* = 8.4 Hz, 4H, ArH), 3.80 (s, 6H, 2(OCH_3_)).

***N*** **-(3,4-Difluorophenyl)-4-fluoro-1*H*-indole-1-carboxamide (4g)**

Prepared by Method A.

Yield 77%. Yellowish solid. Purity: (LC, *t* _min_ = 3.66) 97.56%. UV max: 190.45, 223.45.

MS (ESI) *m*/*z* 291.13 [M + H]^+^; 289.15 [M − H]^−^. LCMS (ESI) *m*/*z* calcd for C_15_H_9_F_3_N_2_O: 289.0589 [M − H]^–^; Found: 289.0588 [M − H]^–^; 291.0719 [M + H]^+^. ^1^H NMR (400 MHz, CDCl_3_) δ 7.82 (*d*, *J* = 8.4 Hz, 1H, ArH), 7.54 (*dd*, *J* = 6.8, 1.6 Hz, 1H, ArH), 7.44 (*d*, *J* = 4.0 Hz, 1H, ArH), 7.43 (bs, 1H, NH), 7.23 (m, 1H, ArH), 7.10 (m, 2H, ArH), 6.89 (*t*, *J* = 8.8 Hz, 1H, ArH), 6.73 (d, *J* = 3.8 Hz, 1H, ArH). ^19^F NMR (CDCl*_3_*, 400 MHz) δ -121.08, -134.93, -141.53.

**4-Cyano-N-(3,4-difluorophenyl)-1*H*-indole-1-carboxamide (4h)**

Prepared by Method A.

Yield 81%. White solid. Purity (LC, *t* _min_ = 3.58) 96.39%. MS (ESI) *m*/*z* 298.13 [M + H]^+^; 296.38 [M − H]^−^. LCMS (ESI) *m*/*z* calcd for C_16_H_9_F_2_N_3_O: 296.0635 [M − H]^−^; Found: 296.0674 [M − H]^−^. ^1^H NMR (400 MHz, DMSO-d*_6_*) δ 10.49 (bs, 1H, NH), 8.54 (*d*, *J* = 8.4 Hz, ArH), 8.29 (*d*, *J* = 3.6 Hz, ArH), 7.81 (m, 1H, ArH), 7.77 (*d*, *J* = 8.4 Hz, 1H, ArH), 7.51 (*d*, *J* = 8.4 Hz, 1H, ArH), 7.47 (m, 2H, ArH), 6.96 (*d*, *J* = 3.6 Hz, ArH). ^13^C NMR (100 MHz, DMSO-d*_6_*) δ 149.66 (N-*C*O-N), 149.47 (dd, *J*_F-F_ = 243, 13 Hz), 146.45 (dd, *J*_F-F_ = 241, 12 Hz), 135.53, 131.38, 129.30, 127.84, 124.64, 120.57, 128.24, 128.09 (d, *J*_F-F_ = 8 Hz), 127.75 (q, *J*_F-F_ =3 Hz), 110.52, 110.31, 104.61, 102.86. ^19^F NMR (CDCl*_3_*, 400 MHz) δ -137.12, -143.87.

**3,4-Difluorophenyl (3,4-difluorophenyl)carbamate** (**4i**)

Under an argon atmosphere, to a solution of triphosgene (1.48 g, 5 mmol) in 10 mL of anhydrous THF, 3,4-difluorophenol (650 mg, 5 mmol) in 3 mL of THF was added slowly at 0 °C to the solution, and triethylamine (0.7 mL, 5 mmol) was added dropwise to the solution. The resulting mixture was stirred at the same temperature for 10 min and allowed to heat to reflux for 30 min. After cooling the flask, 3,4-difluoroaniline (645 mg, 5 mmol) was added to the solution and stirred overnight at room temperature. The reaction mixture was concentrated under reduced pressure and then dissolved into 50 mL of ethyl acetate, washed with water (50 mL), saturated NaHCO_3_ (20 mL), water (30 mL), 3 *N* HCl (20 mL), and water (30 mL). The organic layer was dried over anhydrous MgSO_4,_ and concentrated under reduced pressure and purified with flash column chromatography using EtOAc/hexane (1/3, *v*/*v*) as an eluent to produce the desired product. Yield 73% as a white solid. Purity (LC, *t* _min_ = 3.35): 98.37%; UV max: 190.45, 230.45; MS (ESI) *m*/*z* 285.99 [M + H]^+^, 283.95 [M − H]^−^; LCMS (ESI) *m*/*z* calcd for C_13_H_7_F_4_NO_2_: 286.0491 [M + H]^+^, found: 286.0479 [M + H]^+^. ^1^H NMR (400 MHz, CDCl_3_) δ 7.48 (t, *J* = 8.8 Hz, 1H, NH), 7.18 (q, *J* = 8.8 Hz, 1H, ArH), 7.10–7.08 (m, 1H, ArH), 7.08–7.06 (m, 1H, ArH), 7.06–7.02 (m, 1H, ArH), 6.97–6.95 (s, 2H, ArH). ^19^F NMR (400 MHz, CDCl_3_) δ -134.33, -135.04, -140.65, -142.79.

***N*** **,2-bis(3,4-Difluorophenyl)acetamide (4j)**

Yield 70% as a white solid. Purity (LC, t*_R_* 3.28 min): 96.97%; UV max: 221.45; MS (ESI) *m*/*z* 283.98 [M + H]^+^; 282.06 [M − H]^−^; LCMS (ESI) *m*/*z* calcd for C_14_H_9_F_4_NO: 284.0699 [M + H]^+^, found: 284.0691 [M + H]^+^. ^1^H NMR (400 MHz, DMSO-d_6_) δ 10.43 (bs, 1H), 8.11 (dq, *J* = 7.6, 2.4 Hz, 1H), 7.43–7.36 (m, 3H), 7.30–7.27 (m, 1H), 7.17–7.14 (m, 1H), 3.68 (s, CH_3_, 2H). ^13^C NMR (100 MHz, DMSO-d_6_) δ 169.23 (C=O), 150.78 (dd, *J*_C-F_ = 244.0, 12.5 Hz), 150.60 (dd, *J*_C-F_ = 46.0, 12.5 Hz), 148.19 (dd, *J*_C-F_ = 46.0, 12.5 Hz), 146.92 (dd, *J*_C-F_ = 240.0, 12.5 Hz), 136.59 (q, *J*_C-F_ = 6.0, 3.0 Hz), 133.66 (q, *J*_C-F_ = 6.0, 3.0 Hz), 126.64 (q, *J*_C-F_ = 6.0, 3.0 Hz), 118.84 (d, *J*_C-F_ = 17.0 Hz), 118.84 (d, *J*_C-F_ = 17.0 Hz), 118.04 (d, *J*_C-F_ = 17.0 Hz), 117.72 (d, *J*_C-F_ = 17.0 Hz), 115.87 (q, *J*_C-F_ = 3.0 Hz), 108.65 (d, *J*_C-F_ = 21.0 Hz), 42.27. ^19^F NMR (400 MHz, DMSO-d_6_) δ -137.14, -139.25, -141.79, -144.74.

***S*-(3,4-difluorophenyl) (3,4-difluorophenyl)carbamothioate** (**4k**)

Under an argon atmosphere, to a solution of triphosgene (594 mg, 2 mmol) in 10 mL of anhydrous THF, 3,4-difluorothiophenol (292 mg, 2 mmol) in 3 mL of THF was added slowly at 0 °C to the solution, and triethylamine (0.42 mL, 3 mmol) was added dropwise to the solution. The resulting mixture was stirred at the same temperature for 10 min and allowed to heat to reflux for 30 min. After cooling the flask, the solution of 3,4-difluoroaniline (258 mg, 2 mmol) in 2 mL anhydrous THF was added to the mixture and stirred overnight at room temperature. The reaction mixture was concentrated under reduced pressure and then dissolved into 30 mL of ethyl acetate and washed with water (30 mL) and brine (30 mL). The organic layer was dried over anhydrous MgSO_4,_ and concentrated under reduced pressure and purified with flash column chromatography using EtOAc/hexane (1/3, *v*/*v*) as an eluent to produce the desired product.

Yield 89%. White solid. Purity: 97.90%; UV max: 195.45, 248.45 nm; MS (ESI) *m*/*z* 302.02 [M + H]^+^; LCMS (ESI) *m*/*z* calcd for C_13_H_7_F_4_NOS: 302.0263 [M + H]^+^, found: 302.0260 [M + H]^+^. ^1^H NMR (400 MHz, CDCl_3_) δ 7.49–7.40 (m, 1H, ArH), 7.33–7.30 (m, 1H, ArH), 7.27–7.21 (m, 1H, ArH), 7.18 (bs, 1H, NH), 7.12 (q, *J* = 9.2 Hz, 1H, ArH), 7.01–6.99 (m, 1H, ArH); ^19^F NMR (400 MHz, CDCl_3_) δ -134.33, -135.04, -140.65, -142.79.

**bis((1*R*,4*R*)-5-(4-Methoxyphenyl)-2,5-diazabicyclo[2.2.1]heptan-2-yl)methanone (5)**

A 100 mL, oven-dried, two-necked, round-bottomed flask is charged with a Teflon-coated magnetic oval stir bar and coupled with a 50 mL dropping funnel. Both the dropping funnel and the round-bottomed flask are sealed with a rubber septum. Under a nitrogen atmosphere, compound **15R** (312 mg, 1.52 mmol) in 3 mL of THF was added slowly at 0 °C to a solution of triphosgene (226 mg, 0.76 mmol) in 10 mL of dry THF. The resulting mixture was stirred at the same temperature for 10 min and allowed to stir at room temperature for another 30 min. After completion of the reaction, the reaction mixture was added to crushed ice and extracted with ethyl acetate. The organic layer was dried over anhydrous MgSO_4_ and concentrated under reduced pressure to purify by silica gel chromatography (EtOAc/n-hexane = 1:1) or (hexane/acetone = 3:1, *v*/*v*) to afford the target compound as a yellowish solid. (**5**, Yield 73%). Purity: (LC, *t* _min_ = 3.13) 97.64%. MS (ESI) *m*/*z* 435.31 [M + H]^+^. LCMS (ESI) *m*/*z* calcd for C_25_H_30_N_4_O_3_: 435.2396 [M + H]^+^; Found: 435.2378 [M + H]^+^. ^1^H NMR (400 MHz, DMSO-d_6_) δ 6.78 (*d*, *J* = 8.6 Hz, 4H, ArH), 6.50 (*d*, *J* = 8.6 Hz, 4H, ArH), 4.35 (s, 2H), 4.19 (s, 2H), 3.65 (s, 6H, (OCH_3_)2), 3.45 (d, *J* = 8.2 Hz, 2H), 3.32 (d, *J* = 8.2 Hz, 2H), 3.01 (d, *J* = 8.8 Hz, 2H), 3.00 (d, *J* = 8.8 Hz, 2H), 1.80 (bs, 4H). ^13^C NMR (100 MHz, DMSO-d6) δ 159.85 (N-*C*O-N), 151.13 (2C), 141.87 (2C), 115.21 (4C), 113.99 (4C), 58.02 (2C), 57.36 (2C), 56.57 (2C), 55.72 (2C, (O*C*H_3_)_2_), 52.69 (2C), 36.39 (2C).

## 4. Conclusions

We have designed 4 different series of PFI-3 analogs (**2**, **3**, **4**, and **5**) and synthesized them to examine the activity of each to sensitize GBM cells to TMZ-induced cell death to find the best treatment for GBM. We first optimized the A-ring of series **2**, followed by finding the optimum chirality of the bicyclic ring system, which was the R-isomer. This was followed by replacing the bicyclic ring system with a 5-membered linker in series **3**, which provided weak activity compared to PFI-3. We also examined the di-phenyl urea compounds of **4**, which demonstrated much better action on bromodomain inhibitory activity as compared to PFI-3, especially **4a** (difluoro phenyl analogs on the A- and B-rings). Finally, methoxyphenyl-B-ring with linker PFI-3 analog **5** showed exceptionally strong activity as a bromodomain inhibitor. Overall, reconfiguration of A- and B-ring substituents in the initial lead compound **2a** and optimization of A- and B-rings resulted in a significant boost to bromodomain inhibitor activity for enhancing TMZ in treating GBM. We have retained pharmaceutically druggable properties as potential drug candidates for GBM treatment as calculated by ADME using SwissADME, as shown in Figure 5. Notably, this progression was achieved in rational design, synthesis, and proper pharmaceutical tests for possible new drugs like **2a**, **2b**, **2c**, **4a**, and **5** for enhancing the action of TMZ in treating GBM.

## Figures and Tables

**Table 1:** Summarized physicochemical, pharmacokinetic, and drug likeness properties of **2a**–**c**, **2as**, **3b**, **4a**, **4i**–**k**, and **5a** using the Swiss ADME web tool.

ID	Physicochemical Parameters						Pharmacokinetic Parameters		Drug-Likeness Properties	
	HA	RB	HBA	HBD	FC	MLOGP	GI Absorption	BBB Permeation	Lipinski’s Rules of Five	Bio- Availability
**2a**	26	5	4	1	0.32	3.38	High	Yes	Yes 0 violation	0.55
**2as**	26	5	4	1	0.32	3.38	High	Yes	Yes 0 violation	0.55
**2b**	26	5	5	0	0.32	3.38	High	Yes	Yes 0 violation	0.55
**2c**	26	5	4	0	0.35	3.36	High	Yes	Yes 0 violation	0.55
**3a**	23	4	1	4	0	4.53	High	Yes	1 violation	0.55
**3b**	24	5	1	5	0.06	3.55	High	Yes	Yes 0 violation	0.55
**4a**	20	4	5	2	0	4.53	High	Yes	1 violation	0.55
**4i**	20	4	6	1	0	4.53	High	Yes	1 violation	0.55
**4j**	20	4	5	1	0.07	4.58	High	Yes	1 violation	0.55
**4k**	20	4	5	1	0	5.01	High	Yes	1 violation	0.55
**5**	32	6	3	0	0.48	2.58	High	Yes	Yes 0 violation	0.55

HA: number of heavy atoms; RB: number of rotatable bonds; HBA: number of H-bond acceptors; HBD: number of H-bond donors; FC: fraction Csp3; MLOGP: LogP_o/w_ topological method; GI absorption: gastrointestinal absorption; BBB permeation: blood–brain barrier permeation.

## Data Availability

Data is contained within the article or supplementary data.

## Supplementary Materials

The following supporting information can be downloaded at https://www.mdpi.com/article/10.3390/ph18050608/s1. Computer-aided drug design by the Swiss ADME programs for representative compounds **1**, **2a**–**c**, **3b** and **5**; analytical spectra of series **2** (**2a**–**s**), **3a**,**b**, **4a**–**k** and **5**: mass (MS), high-resolution mass spectroscopy (HRMS), LC purity, proton (1H) NMR, 19F NMR, 2D COSY NMR, 2D NOESY NMR, etc. Figure S1: Computer-aided drug design by Swiss ADME programs: Compounds **1**, **2a**–**c**, **5** and **3b;** Table S1: Summary of ADME data (**1**, **2a-c**, **5**, and **3b**); Figure S2. Analytical spectrums of Series **2** (**2a**–**s**). Figure S3. Analytical spectrums of Series **3**~**5** (**3a**–**b**, **4a**–**k**, **5**).

## Abbreviations

The following abbreviations were used in this manuscript:

ADME	Absorption, Distribution, Metabolism and Excretion
calcd	Calculated
ESI	Electrospray ionization
EtOAc	Ethyl acetate
GBM	Glioblastoma
LCMS	Liquid chromatography/mass spectrometry
*m*/*z*	Mass-to-charge ratios
NOE	Nuclear Overhauser effect
TED	Therapeutic-enhancing drugs
*t*_min_	Time in minutes
TLC	Thin-layer chromatography
TMZ	Temozolomide
t*_R_*	Time resolution
