# Supplementary material for: Structural Activity Relationship Analysis of New Diphenyl PFI-3 Analogues Targeting for the Treatment of Glioblastoma
Source: Pharmaceuticals (Basel). 2025 Apr 23;18(5):608. doi: 10.3390/ph18050608 (PMC12114682; doi:10.3390/ph18050608)

Supporting Information

Structural Activity Relationship Analysis of New Diphenyl PFI-3 Analogues Targeting for the Treatment of Glioblastoma

Dong-Jin Hwang [1], Chuanhe Yang [2], Yinan Wang [2], Hannah Kelso [2], Satyanarayana Pochampally [1], Lawrence M. Pfeffer [2,3,†] and Duane D. Miller [1,3,*,†]

[1] Department of Pharmaceutical Sciences, College of Pharmacy, University of Tennessee Health Science
Center, Memphis, TN 38163, USA; dhwang@uthsc.edu (D.-J.H.); spochamp@uthsc.edu (S.P.)
[2] Department of Pathology and Laboratory Medicine, College of Medicine, University of Tennessee Health Science Center, Memphis, TN 38103, USA; cyang@uthsc.edu (C.Y.); ywang127@uthsc.edu (Y.W.); hanrkels@uthsc.edu (H.K.); lpfeffer@uthsc.edu (L.M.P.)
[3] The Center for Cancer Research, College of Medicine, University of Tennessee Health Science Center,
Memphis, TN 38103, USA
* Correspondence: dmiller@uthsc.edu; Tel.: +1-901-448-6026
† These authors contributed equally to this work.

Table of Contents

1. Figure S1. Computer-aided drug design by Swiss ADME programs: Compounds **1**, **2a-c**, **5** and **3b** S2
2. Table S1. Summary of ADME data (**1**, **2a-c**, **3b**, **4a** and **5**) S8
3. Figure S2. Analytical spectrums of Series **2** (**2a** – **s**) S10
4. Figure S3. Analytical spectrums of Series **3** ~ **5** (**3a** - **b, 4a -k, 5**) S48

Analytical data of compounds was conducted with Mass (MS), High-resolution mass spectroscopy (HRMS), LC purity, Proton (1H) NMR, 19F NMR, 2D COSY NMR, 2D NOESY NMR etc.

Figure S1. Computer-aided drug design by Swiss ADME (Absorption, Distribution, Metabolism, and Excretion) programs

Compound **1**

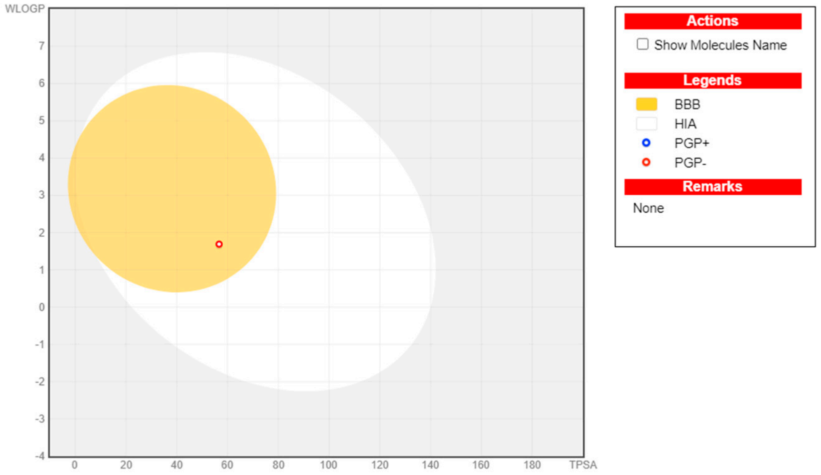

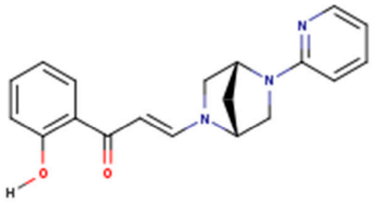

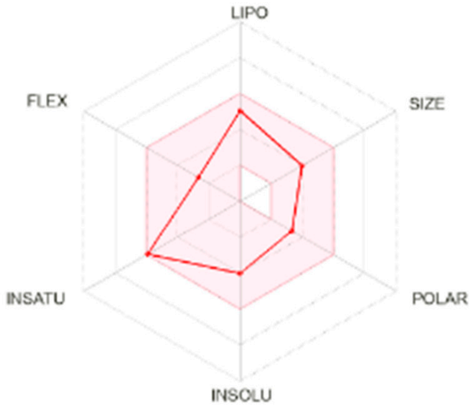

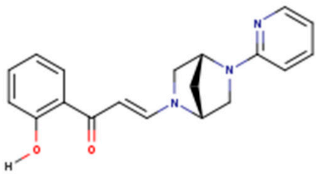

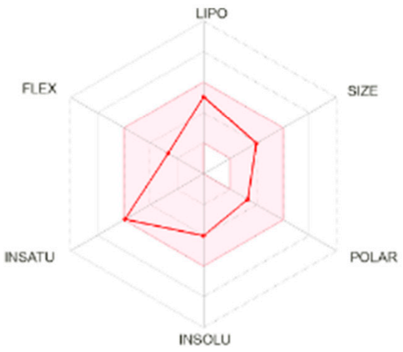

SMILES O=C(c1ccccc1O)/C=C/N1C[C@H]2C[C@@H]1CN2c1ccccn1

Compound **2a**

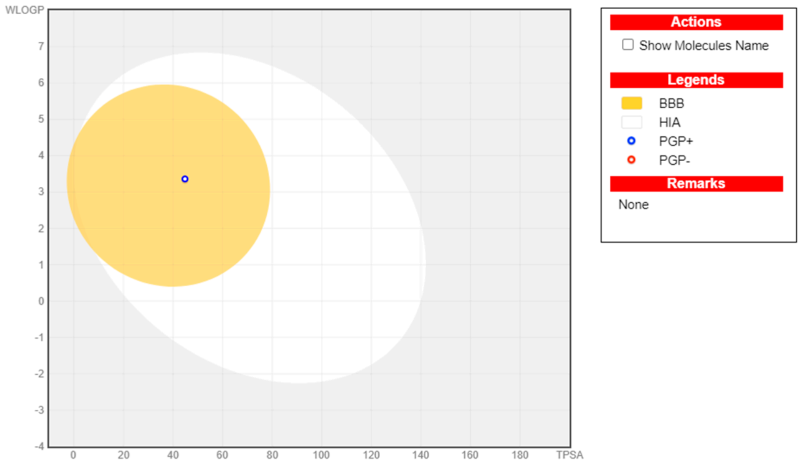

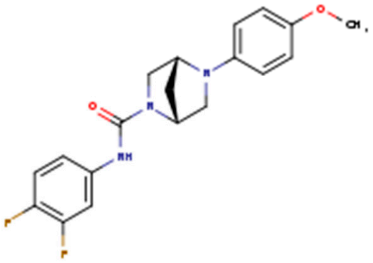

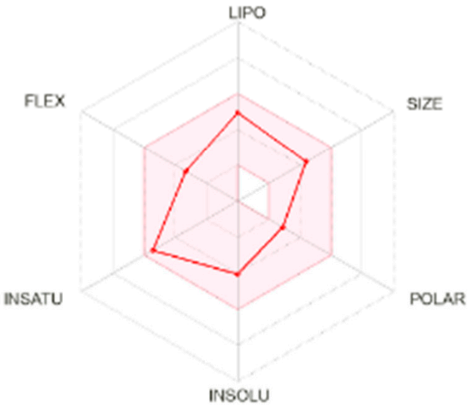

SMILES COc1ccc(cc1)N1C[C@H]2C[C@@H]1CN2C(=O)Nc1ccc(c(c1)F)F

Compound **2b**

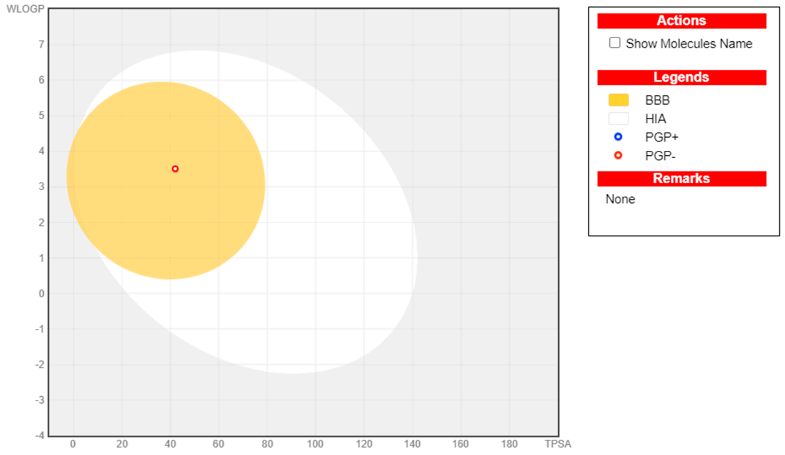

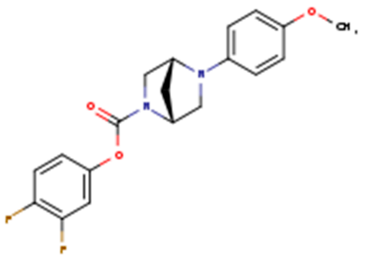

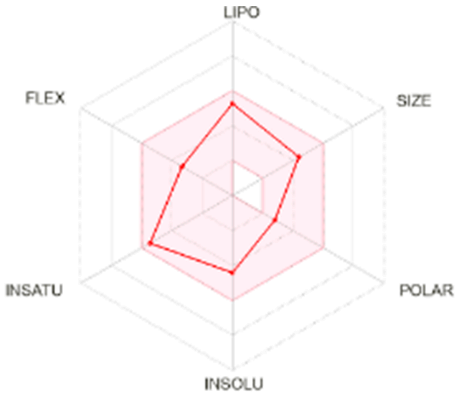

SMILES COc1ccc(cc1)N1C[C@H]2C[C@@H]1CN2C(=O)Oc1ccc(c(c1)F)F

Compound **2c**

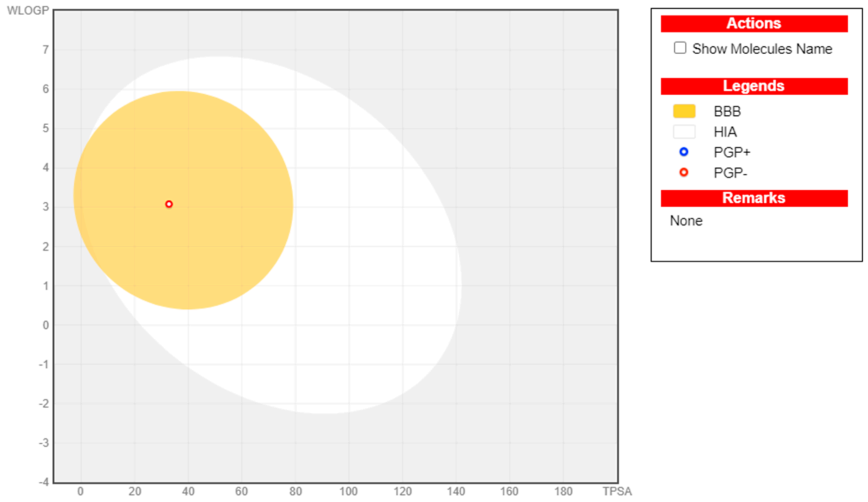

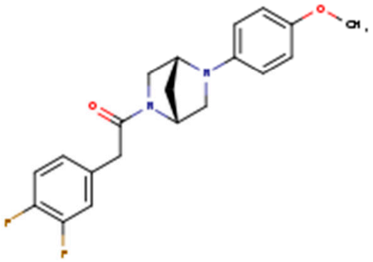

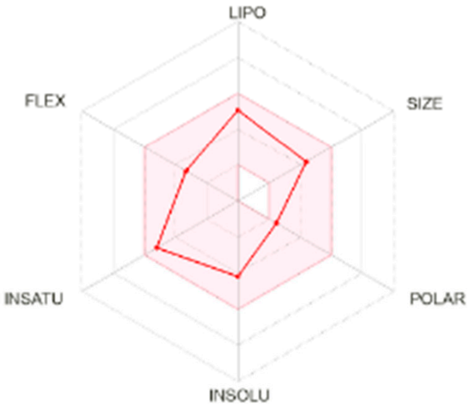

SMILES COc1ccc(cc1)N1C[C@H]2C[C@@H]1CN2C(=O)Cc1ccc(c(c1)F)F

Compound **5**

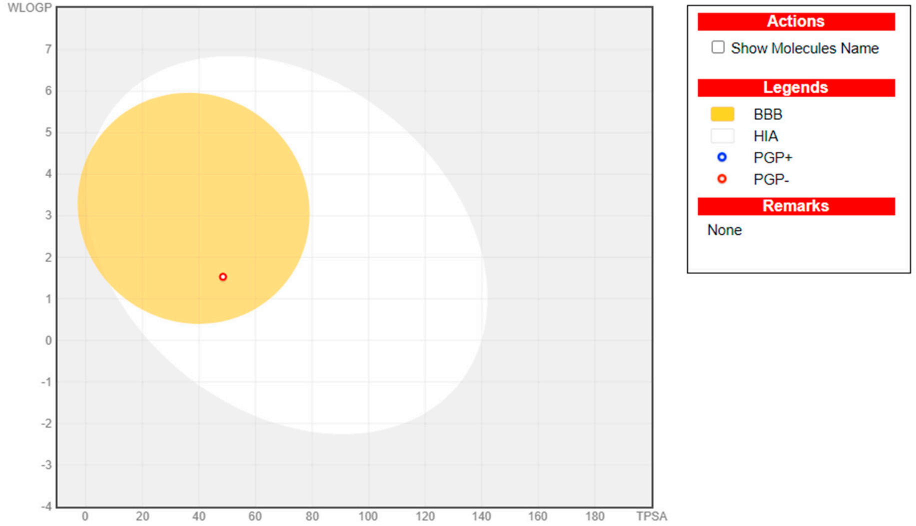

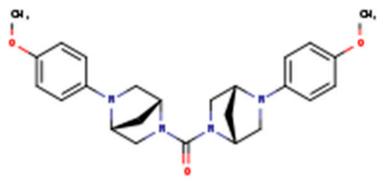

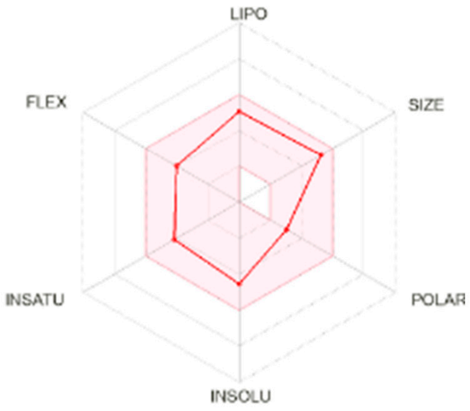

SMILES COc1ccc(cc1)N1C[C@H]2C[C@@H]1CN2C(=O)N1C[C@H]2C[C@@H]1CN2c1ccc(cc1)OC

Compound **3b**

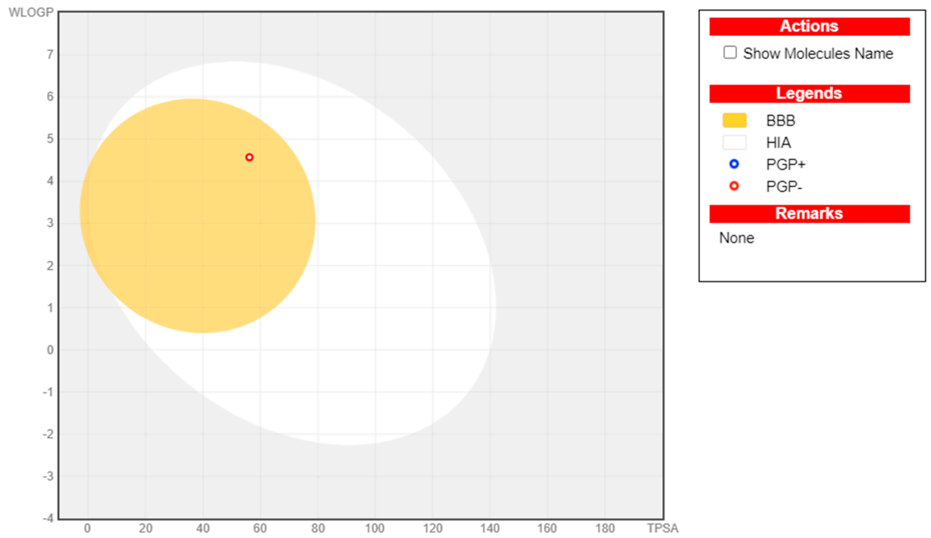

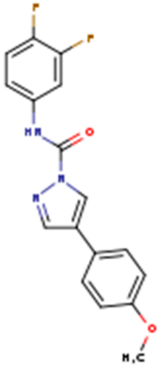

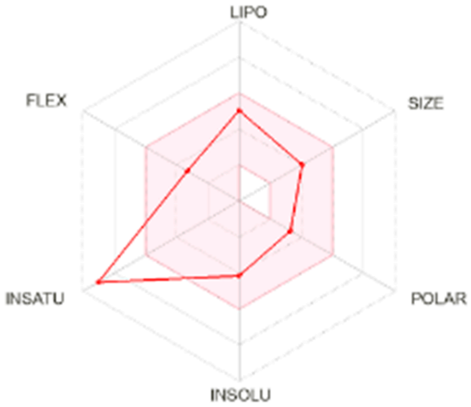

SMILES COc1ccc(cc1)c1cnn(c1)C(=O)Nc1ccc(c(c1)F)F

Table S1. Summary of ADME data (**1**, **2a-c**, **3b**, **4a,** and **5**)

Properties\ID	1	2a	2b	2c	3b	4a	5
**Physicochemical Properties**							
Formula	C19H19N3O2	C19H19F2N3O2	C19H18F2N2O3	C20H20F2N2O2	C17H13F2N3O2	C13H8F4N2O	C25H30N4O3
Molecular weight	321.37 g/mol	359.37 g/mol	360.35 g/mol	358.38 g/mol	329.30 g/mol	284.21 g/mol	434.53 g/mol
Num. heavy atoms	24	26	26	26	24	20	32
Num. aromatic heavy atoms	12	12	12	12	17	12	12
Fraction Csp3	0.26	0.32	0.32	0.35	0.06	0	0.48
Num. Rotatable bonds	4	5	5	5	5	4	6
Num. H-bond acceptors	3	4	5	4	5	5	3
Num. H-bond donors	1	1	0	0	1	2	0
Molar Refractivity	99.26	101.22	98.41	101.3	84.75	65	137.44
TPSA	56.67 Å²	44.81 Å²	42.01 Å²	32.78 Å²	56.15 Å²	41.13 Å²	48.49 Å²
**Lipophilicity**							
LogPo/w(iLOGP)	2.57	3.15	3.45	3.33	3.21	2.48	3.81
LogPo/w(XLOGP3)	3.29	3.14	3.71	3.35	3.3	2.89	3.39
LogPo/w(WLOGP)	1.69	3.36	3.51	3.08	4.57	5.19	1.53
LogPo/w(MLOGP)	1.46	3.38	3.38	3.36	3.55	4.53	2.58
LogPo/w(Silicos-IT Log P)	1.8	2.35	2.67	3.52	2.98	3.48	1.19
Consensus Log Po/w	2.16	3.08	3.35	3.33	3.52	3.71	2.5
**Water Solubility**							
LogS (ESOL)	-4.01	-4.06	-4.42	-4.18	-4.15	-3.6	-4.55
Solubility	3.13e-02 mg/ml ; 9.75e-05 mol/l	3.13e-02 mg/ml ; 9.75e-05 mol/l	1.36e-02 mg/ml ; 3.78e-05 mol/l	2.35e-02 mg/ml ; 6.55e-05 mol/l	2.31e-02 mg/ml ; 7.00e-05 mol/l	7.09e-02 mg/ml ; 2.50e-04 mol/l	1.22e-02 mg/ml ; 2.81e-05 mol/l
Class	Moderately soluble	Moderately soluble	Moderately soluble	Moderately soluble	Moderately soluble	Soluble	Moderately soluble
LogS (Ali)	-4.16	-3.75	-4.28	-3.72	-4.15	-3.41	-4.09
Solubility	2.25e-02 mg/ml ; 6.99e-05 mol/l	2.25e-02 mg/ml ; 6.99e-05 mol/l	1.88e-02 mg/ml ; 5.21e-05 mol/l	6.89e-02 mg/ml ; 1.92e-04 mol/l	2.31e-02 mg/ml ; 7.00e-05 mol/l	1.10e-01 mg/ml ; 3.86e-04 mol/l	3.55e-02 mg/ml ; 8.18e-05 mol/l
Class	Moderately soluble	Soluble	Moderately soluble	Soluble	Moderately soluble	Soluble	Moderately soluble

LogS (Silicos-IT)	-3.58	-5.15	-4.84	-5.5	-6.1	-6.08	-4.19
Solubility	8.53e-02 mg/ml ; 2.65e-04 mol/l	8.53e-02 mg/ml ; 2.65e-04 mol/l	5.26e-03 mg/ml ; 1.46e-05 mol/l	1.13e-03 mg/ml ; 3.16e-06 mol/l	2.59e-04 mg/ml ; 7.87e-07 mol/l	2.37e-04 mg/ml ; 8.34e-07 mol/l	2.78e-02 mg/ml ; 6.40e-05 mol/l
Class	Soluble	Moderately soluble	Moderately soluble	Moderately soluble	Poorly soluble	Poorly soluble	Moderately soluble
**Pharmacokinetics**							
GI absorption	High	High	High	High	High	High	High
BBB permeant	Yes	Yes	Yes	Yes	Yes	Yes	Yes
P-gp substrate	No	Yes	No	No	No	No	No
CYP1A2 inhibitor	Yes	Yes	Yes	No	Yes	No	No
CYP2C19 inhibitor	No	No	No	No	Yes	Yes	Yes
CYP2C9 inhibitor	Yes	Yes	Yes	Yes	Yes	No	No
CYP2D6 inhibitor	Yes	Yes	Yes	Yes	No	Yes	Yes
CYP3A4 inhibitor	No	No	No	Yes	No	No	No
LogKp (skin permeation)	-5.92 cm/s	-5.92 cm/s	-5.86 cm/s	-6.11 cm/s	-5.97 cm/s	-5.98 cm/s	-6.54 cm/s
**Drug likeness**							
Lipinski	Yes:0 violation	Yes:0 violation	Yes:0 violation	Yes:0 violation	Yes:0 violation	Yes; 1 violation: MLOGP>4.15	Yes:0 violation
Ghose	Yes	Yes	Yes	Yes	Yes	Yes	No.1 violation: MR>130
Veber	Yes	Yes	Yes	Yes	Yes	Yes	Yes
Egan	Yes	Yes	Yes	Yes	Yes	Yes	Yes
Muegge	Yes	Yes	Yes	Yes	Yes	Yes	Yes
Bioavailability Score	0.55	0.55	0.55	0.55	0.55	0.55	0.55
**Medicinal Chemistry**							
PAINS	0 alert	0 alert	1alert:anil_di_alk_C	1alert:anil_di_alk_C	0 alert	0 alert	1alert:anil_di_alk_C
Brenk	1 alert:michael_acceptor_1	1 alert:anil_di_alk_C	0 alert	0alert	0 alert	0 alert	0 alert
Lead likeness	Yes	No:1 violation: MW>350	No:2 violations: MW>350	No:1 violation: MW>350	Yes	Yes	No:1 violation: MW>350
Synthetic accessibility	4.33	4.11	4.24	3.91	2.64	1.99	5.16

Figure S2. Analytical spectrums of Series 2 (**2a** ~ **2s**)

(1*R*,4*R*)-*N*-(3,4-Difluorophenyl)-5-(4-methoxyphenyl)-2,5-diazabicyclo[2.2.1]heptane-2-carboxamide (2a)

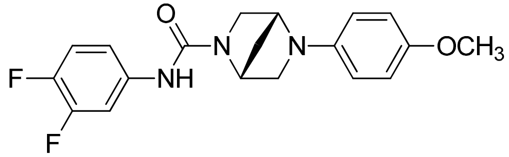

^{1}H NMR (DMSO-d6)

Calculated HRMS: 360.1524 [M + H] $^{+}$

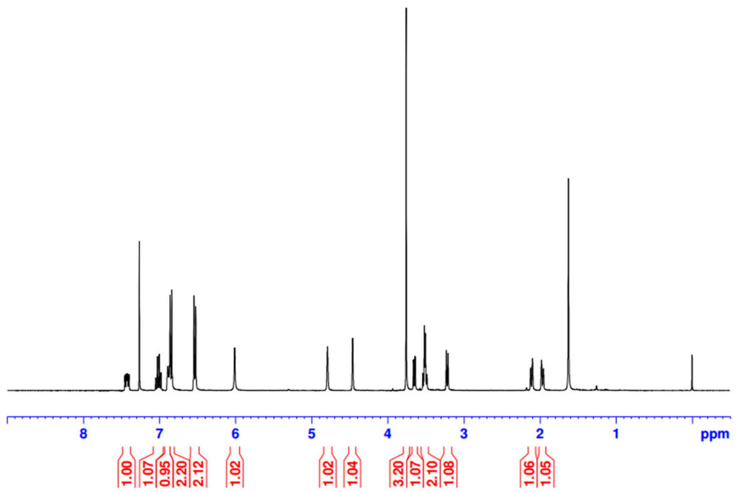

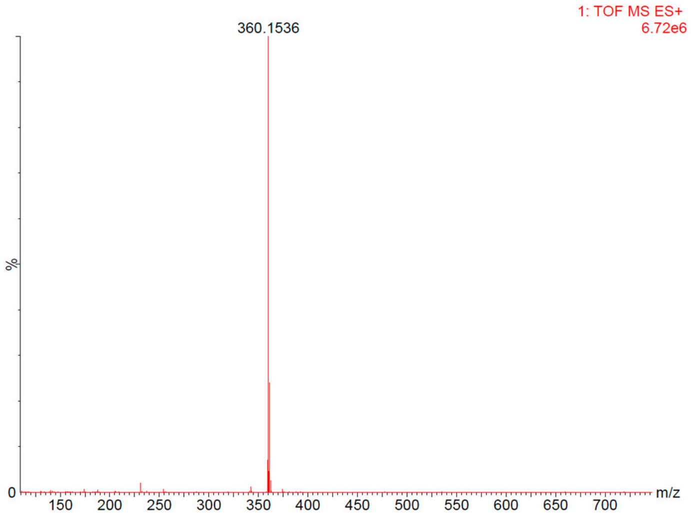

(1*R*,4*R*)-*N*-(3,4-Difluorophenyl)-5-(4-methoxyphenyl)-2,5-diazabicyclo[2.2.1]heptane-2-carboxamide (2a)

2D NOESY NMR (DMSO-d6)

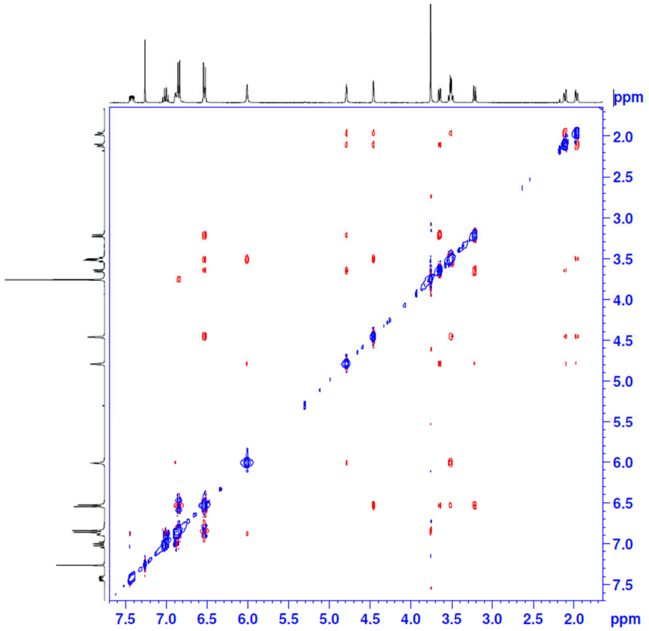

Purity (LC, t_R 3.35 min) 99.10%

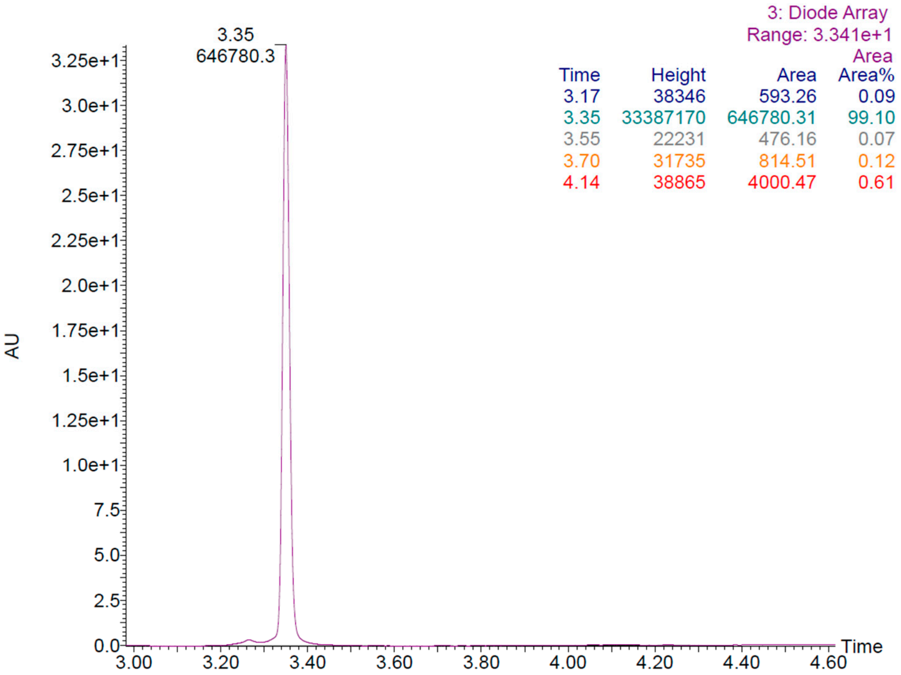

(1*R*,4*R*)-3,4-Difluorophenyl 5-(4-methoxyphenyl)-2,5-diazabicyclo[2.2.1]heptane-2-carboxylate (2b)

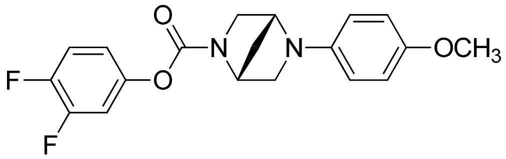

^{1}H NMR (DMSO-d6)

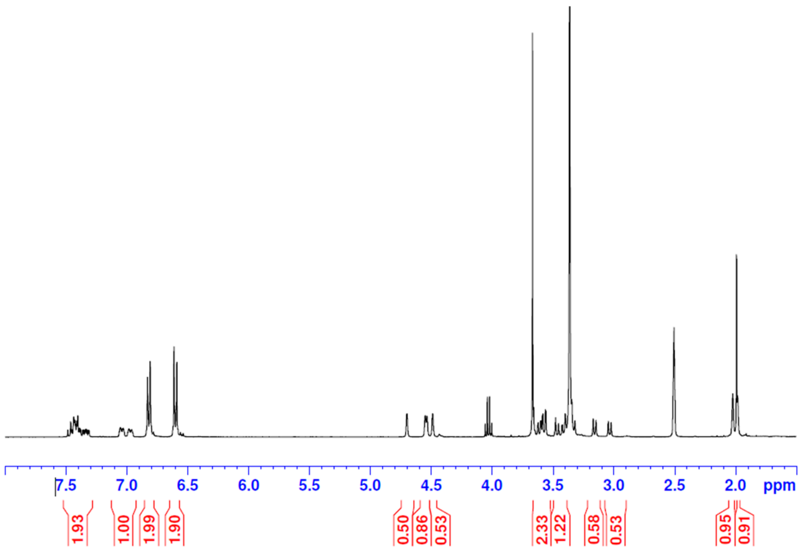

Calculated HRMS: 361.1364 [M + H] $^{+}$

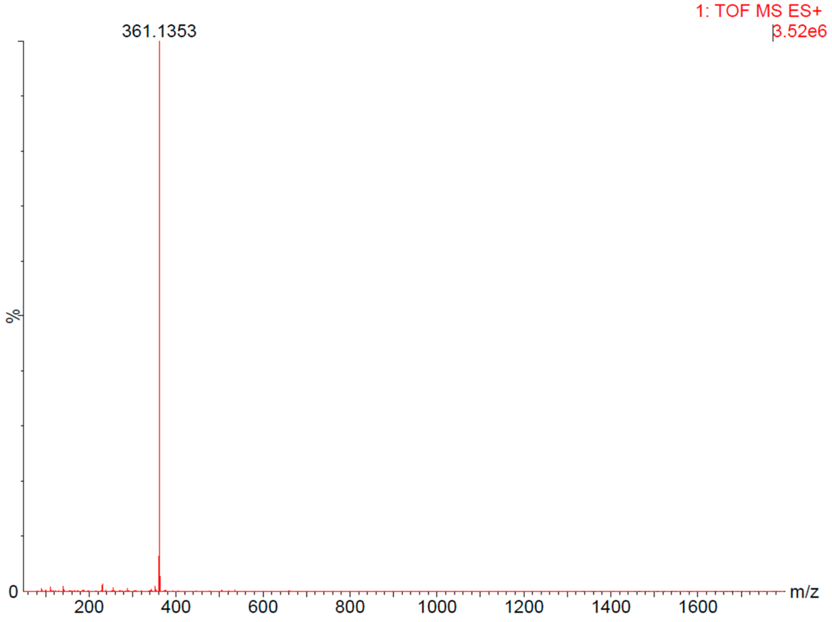

(1*R*,4*R*)-3,4-Difluorophenyl 5-(4-methoxyphenyl)-2,5-diazabicyclo[2.2.1]heptane-2-carboxylate (2b)

^{19}F NMR (DMSO-d6)

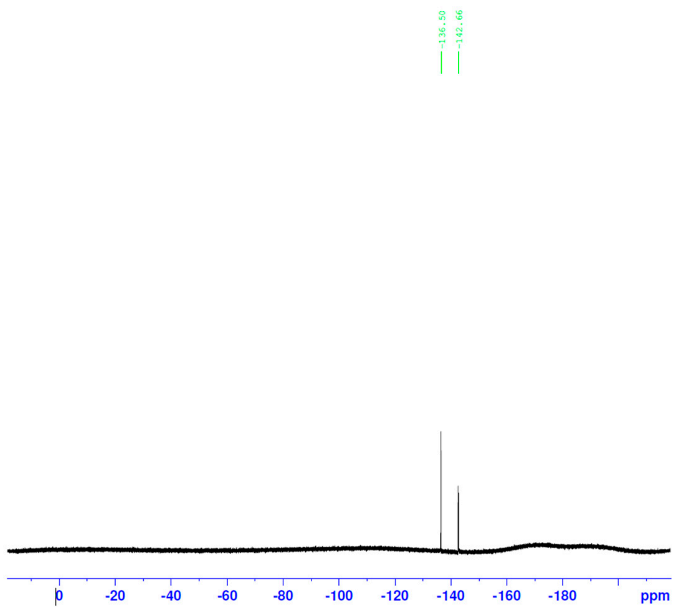

2D NOESY NMR (DMSO-d6)

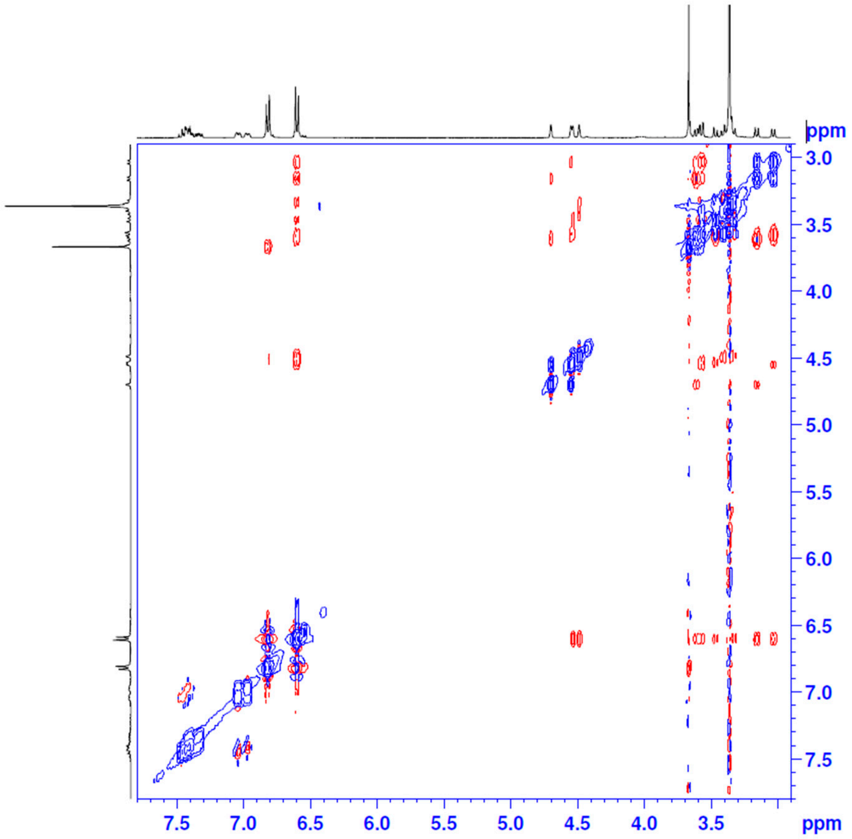

2-(3,4-Difluorophenyl)-1-((1*R*,4*R*)-5-(4-methoxyphenyl)-2,5-diazabicyclo[2.2.1]heptan-2-yl)ethenone (2c)

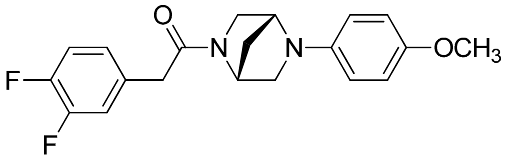

1H NMR (DMSO-d6)

Calculated HRMS 359.1571 [M + H] $^{+}$

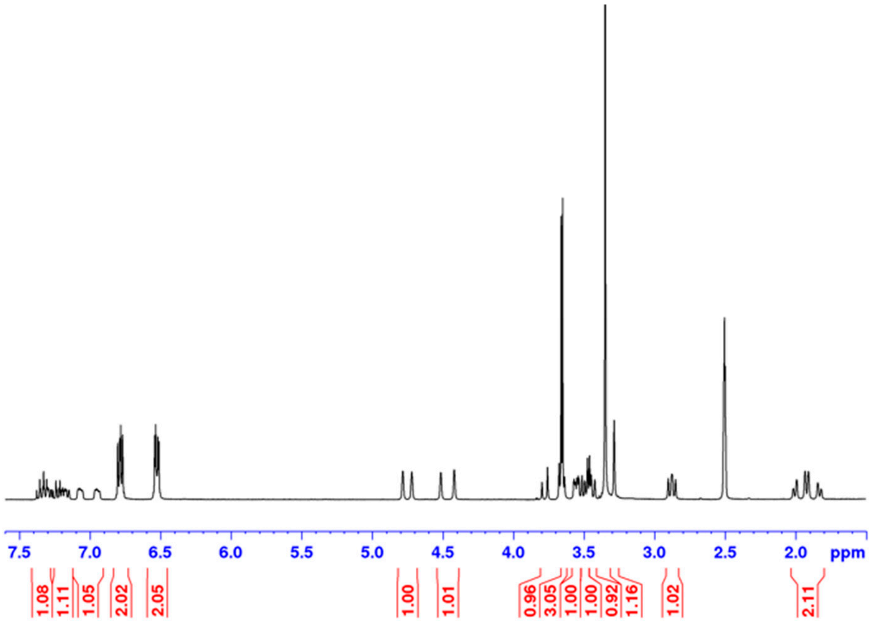

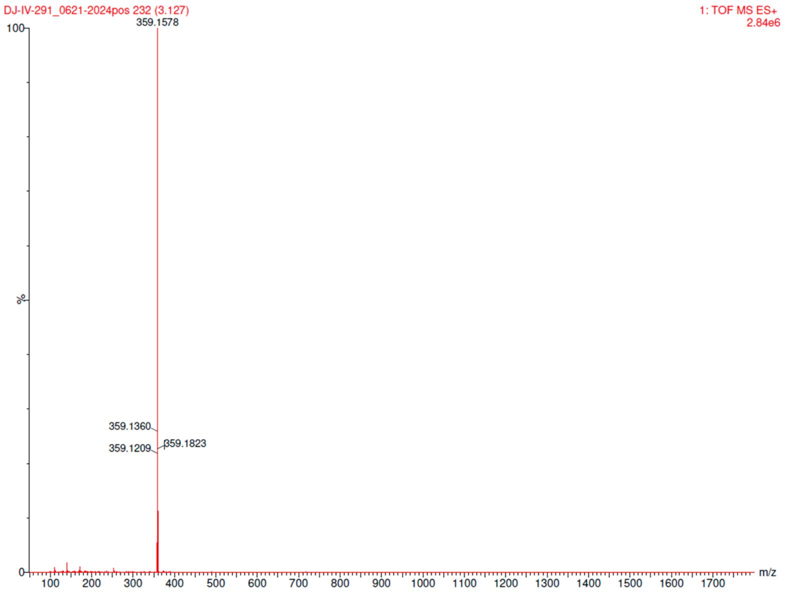

2-(3,4-Difluorophenyl)-1-((1*R*,4*R*)-5-(4-methoxyphenyl)-2,5-diazabicyclo[2.2.1]heptan-2-yl)ethenone (2c)

2D NOESY NMR (DMSO-d6)

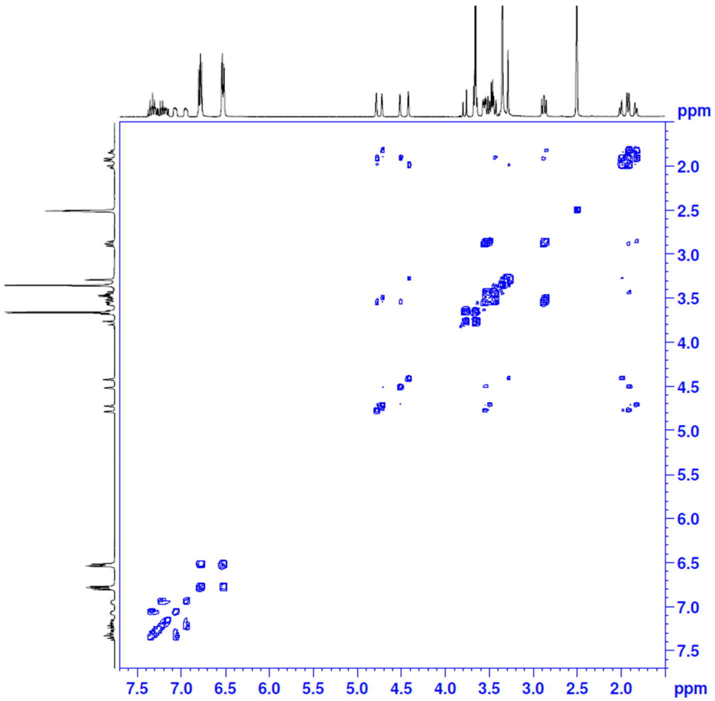

^{19}F NMR (DMSO-d6)

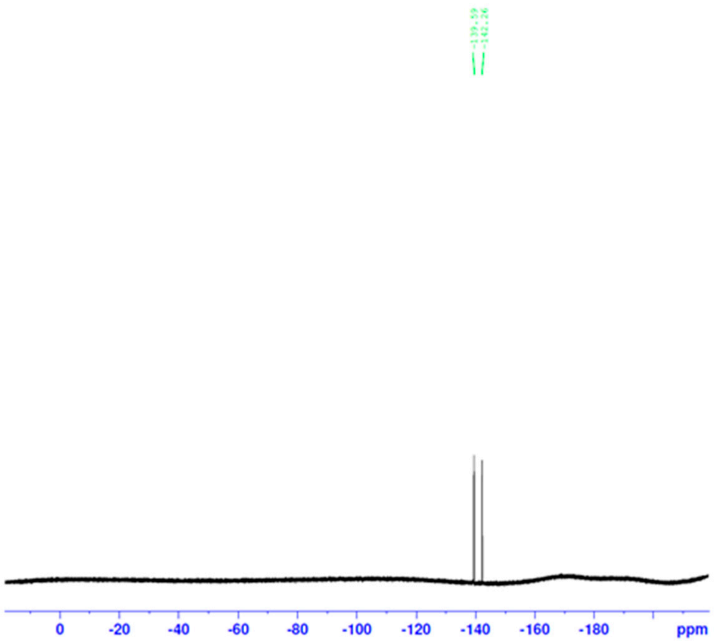

(1*R*,4*R*)-*S*-(3,4-Difluorophenyl) 5-(4-methoxyphenyl)-2,5-diazabicyclo[2.2.1]heptane-2-carbothioate (2d)

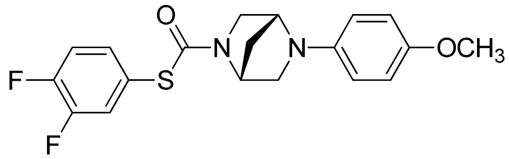

1H NMR (DMSO-d6)

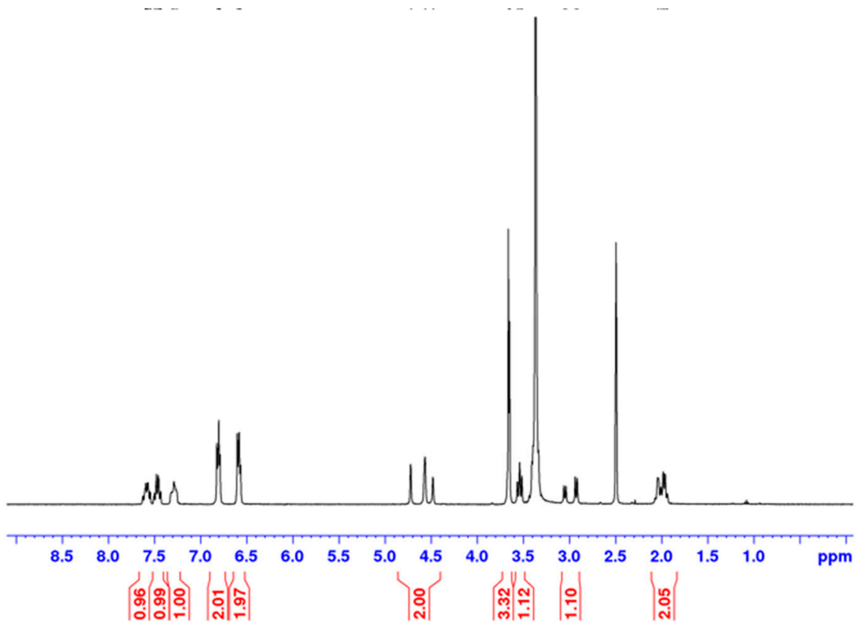

Purity (LC, t_R 3.54 min): 99.67%

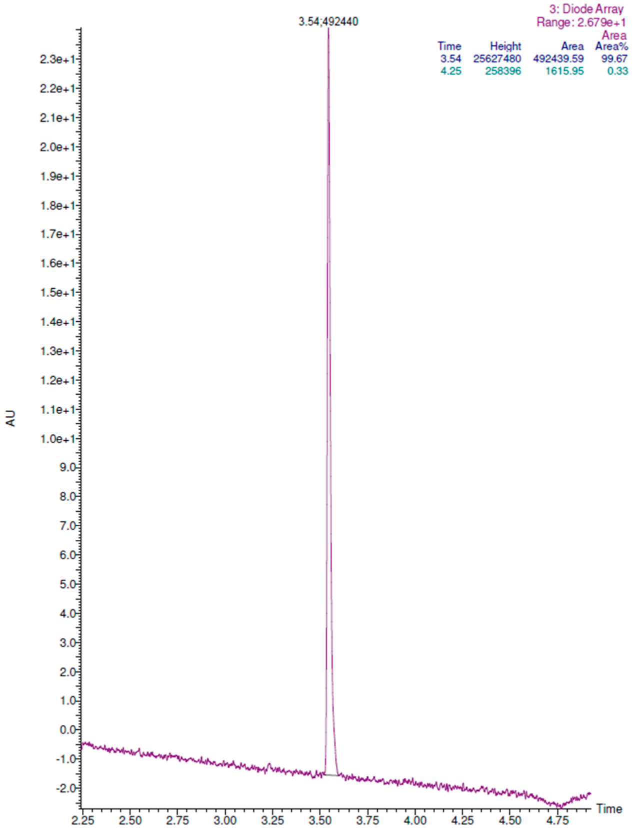

(1*R*,4*R*)-*S*-(3,4-Difluorophenyl) 5-(4-methoxyphenyl)-2,5-diazabicyclo[2.2.1]heptane-2-carbothioate (2d)

2D COSY NMR (DMSO-d6)

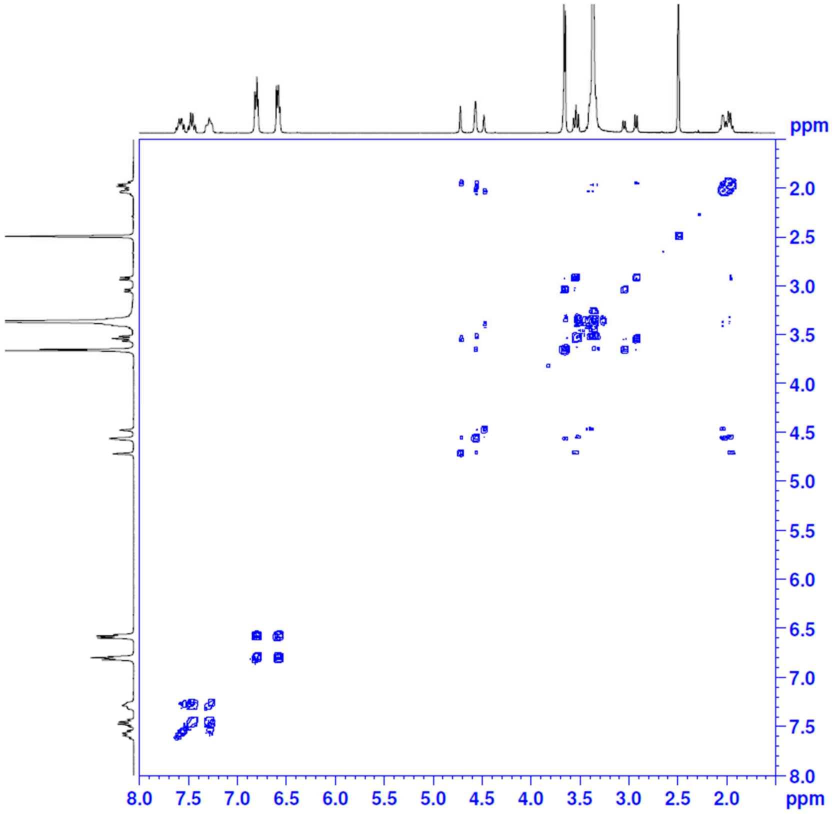

2D NOESY NMR (DMSO-d6)

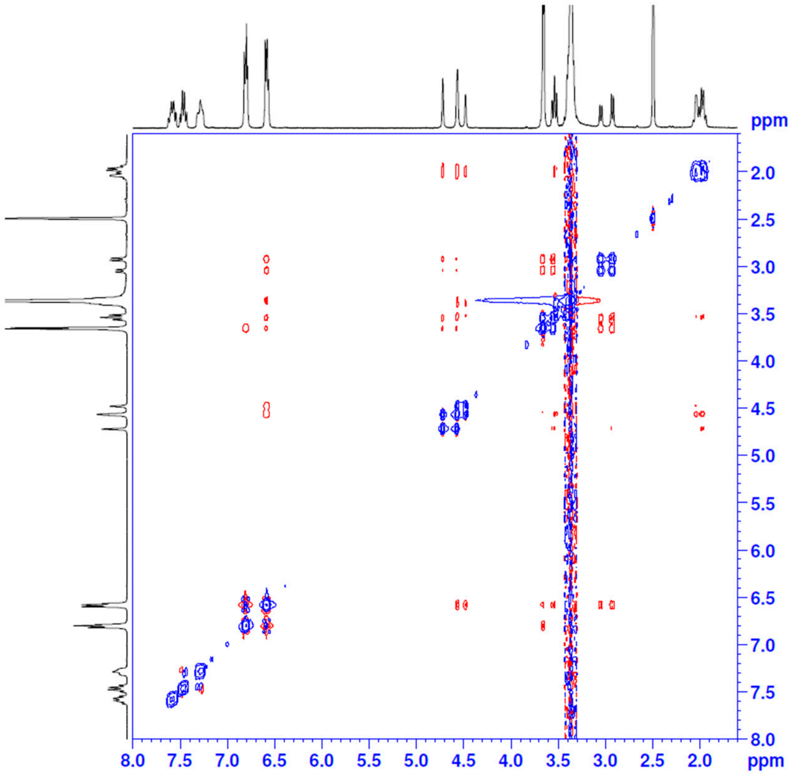

(1*R*,4*R*)-5-(3,4-Dimethoxyphenyl)-N-(4-fluorophenyl)-2,5-diazabicyclo[2.2.1]heptane-2-carboxamide (2e)

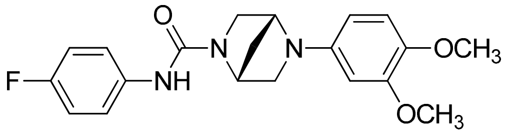

1H NMR (DMSO-d6)

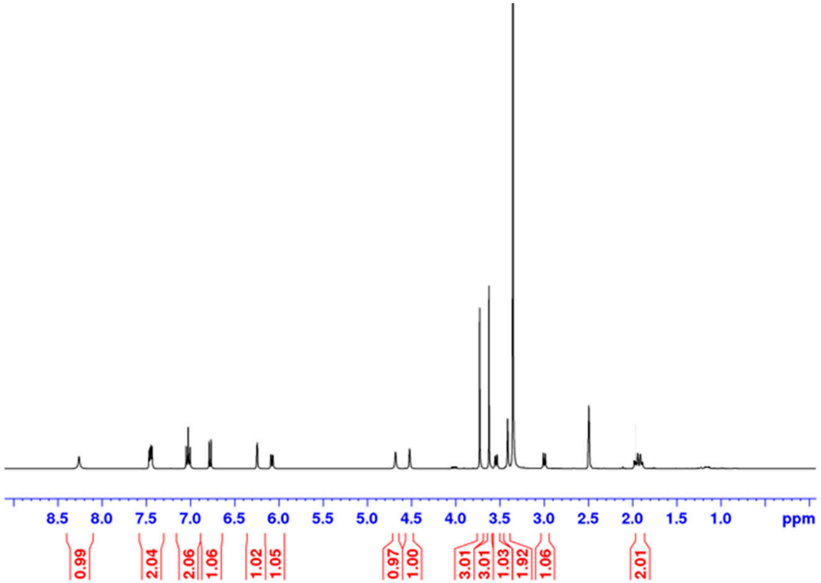

Calculated HRMS 370.1567 [M + H] $^+$

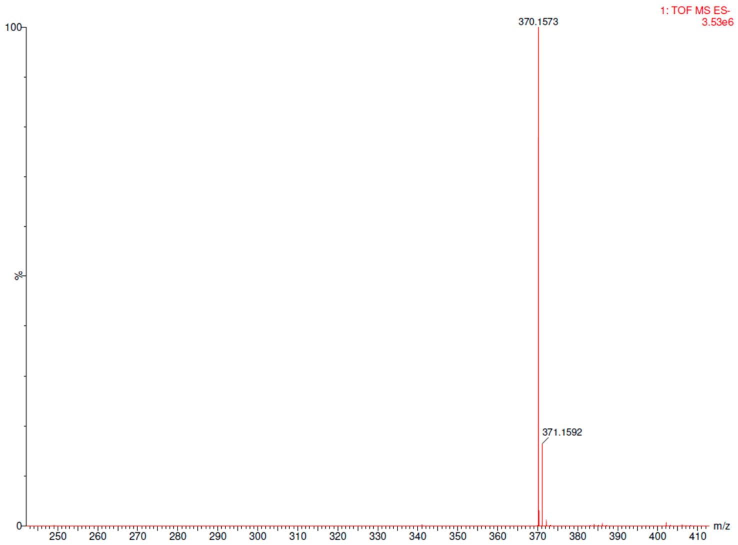

(1*R*,4*R*)-5-(3,4-Dimethoxyphenyl)-N-(4-fluorophenyl)-2,5-diazabicyclo[2.2.1]heptane-2-carboxamide (2e)

Purity: (LC, t_{min} = 3.08) 99.46%

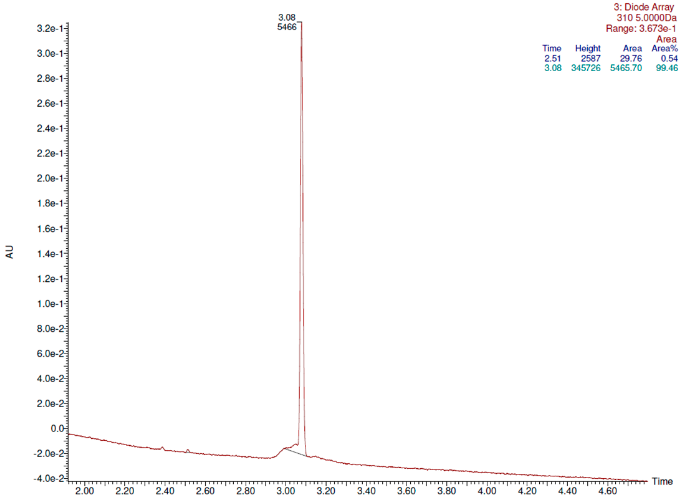

^{19}F NMR (DMSO-d6)

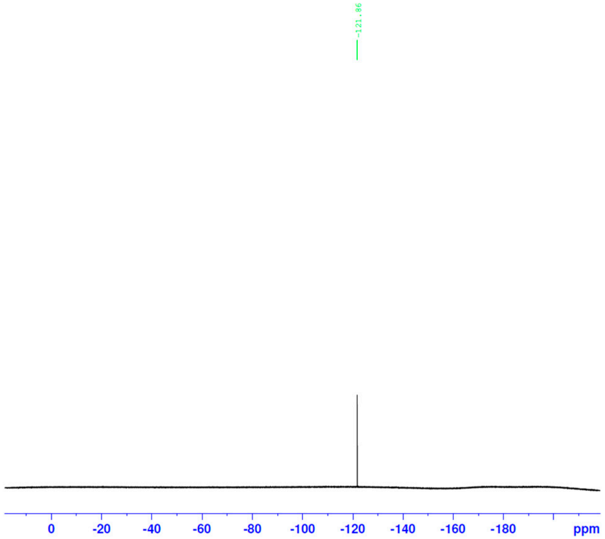

(1*R*,4*R*)-*N*-(3,4-Difluorophenyl)-5-(3,4-dimethoxyphenyl)-2,5-diazabicyclo[2.2.1]heptane-2-carboxamide (2f)

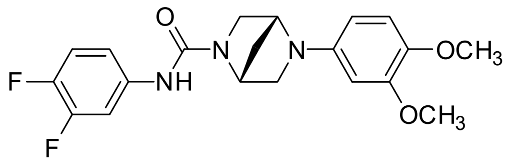

1H NMR (DMSO-d6)

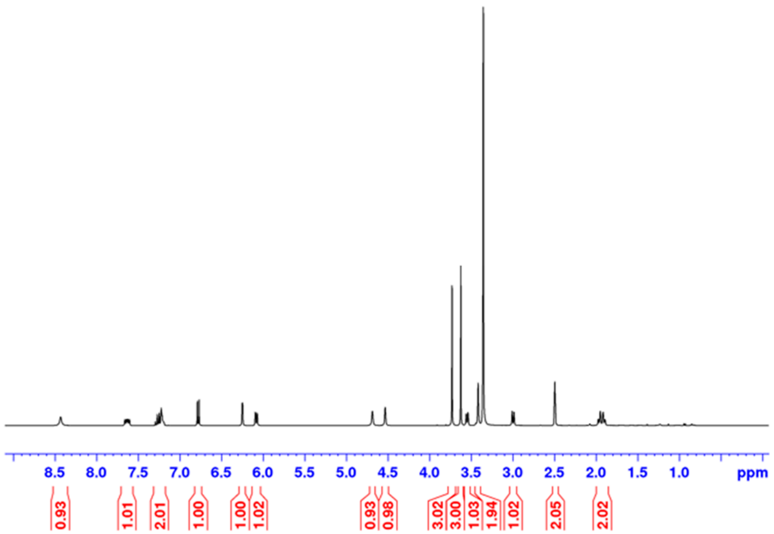

Calculated HRMS 388.1473 [M - H] -

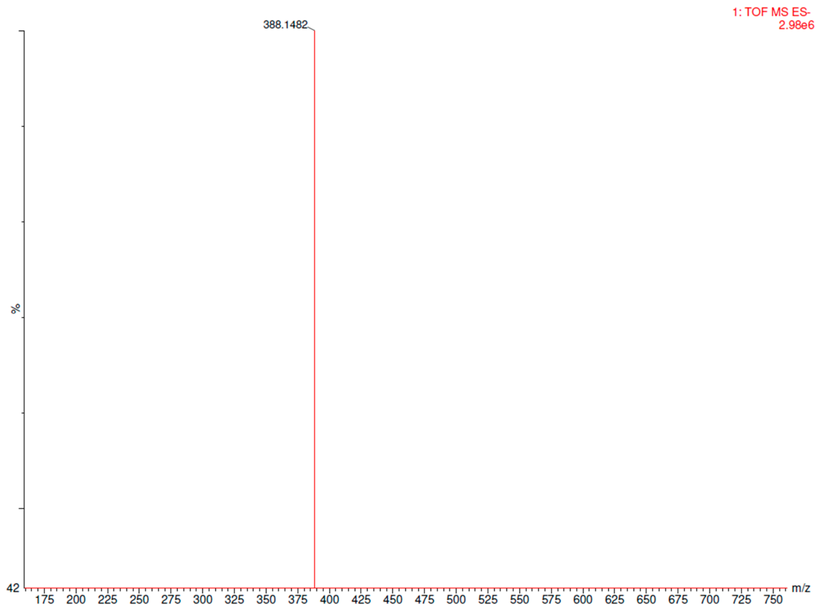

(1*R*,4*R*)-*N*-(3,4-Difluorophenyl)-5-(3,4-dimethoxyphenyl)-2,5-diazabicyclo[2.2.1]heptane-2-carboxamide (2f)

^{19}F NMR (DMSO-d6)

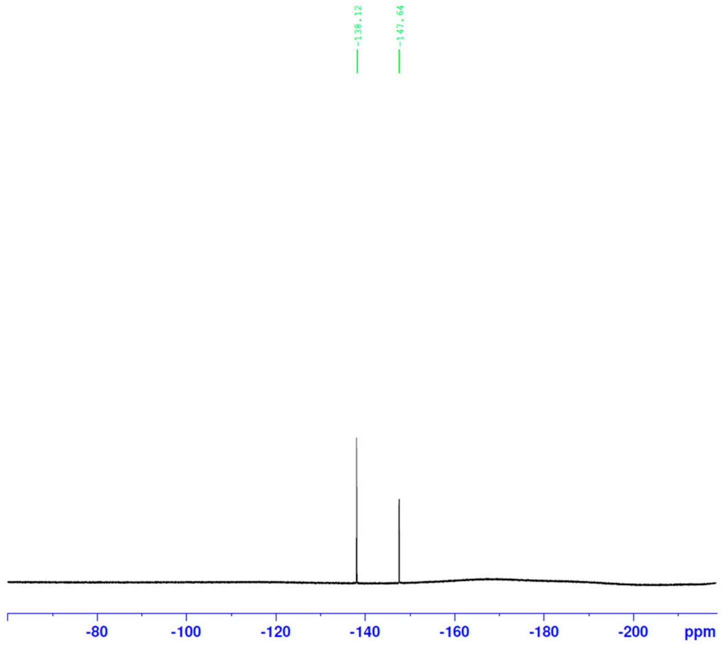

Purity: (LC, t_{min} = 3.21) 99.27%

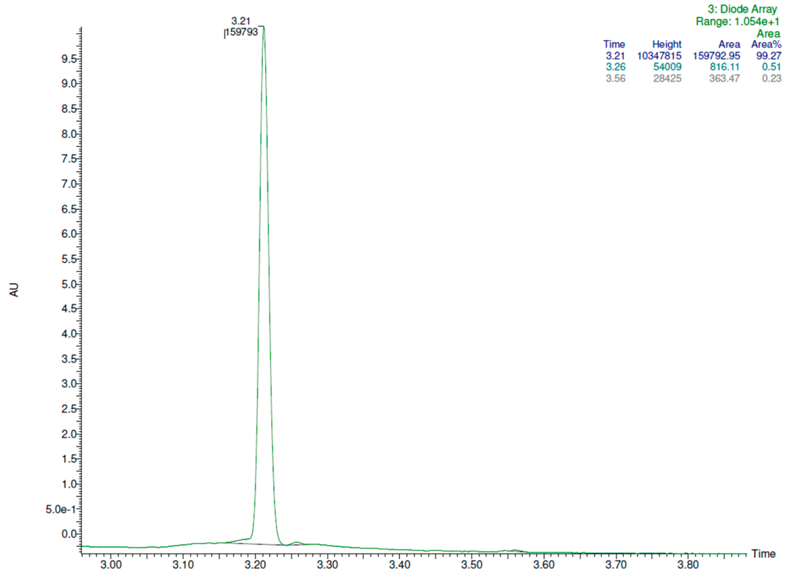

Time	Height	Area	Area%
3.21	10347815	159792.95	99.27
3.26	54009	816.11	0.51
3.56	28425	363.47	0.23

(1*R*,4*R*)-*N*-(3,4-Difluorophenyl)-5-(pyridin-2-yl)-2,5-diazabicyclo[2.2.1]heptane-2-carboxamide (2g)

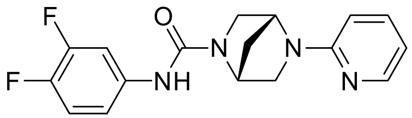

1H NMR (DMSO-d6)

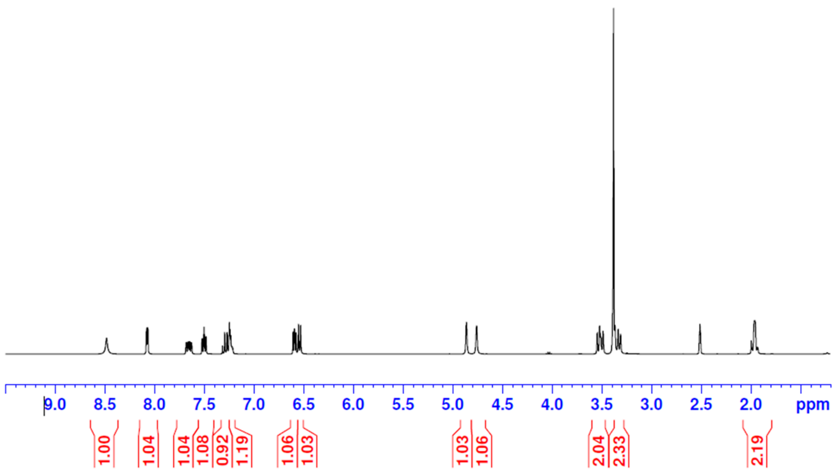

Calculated HRMS [M + H] $^{+}$ 331.1370

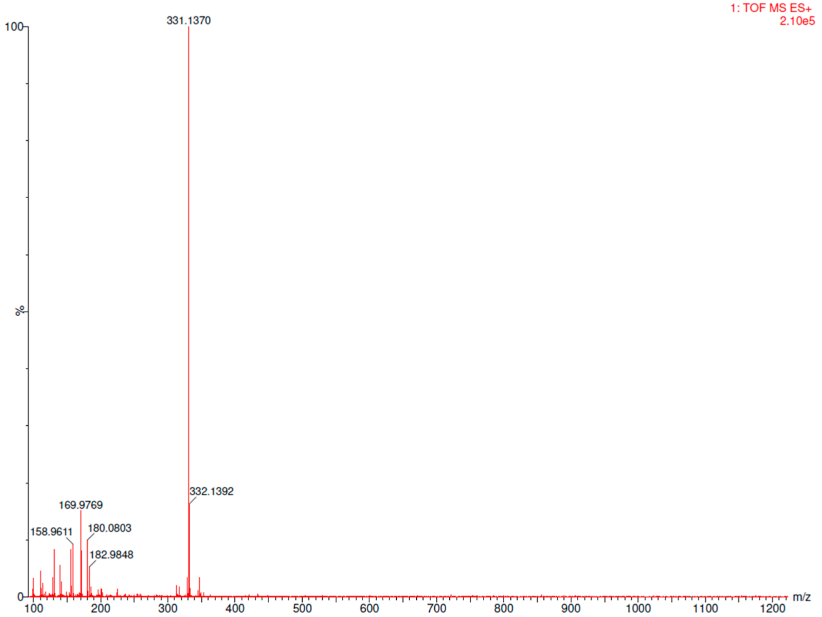

(1*R*,4*R*)-*N*-(3,4-Difluorophenyl)-5-(pyridin-2-yl)-2,5-diazabicyclo[2.2.1]heptane-2-carboxamide (2g)

2D NOESY NMR (DMSO-d6)

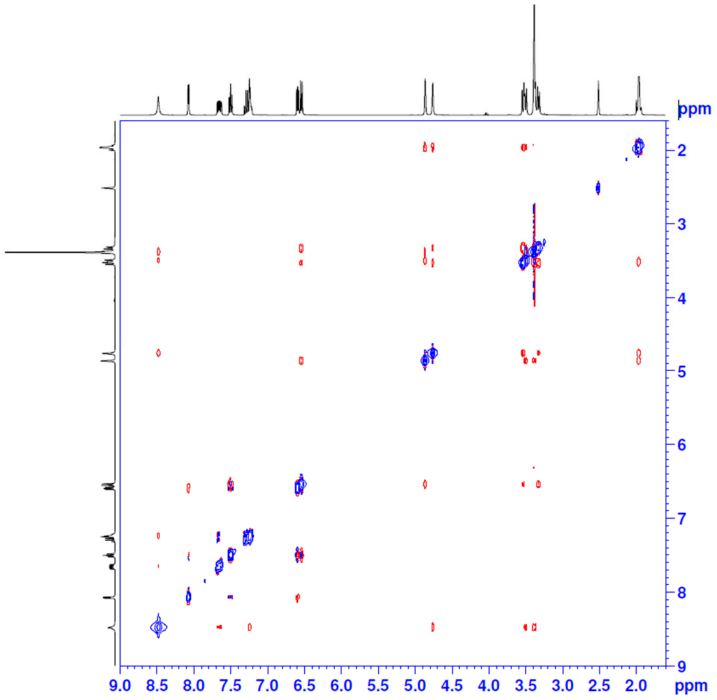

^{19}F (DMSO-d6)

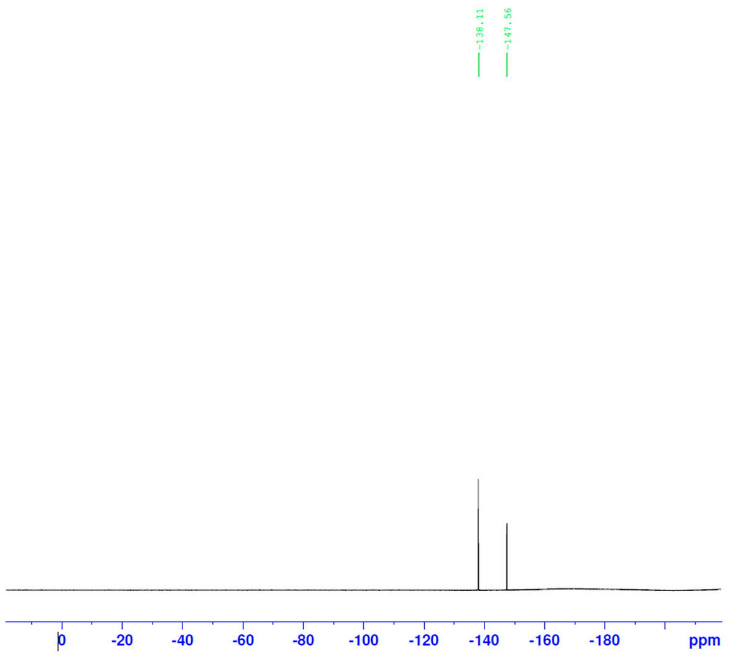

(1*R*,4*R*)-*N*-(7-Chlorobenzo[c][1,2,5]oxadiazol-4-yl)-5-(4-methoxyphenyl)-2,5-diazabicyclo[2.2.1]heptane-2-carboxamide (2h)

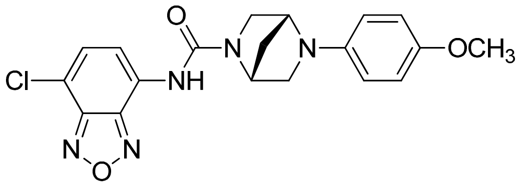

Calculated HRMS [M + H] + 400.1176.

1H NMR (DMSO-d6)

Calculated HRMS [M – H] - 398.1020.

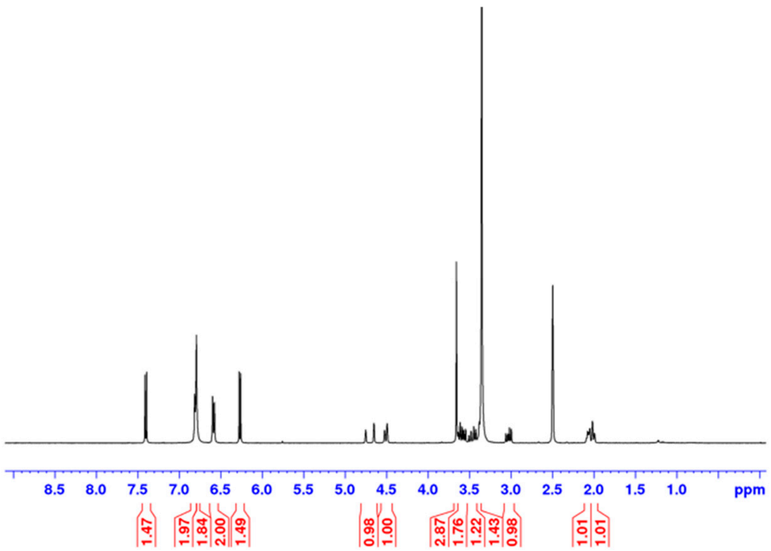

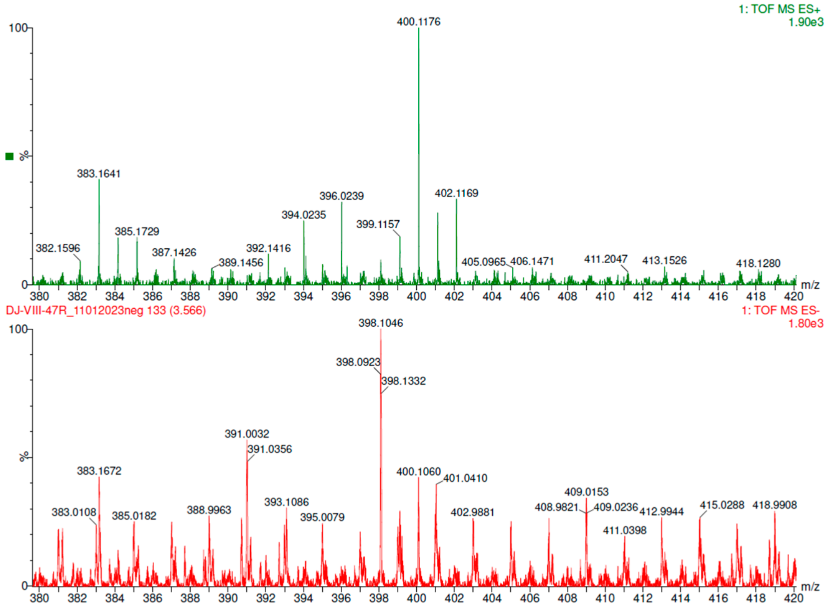

(1*R*,4*R*)-*N*-(7-Chlorobenzo[c][1,2,5]oxadiazol-4-yl)-5-(4-methoxyphenyl)-2,5-diazabicyclo[2.2.1]heptane-2-carboxamide (2h)

2D COSY NMR (DMSO-d6)

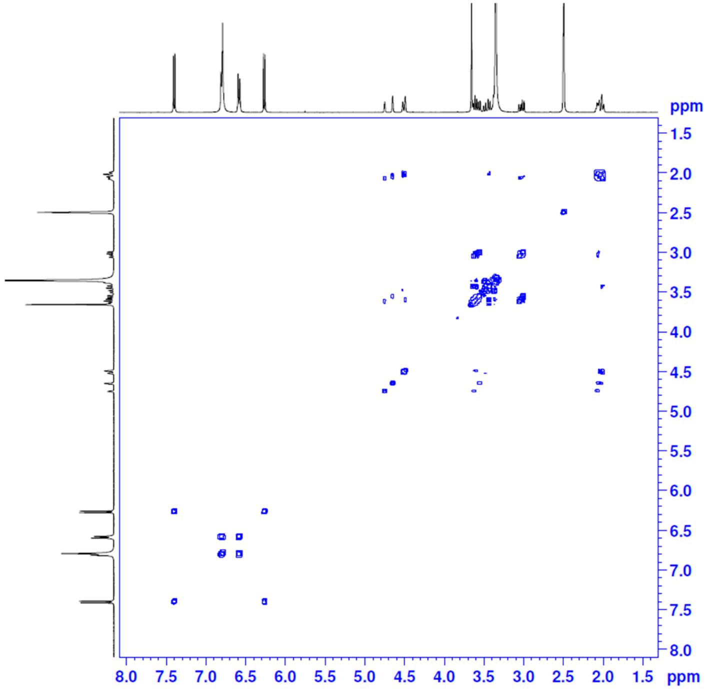

2D NOESY NMR (DMSO-d6)

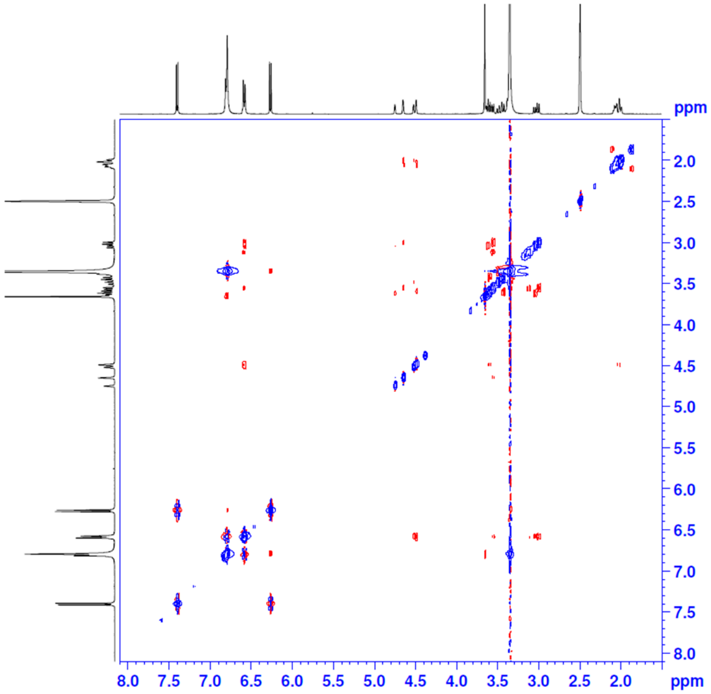

(1*R*,4*R*)-Phenyl 5-(4-methoxyphenyl)-2,5-diazabicyclo[2.2.1]heptane-2-carboxylate (2i)

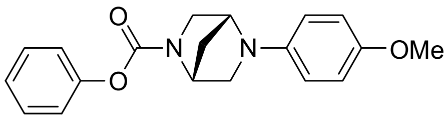

Calculated HRMS $[M + H]^+$ 325.1552

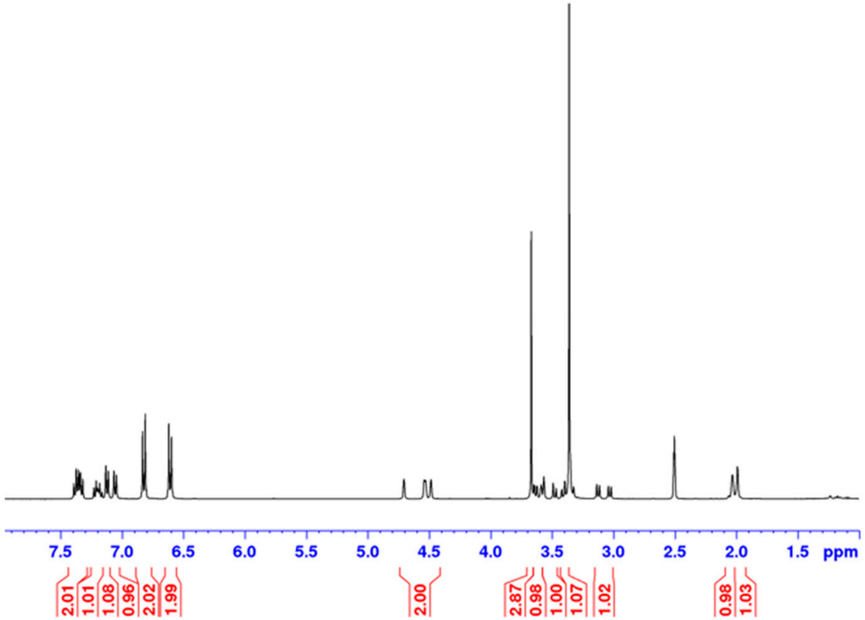

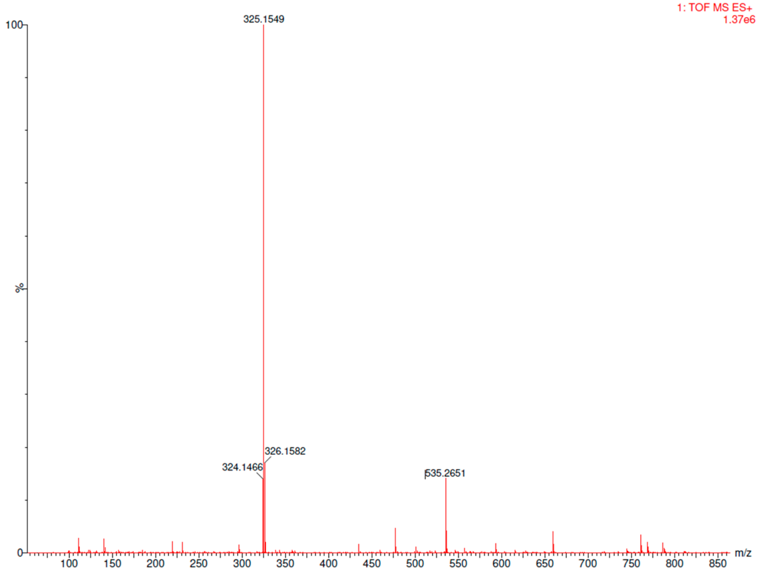

(1*R*,4*R*)-Phenyl 5-(4-methoxyphenyl)-2,5-diazabicyclo[2.2.1]heptane-2-carboxylate (2i)

2D COSY NMR (DMSO-d6)

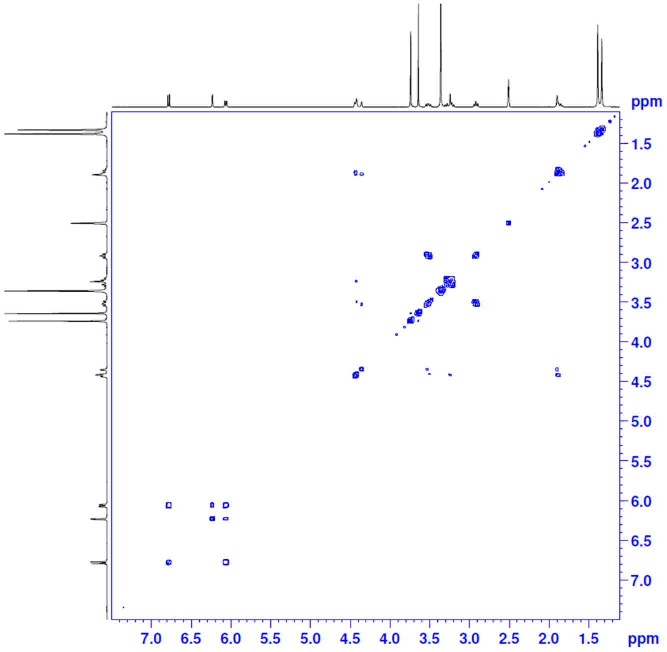

2D NOESY NMR (DMSO-d6)

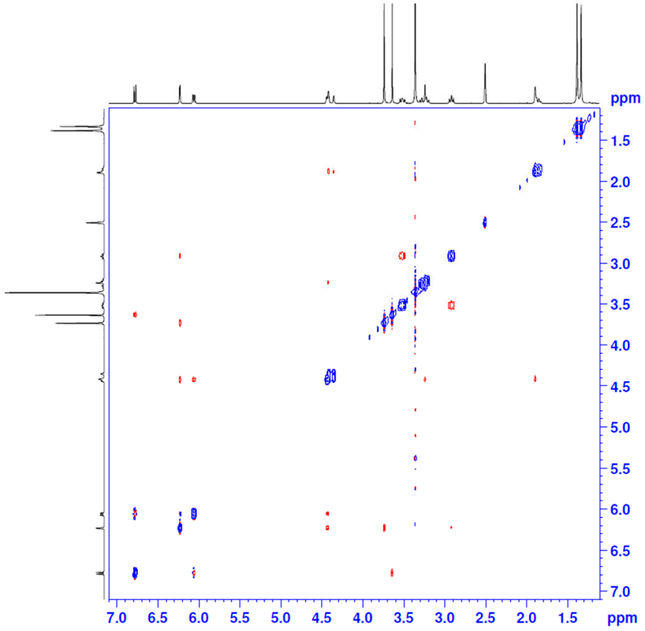

(1*R*,4*R*)-5-(4-Methoxyphenyl)-N-phenyl-2,5-diazabicyclo[2.2.1]heptane-2-carboxamide (2j)

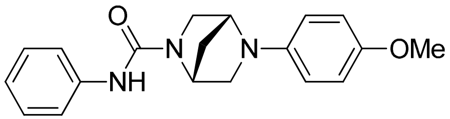

1H NMR (DMSO-d6)

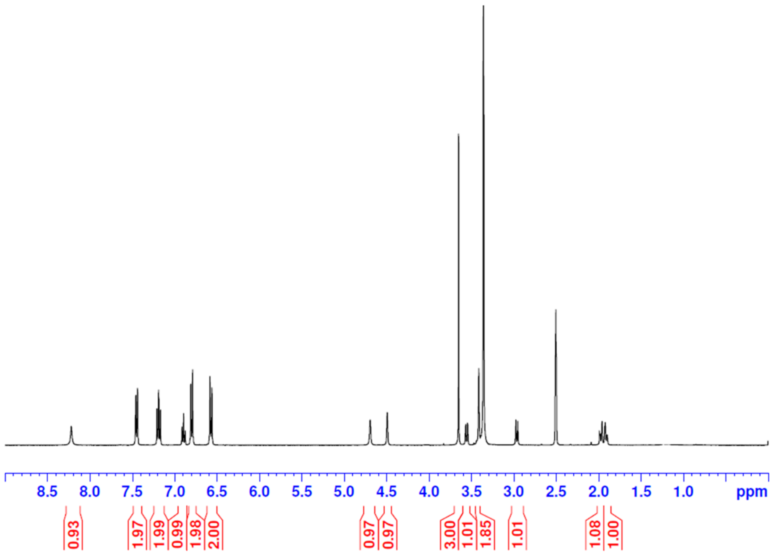

Calculated HRMS [M + H] + 324.1712

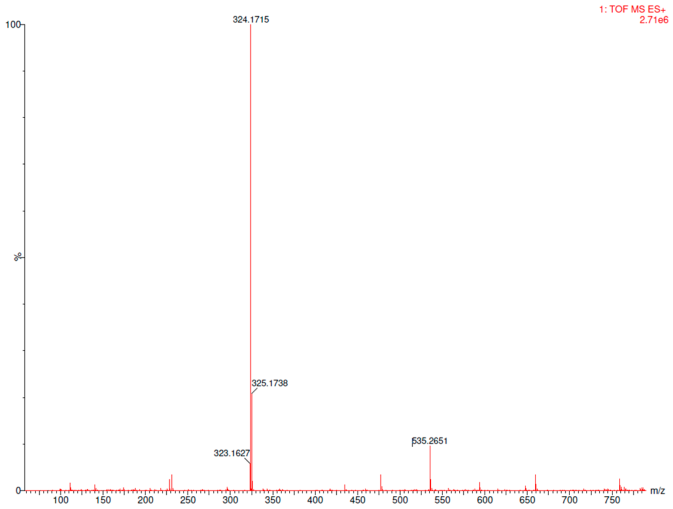

(1*R*,4*R*)-5-(4-Methoxyphenyl)-N-phenyl-2,5-diazabicyclo[2.2.1]heptane-2-carboxamide (2j)

Purity: (LC, t_{min} = 2.92) 98.59%

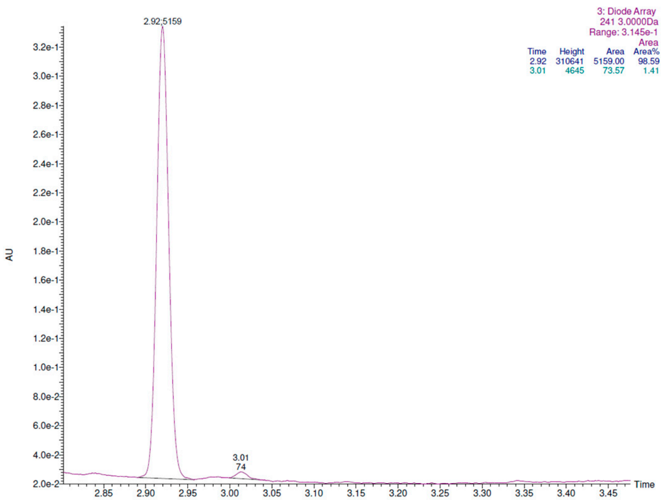

2D COSY NMR (DMSO-d6)

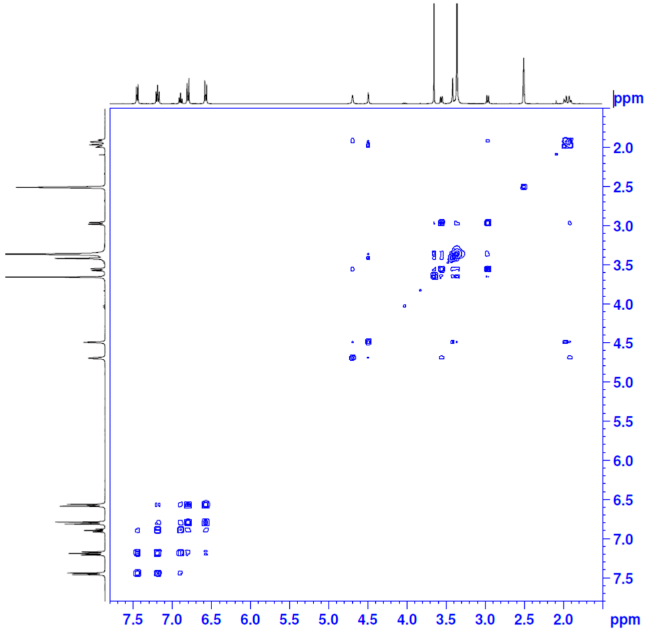

(1*R*,4*R*)-*N*-(4-Fluorophenyl)-5-(4-methoxyphenyl)-2,5-diazabicyclo[2.2.1]heptane-2-carboxamide (2k)

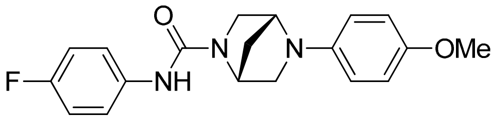

1H NMR (DMSO-d6)

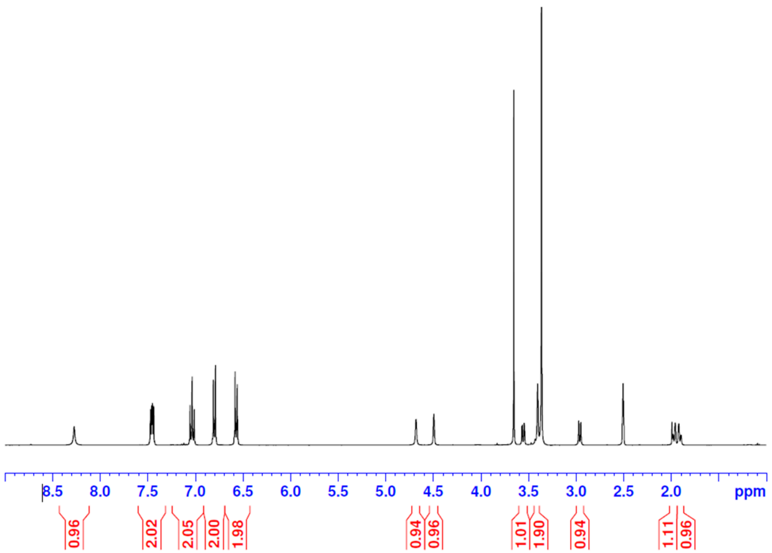

Calculated HRMS [M + H] + 342.1618

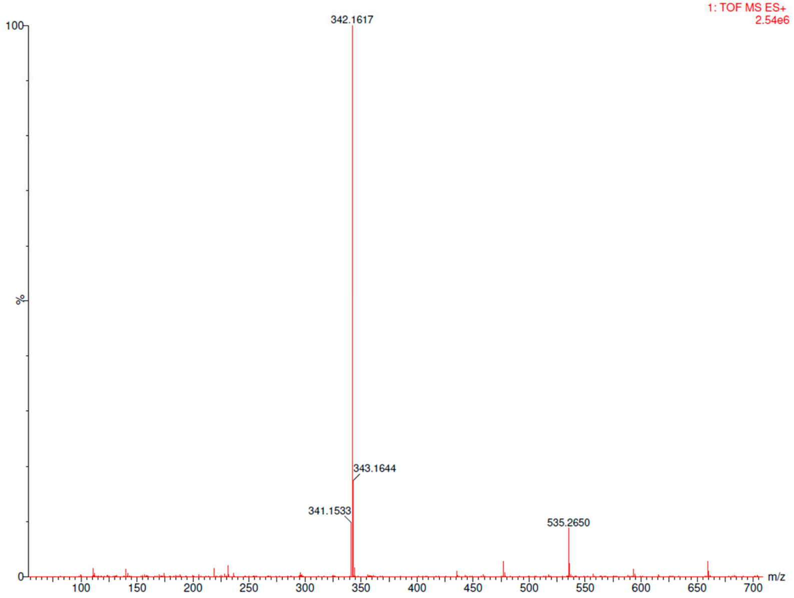

(1*R*,4*R*)-*N*-(4-Fluorophenyl)-5-(4-methoxyphenyl)-2,5-diazabicyclo[2.2.1]heptane-2-carboxamide (2k)

19F NMR (DMSO-d6)

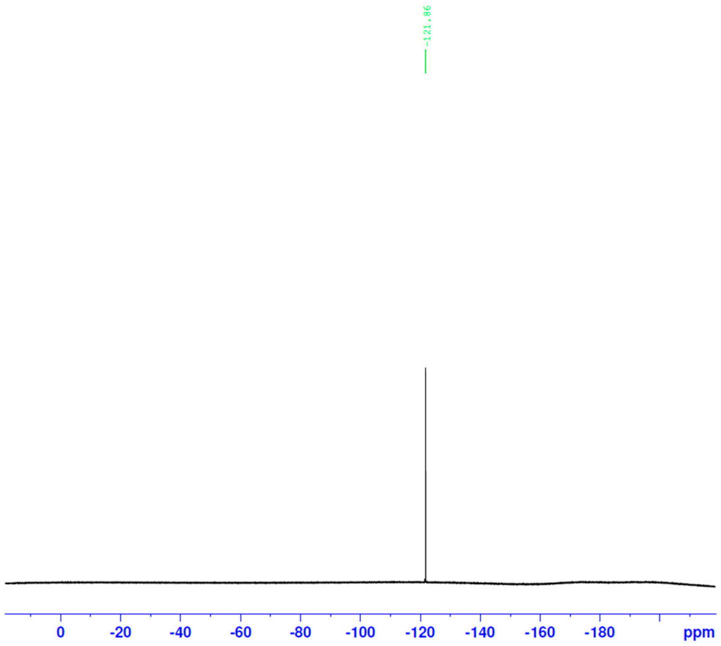

2D NOESY NMR (DMSO-d6)

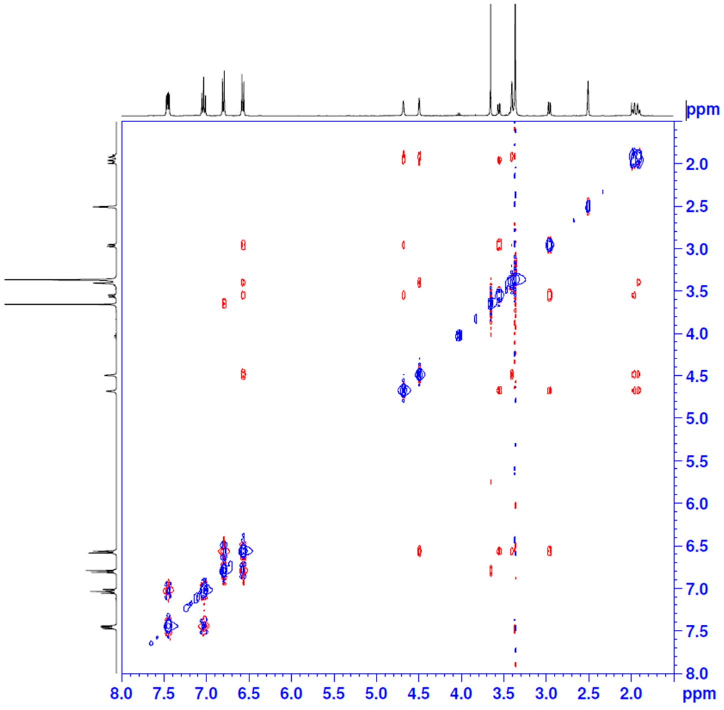

(1*R*,4*R*)-*N*-(3,4-Difluorophenyl)-5-(4-fluorophenyl)-2,5-diazabicyclo[2.2.1]heptane-2-carboxamide (2l)

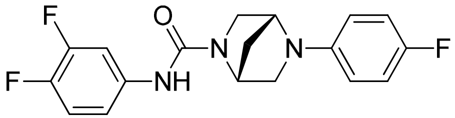

Calculated HRMS [M + H] + 348.1324

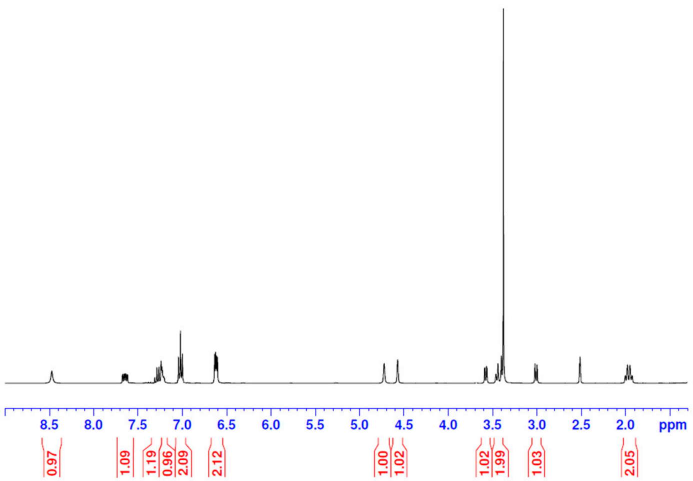

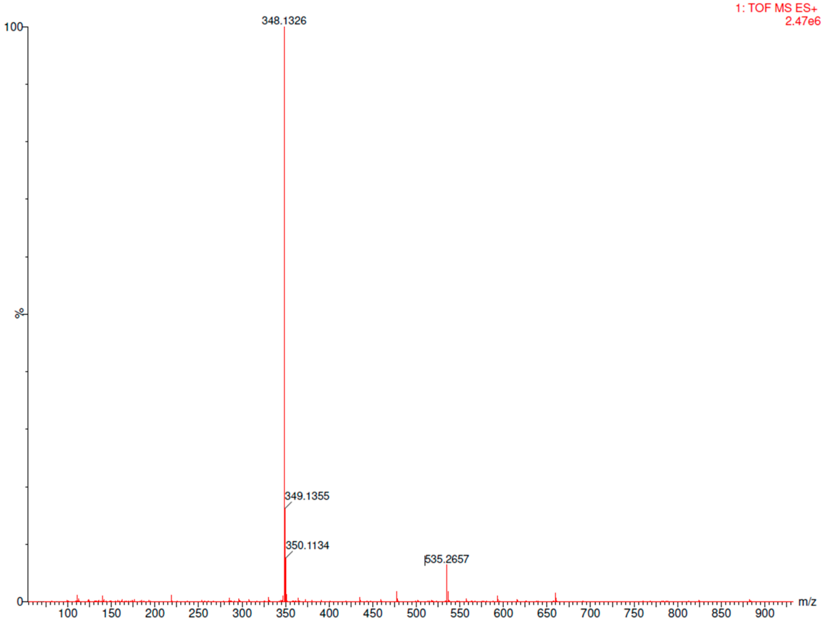

(1*R*,4*R*)-*N*-(3,4-Difluorophenyl)-5-(4-fluorophenyl)-2,5-diazabicyclo[2.2.1]heptane-2-carboxamide (2l)

2D COSY NMR (DMSO-d6)

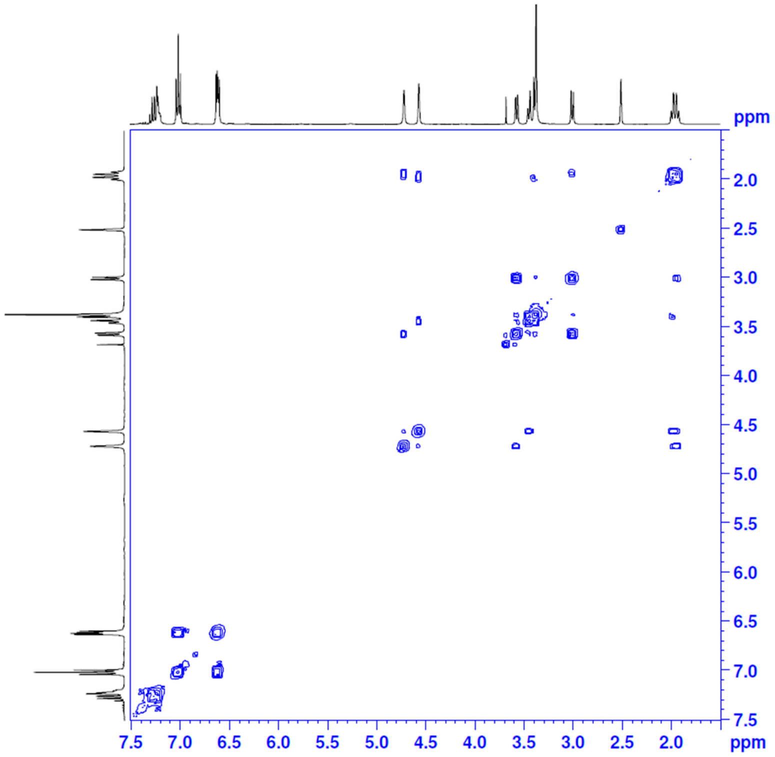

2D NOESY NMR (DMSO-d6)

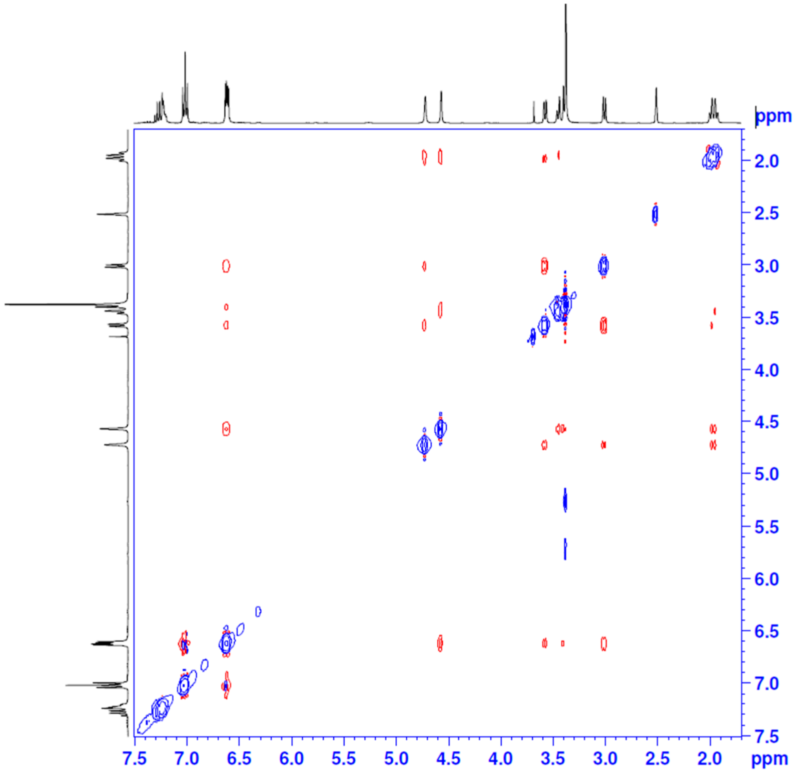

(1*R*,4*R*)-*N*-(2,4-Difluorophenyl)-5-(4-methoxyphenyl)-2,5-diazabicyclo[2.2.1]heptane-2-carboxamide (2m)

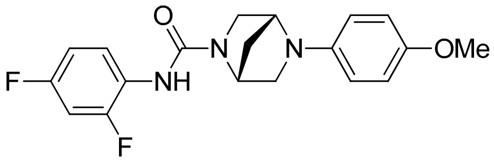

1H NMR (DMSO-d6)

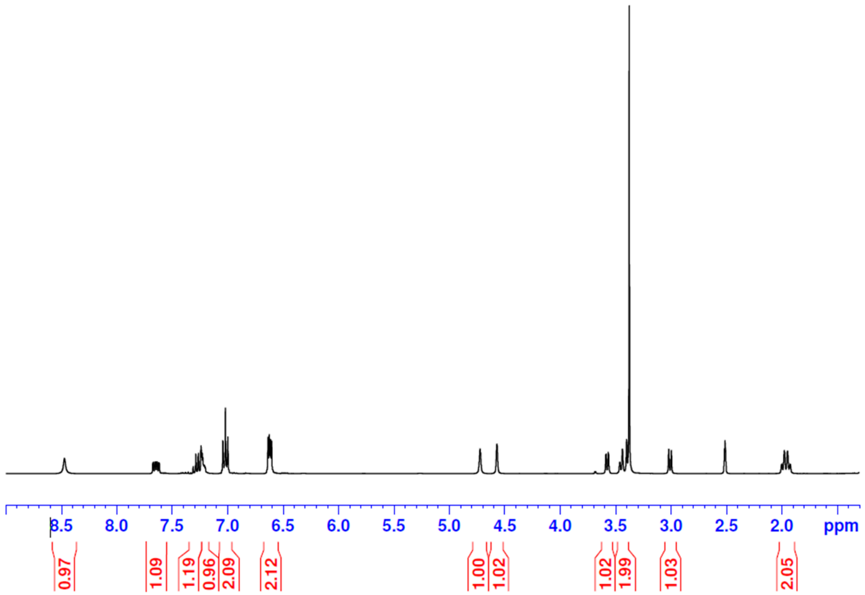

Calculated HRMS [M + H] + 360.1524

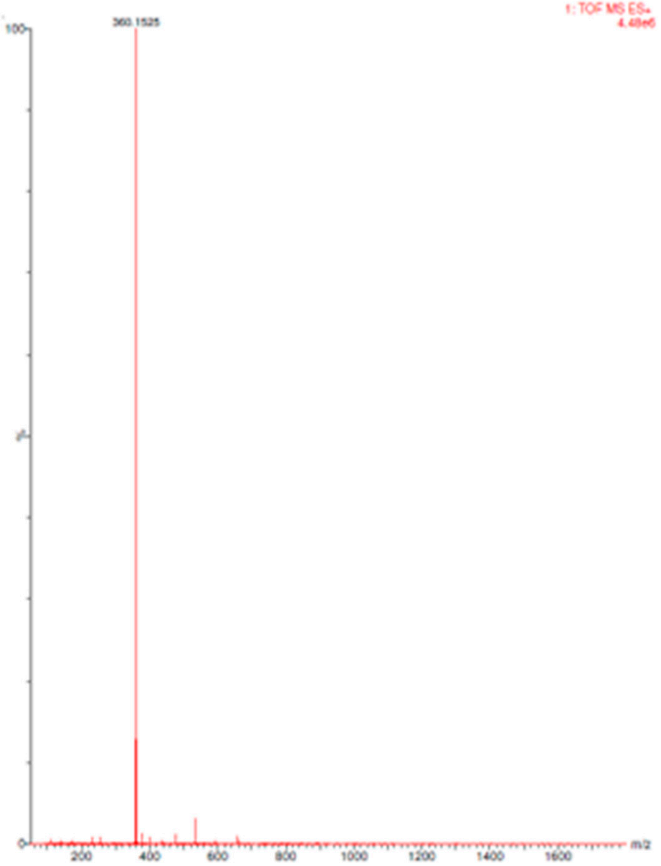

(1*R*,4*R*)-*N*-(2,4-Difluorophenyl)-5-(4-methoxyphenyl)-2,5-diazabicyclo[2.2.1]heptane-2-carboxamide (2m)

2D COSY NMR (DMSO-d6)

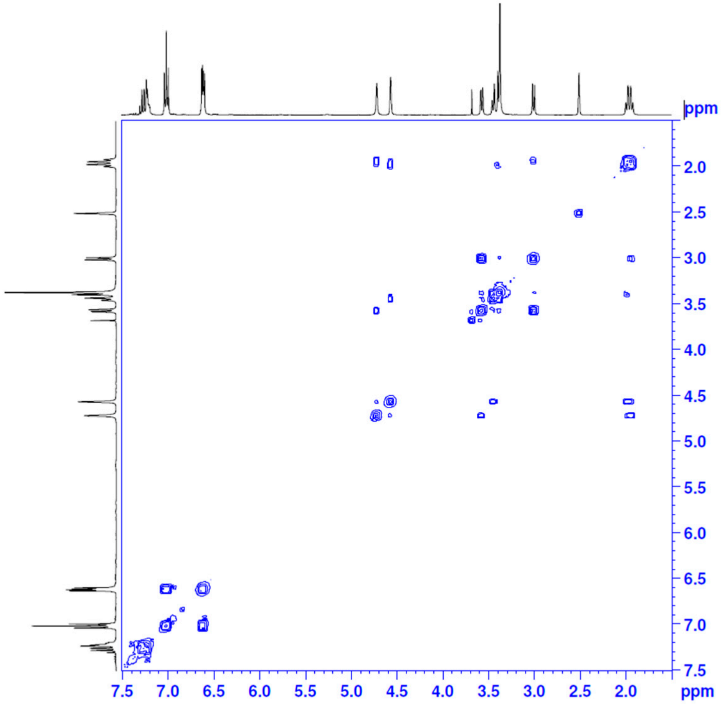

Purity: (LC, t_{min} = 3.27) 96.99%

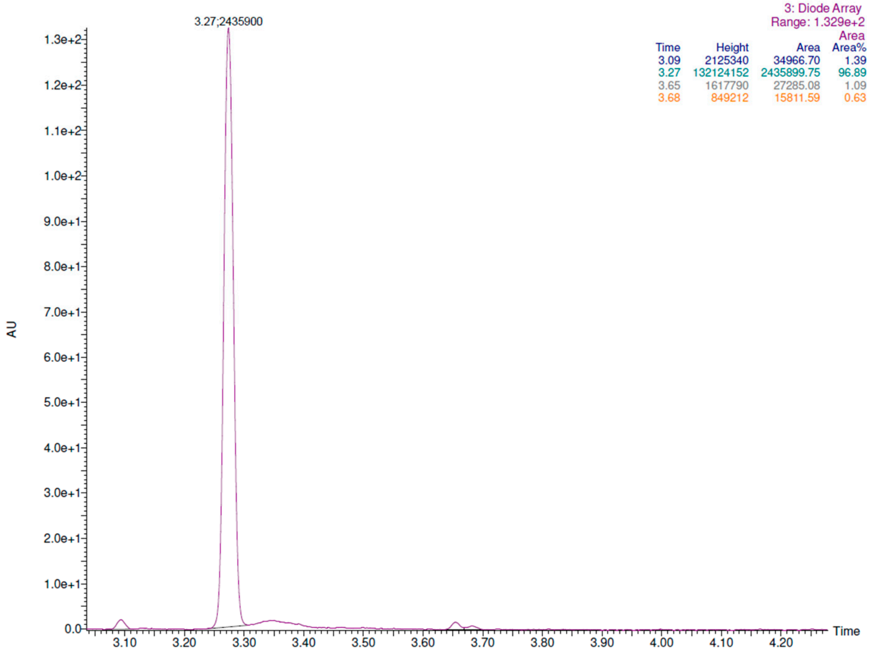

(1*R*,4*R*)-*N*,5-bis(4-Methoxyphenyl)-2,5-diazabicyclo[2.2.1]heptane-2-carboxamide (2n)

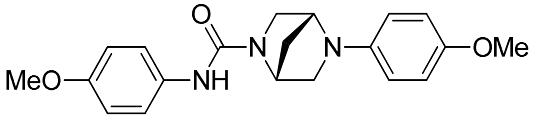

1H NMR (DMSO-d6)

Calculated HRMS [M + H] + 354.1818

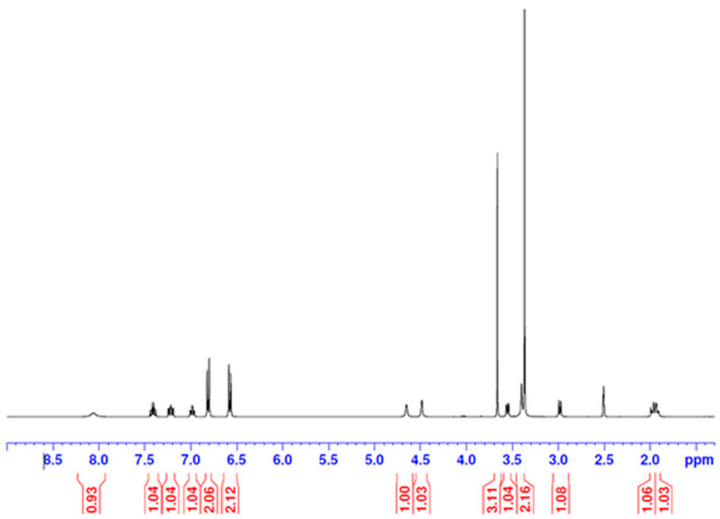

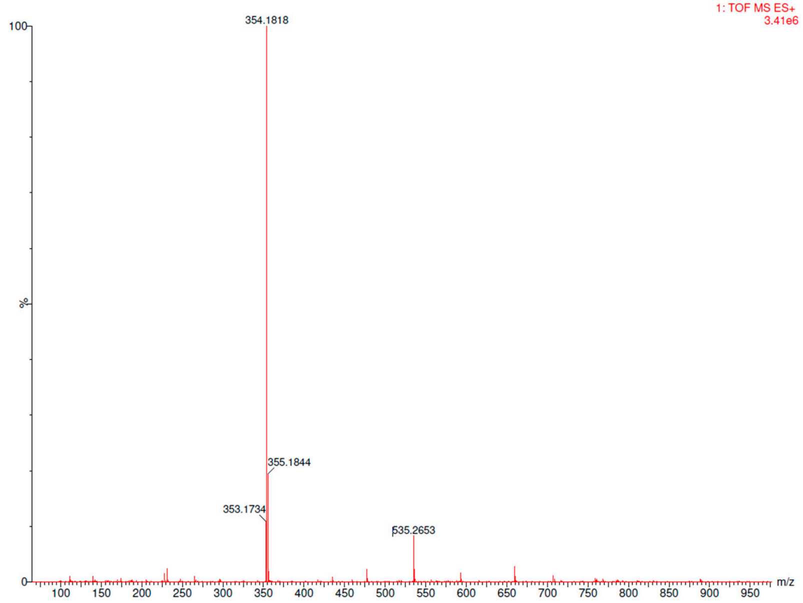

(1*R*,4*R*)-*N*,5-bis(4-Methoxyphenyl)-2,5-diazabicyclo[2.2.1]heptane-2-carboxamide (2n)

2D COSY NMR (DMSO-d6)

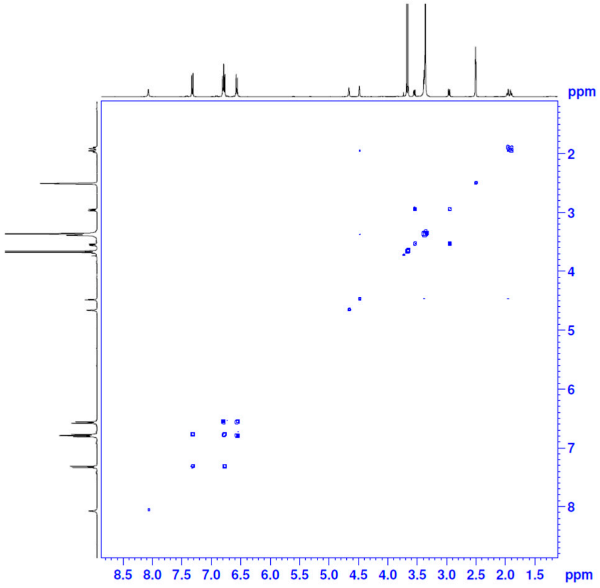

2D NOESY NMR (DMSO-d6)

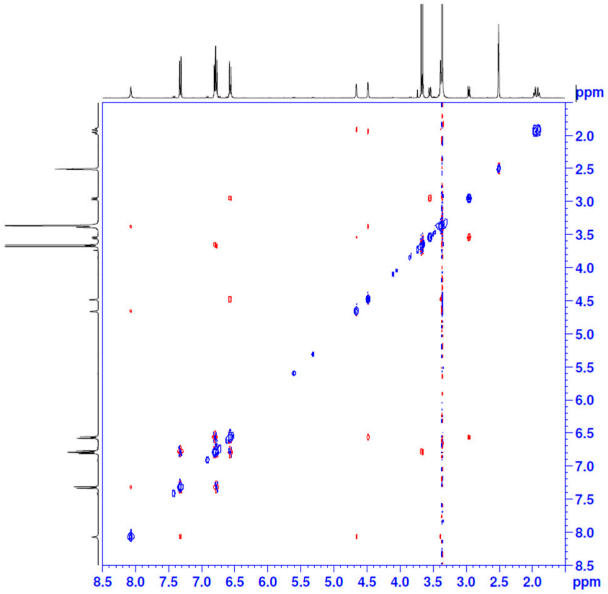

(1*R*,4*R*)-*N*-(4-Cyanophenyl)-5-(4-methoxyphenyl)-2,5-diazabicyclo[2.2.1]heptane-2-carboxamide (2o)

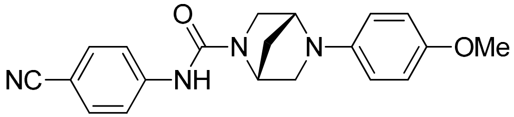

^{1}H NMR (DMSO-d6)

Calculated HRMS [M + H] + 349.1665

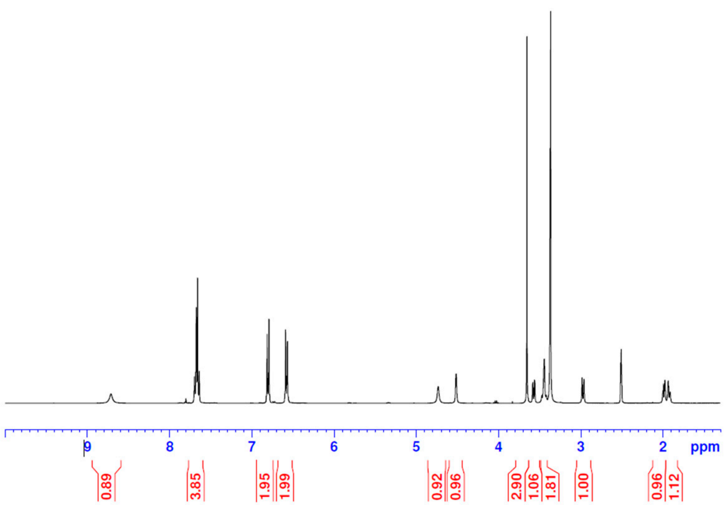

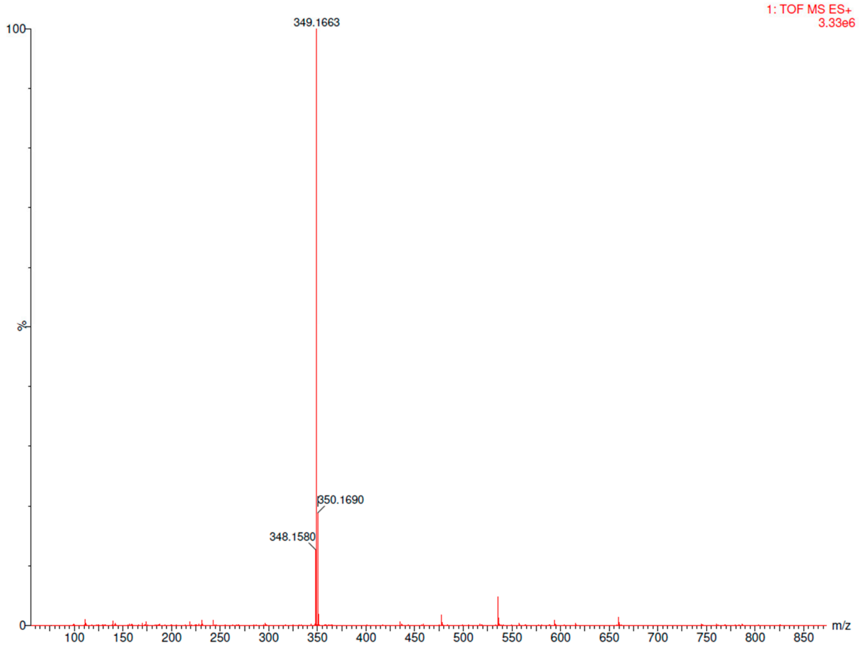

(1*R*,4*R*)-*N*-(4-Cyanophenyl)-5-(4-methoxyphenyl)-2,5-diazabicyclo[2.2.1]heptane-2-carboxamide (2o)

2D COSY NMR (DMSO-d6)

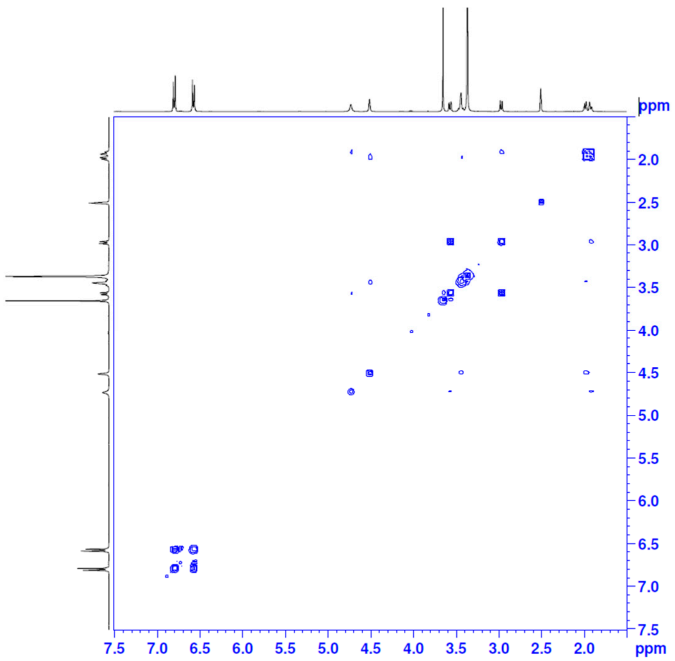

2D NOESY NMR (DMSO-d6)

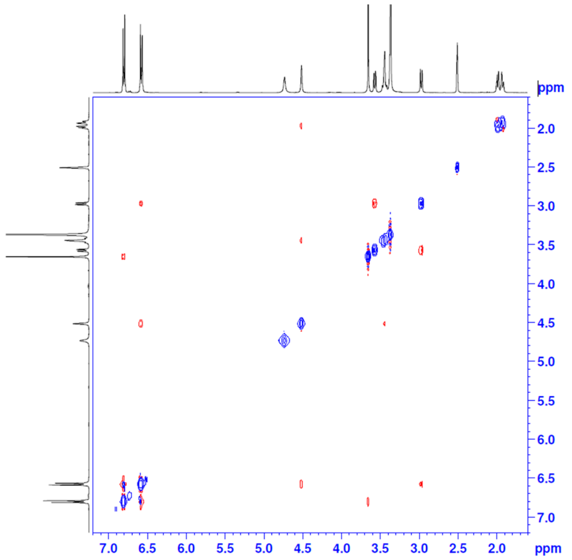

(1*R*,4*R*)-5-(4-Methoxyphenyl)-N-(4-(trifluoromethyl)phenyl)-2,5-diazabicyclo[2.2.1]heptane-2-carboxamide (2p)

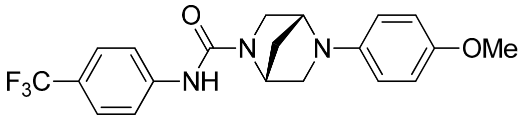

^{1}H NMR (DMSO-d6)

Calculated HRMS [M + H] + 392.1586

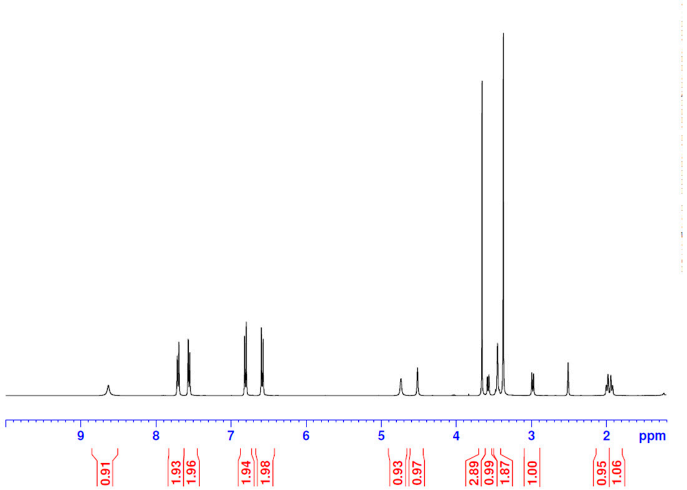

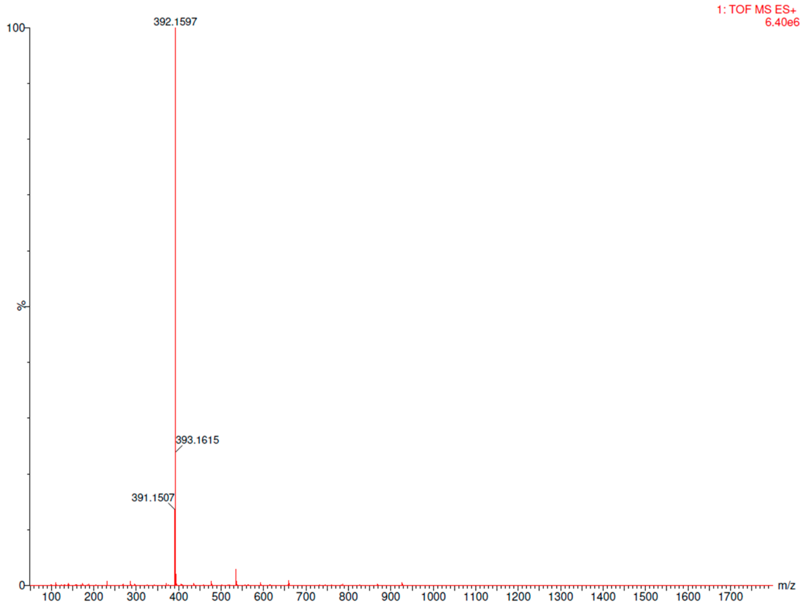

(1*R*,4*R*)-5-(4-Methoxyphenyl)-N-(4-(trifluoromethyl)phenyl)-2,5-diazabicyclo[2.2.1]heptane-2-carboxamide (2p)

2D COSY NMR (DMSO-d6)

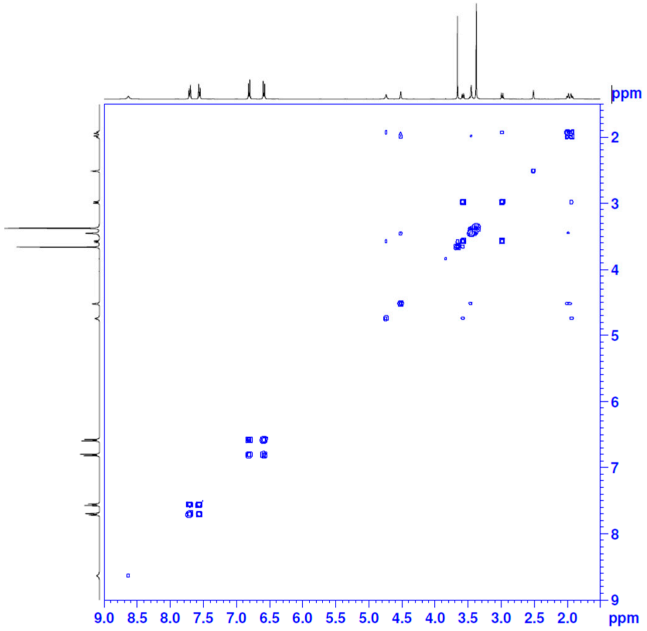

2D NOESY NMR (DMSO-d6)

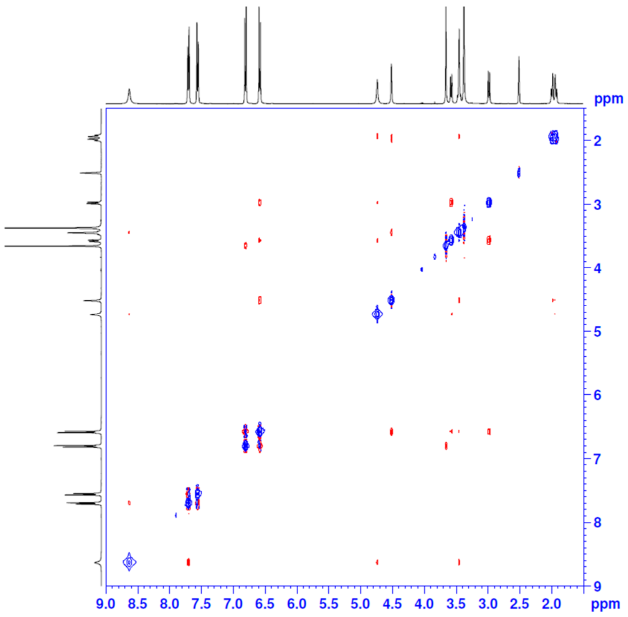

(1*R*,4*R*)-*N*-(*tert*-Butyl)-5-(4-methoxyphenyl)-2,5-diazabicyclo[2.2.1]heptane-2-carboxamide (2q)

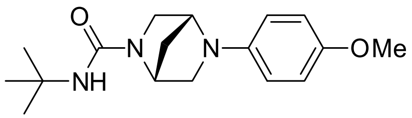

^{1}H NMR (DMSO-d6)

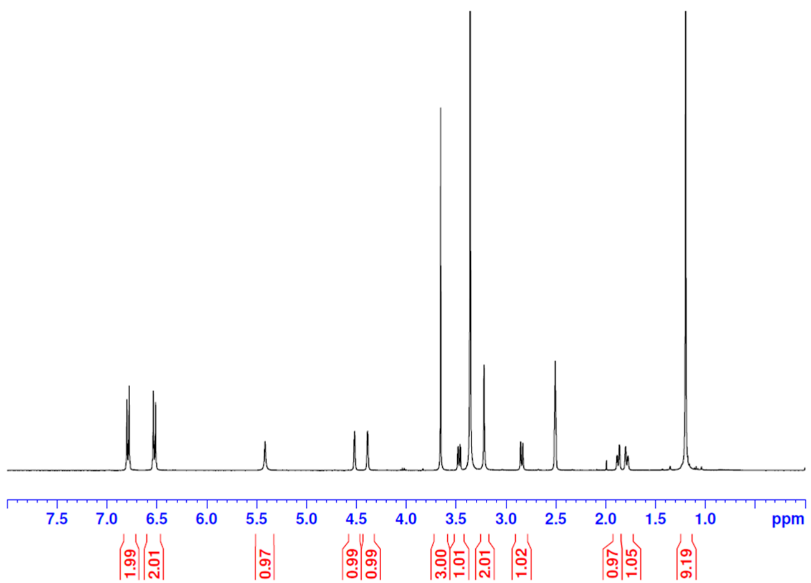

Calculated HRMS [M + H] + 304.2025

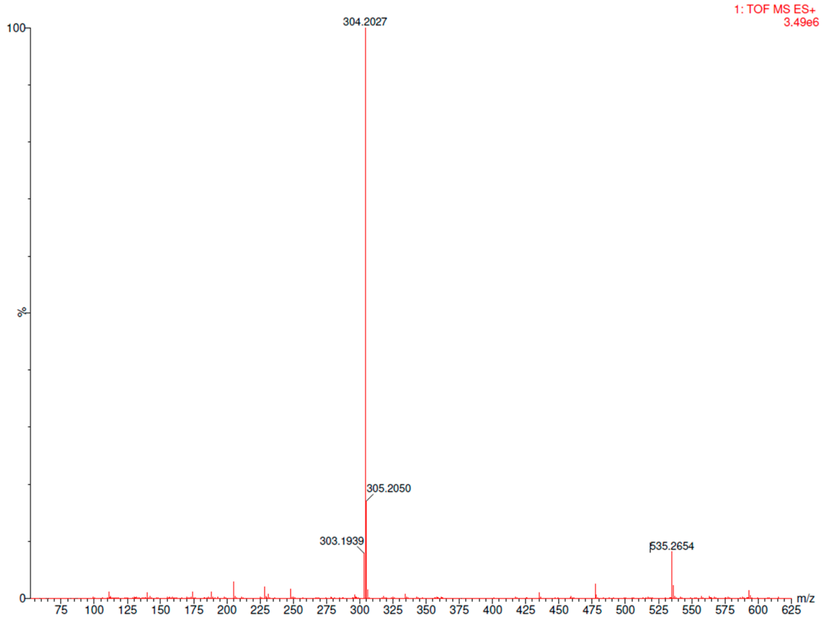

(1*R*,4*R*)-*N*-(*tert*-Butyl)-5-(4-methoxyphenyl)-2,5-diazabicyclo[2.2.1]heptane-2-carboxamide (2q)

Purity: (LC, t_{min} = 2.86) 99.36%

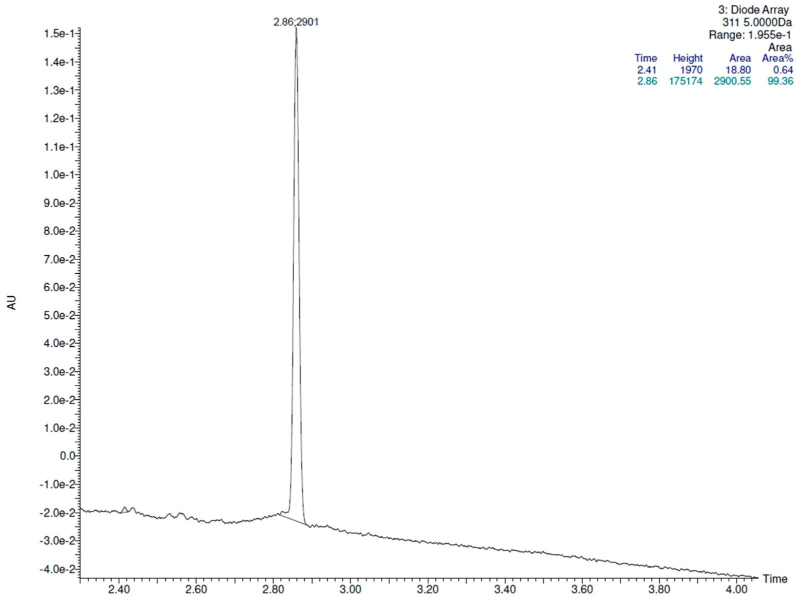

2D NOESY NMR (DMSO-d6)

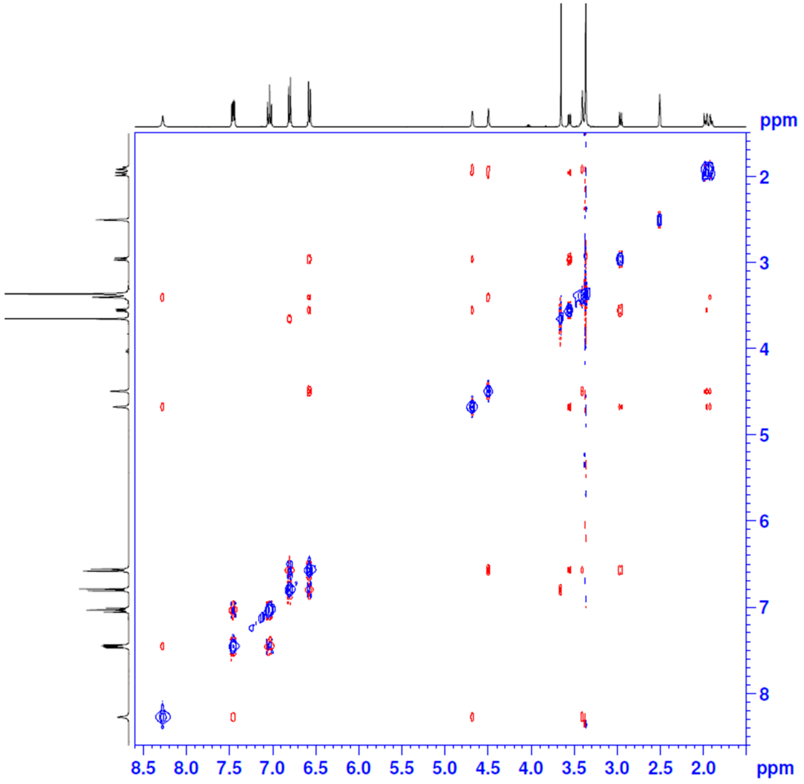

(1*R*,4*R*)-Cyclohexyl 5-(4-methoxyphenyl)-2,5-diazabicyclo[2.2.1]heptane-2-carboxylate (2r)

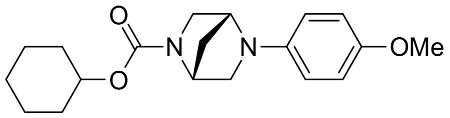

^{1}H NMR (DMSO-d6)

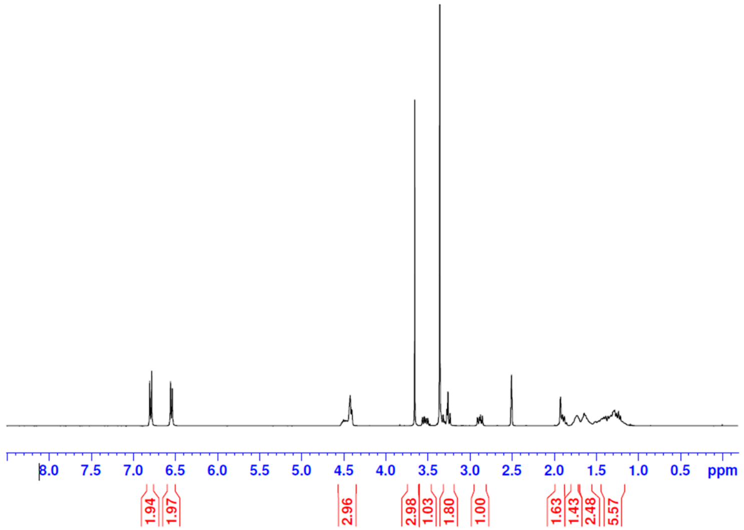

Calculated HRMS [M + H] + 331.2022

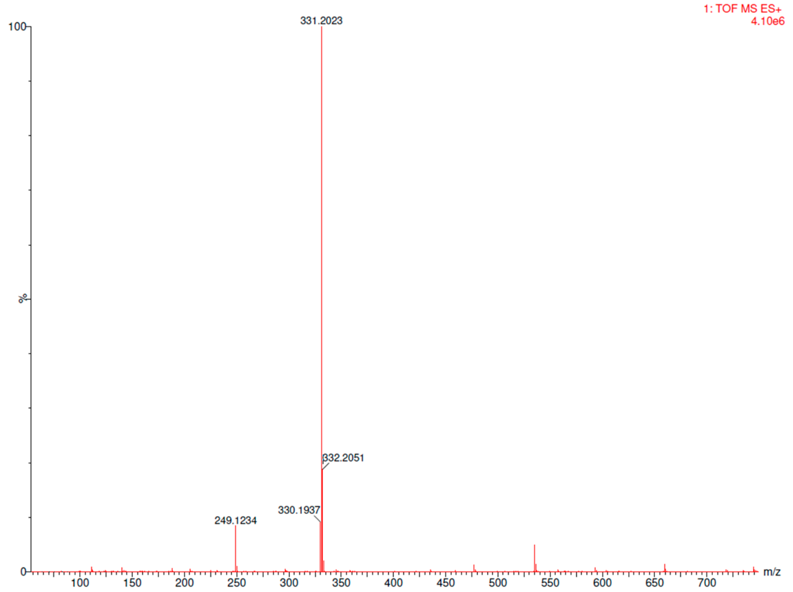

(1*R*,4*R*)-Cyclohexyl 5-(4-methoxyphenyl)-2,5-diazabicyclo[2.2.1]heptane-2-carboxylate (2r)

Purity: (LC, t_{min} = 3.66) 98.75%

UV max: 249.45

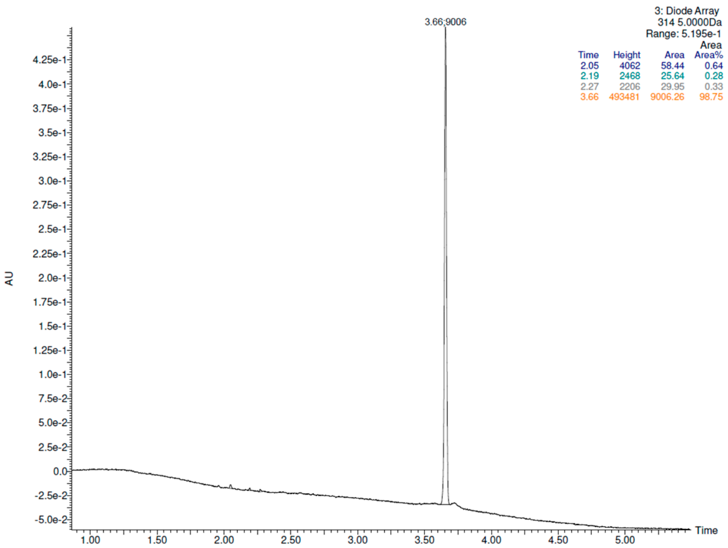

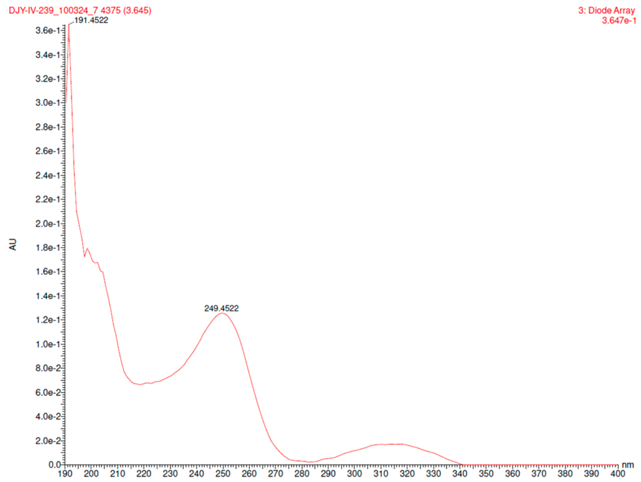

(1*R*,4*R*)-*N*-(5-Chloro-2-methoxyphenyl)-5-(3,4-dimethoxyphenyl)-2,5-diazabicyclo[2.2.1]heptane-2-carboxamide (2s)

^{1}H NMR (DMSO-d6)

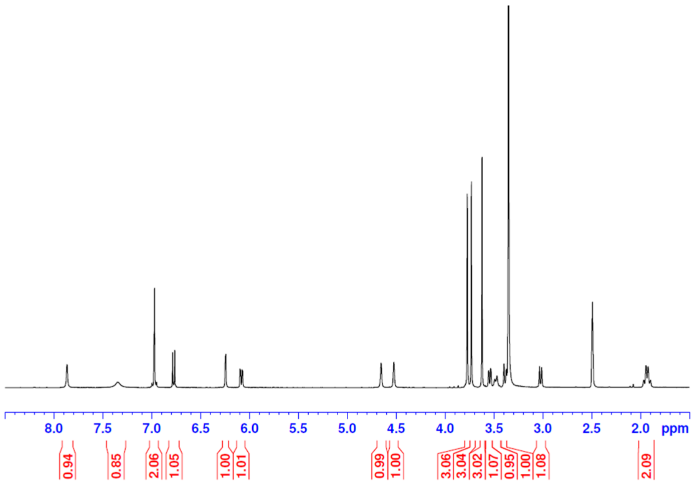

Calculated HRMS [M - H] - 416.1377

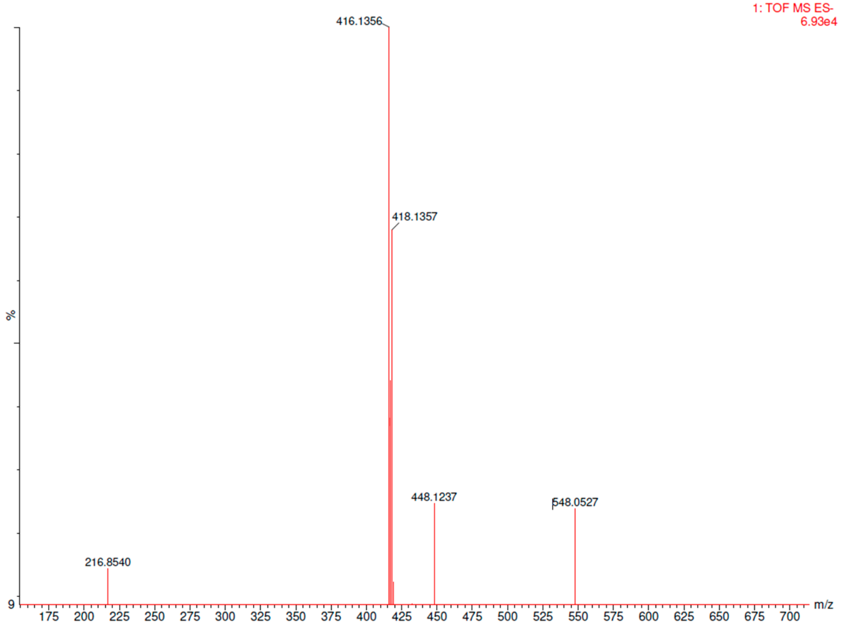

(1*R*,4*R*)-*N*-(5-Chloro-2-methoxyphenyl)-5-(3,4-dimethoxyphenyl)-2,5-diazabicyclo[2.2.1]heptane-2-carboxamide (2s)

2D COSY NMR (DMSO-d6)

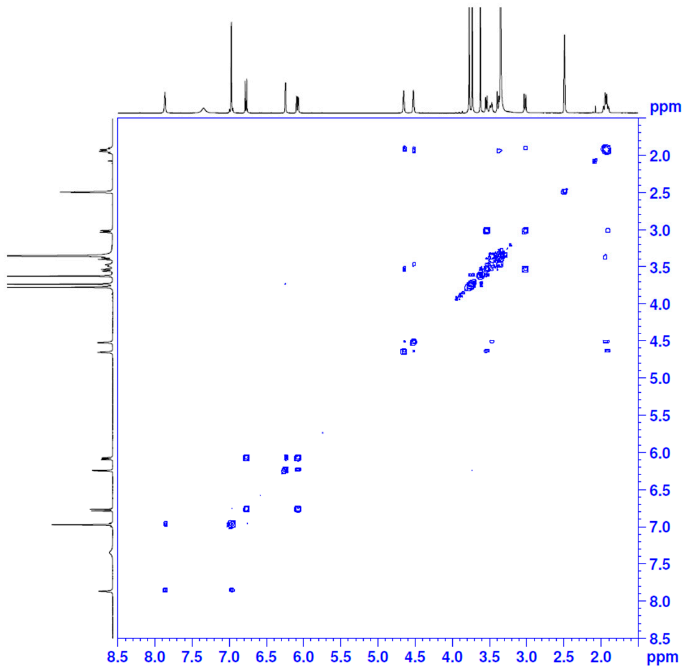

2D NOESY NMR (DMSO-d6)

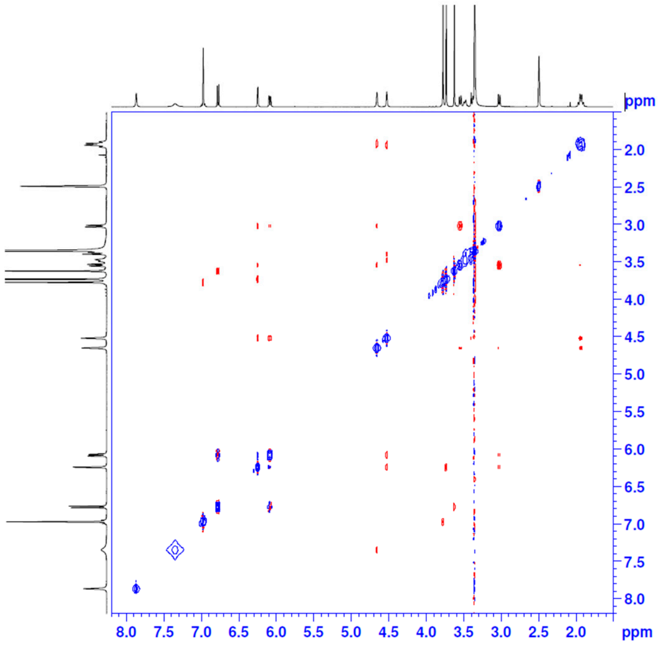

Figure S3. Analytical spectrums of Series 2 (**3a - b, 4a-k, 5**)

***N*-(3,4-Difluorophenyl)-3-(4-fluorophenyl)-1*H*-pyrrole-1-carboxamide (3a)**

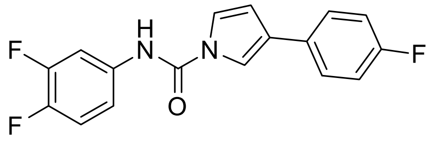

^{1}H NMR (CDCl$_3$)

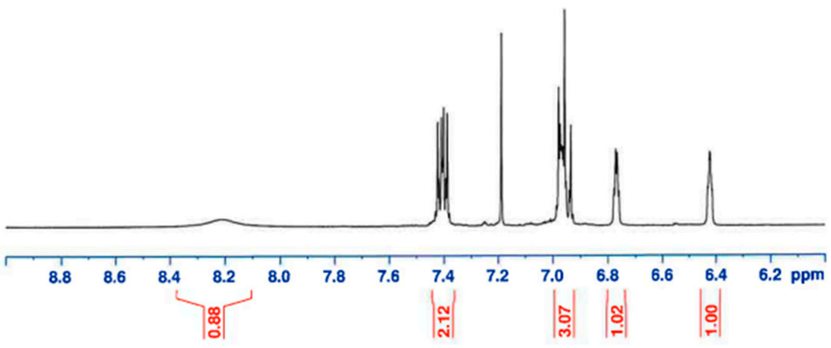

Calculated HRMS [M + H] + 317.0902

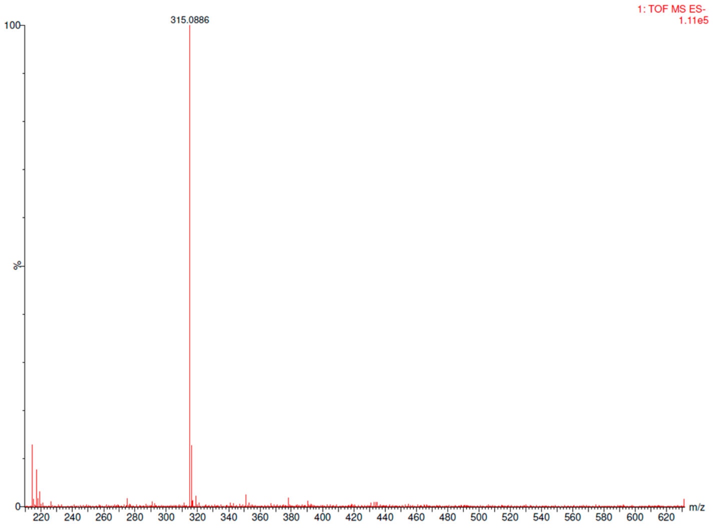

N-(3,4-Difluorophenyl)-3-(4-fluorophenyl)-1H-pyrrole-1-carboxamide (3a)

^{19}F NMR (CDCl$_3$)

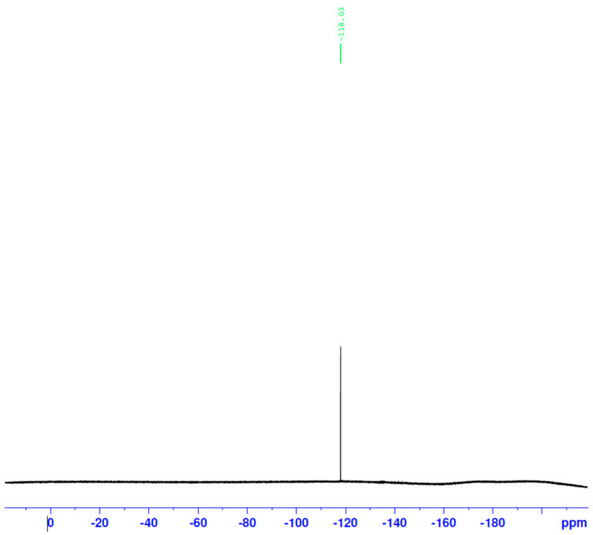

Purity: (LC, t_{min} = 3.72) 98.64%

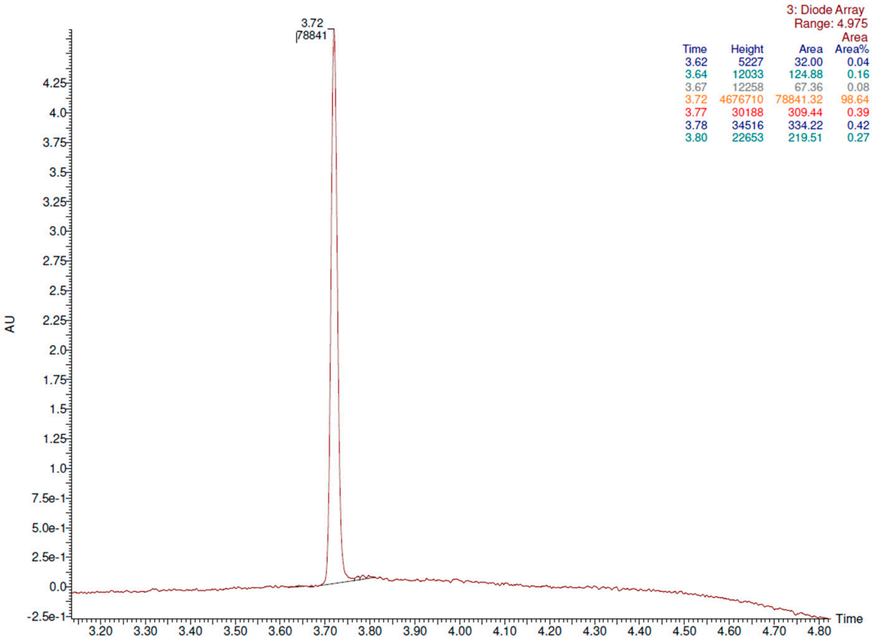

_N_-(3,4-Difluorophenyl)-4-(4-methoxyphenyl)-1_H_-pyrazole-1-carboxamide (3b)

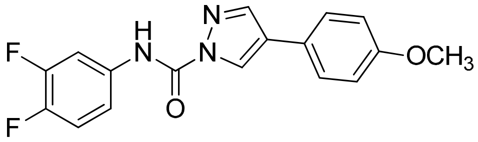

^{1}H NMR (DMSO-d6)

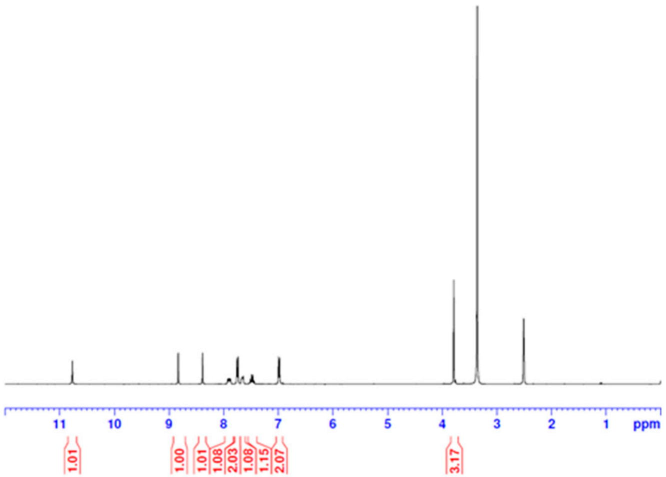

^{19}F NMR (DMSO-d6)

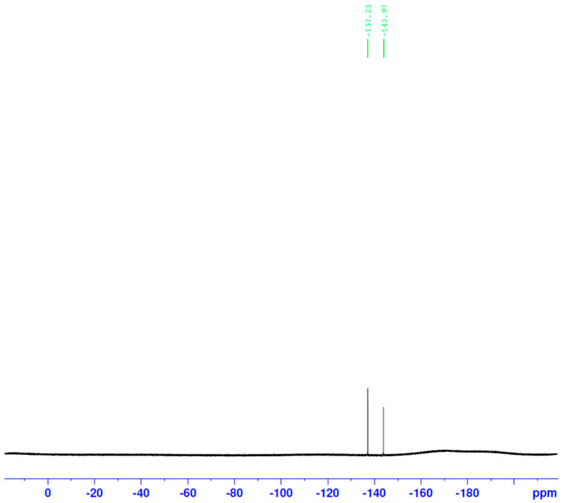

N-(3,4-Difluorophenyl)-4-(4-methoxyphenyl)-1H-pyrazole-1-carboxamide (3b)

2D COSY NMR (DMSO-d6)

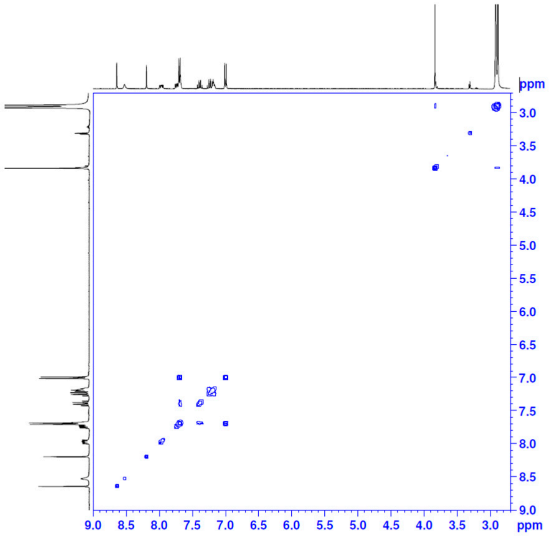

2D NOESY NMR (DMSO-d6)

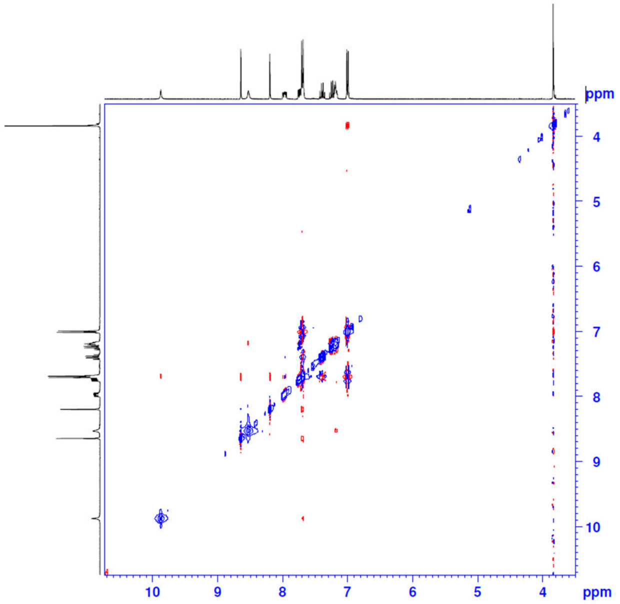

1,3-bis(3,4-Difluorophenyl)urea (4a)

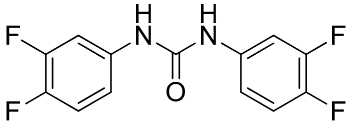

^{1}H NMR (DMSO-d_6)

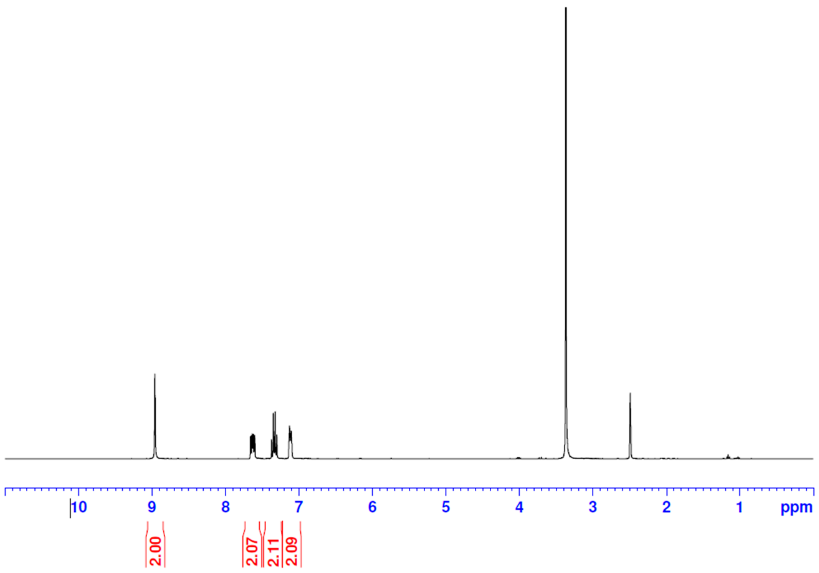

^{13}C NMR (DMSO-d_6)

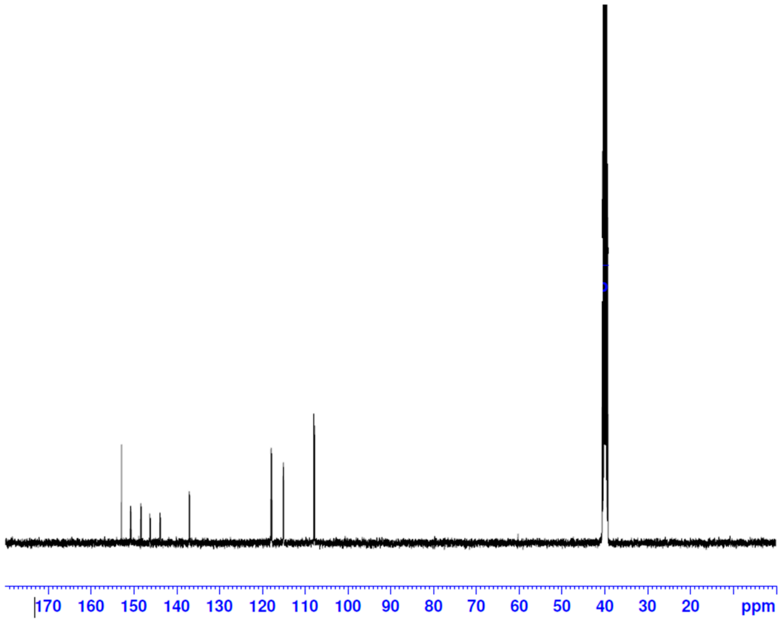

1,3-bis(3,4-Difluorophenyl)urea (4a)

^{19}F NMR (DMSO-d_6)

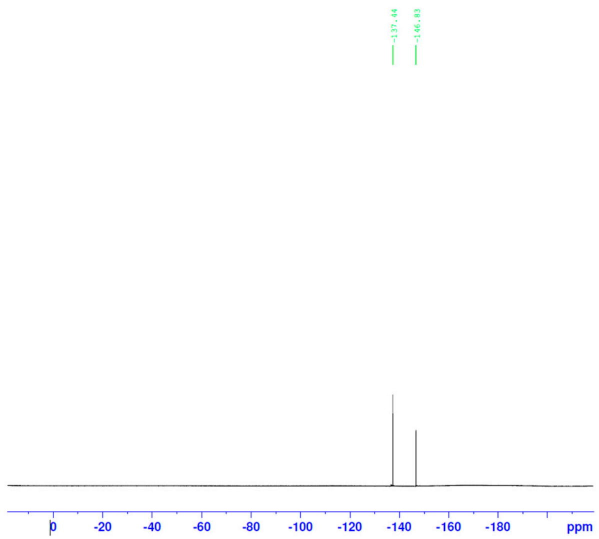

2D COSY NMR (DMSO-d6)

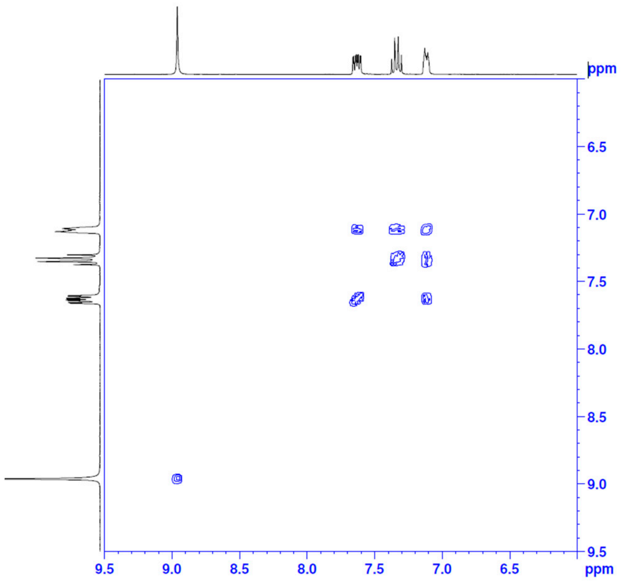

1-(3,4-Difluorophenyl)-3-(3,4,5-trifluorophenyl)urea (4b)

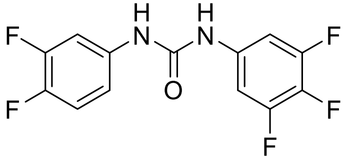

^{1}H NMR (DMSO-d$_6$)

Calculated HRMS [M + H] + 303.0557

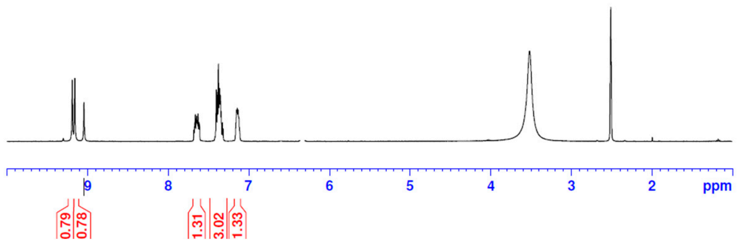

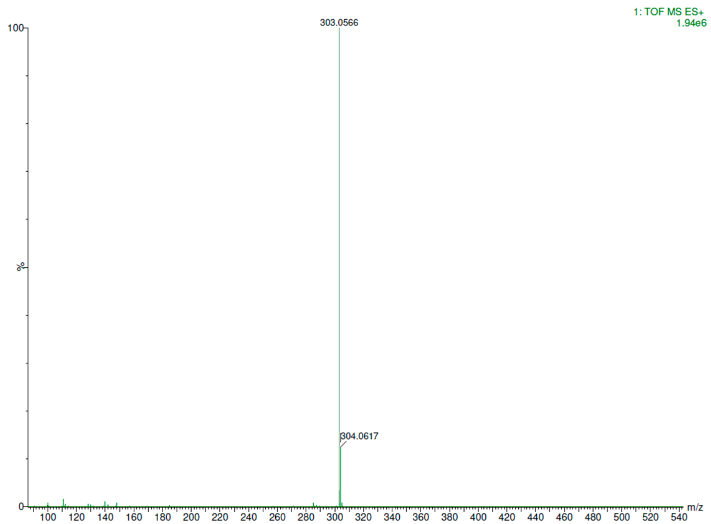

1-(3,4-Difluorophenyl)-3-(3,4,5-trifluorophenyl)urea (4b)

^{13}C NMR (DMSO-d_6)

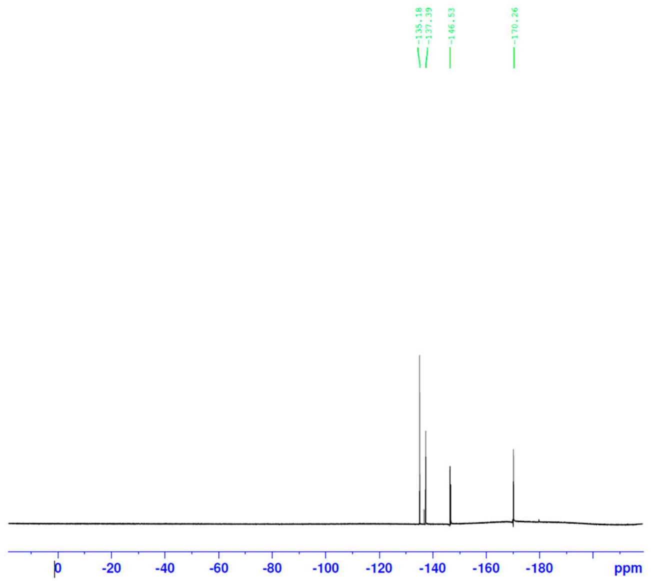

Purity: (LC, t_{min} = 3.68) 99.35%

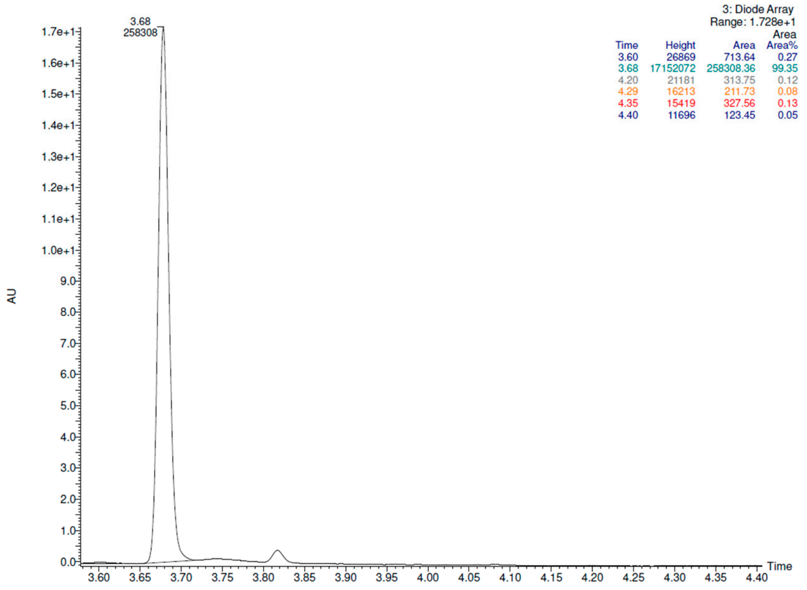

1-(4-Chloro-3-fluorophenyl)-3-(3,4-difluorophenyl)urea (4c)

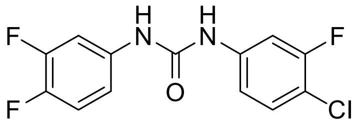

^{1}H NMR (DMSO-d_6)

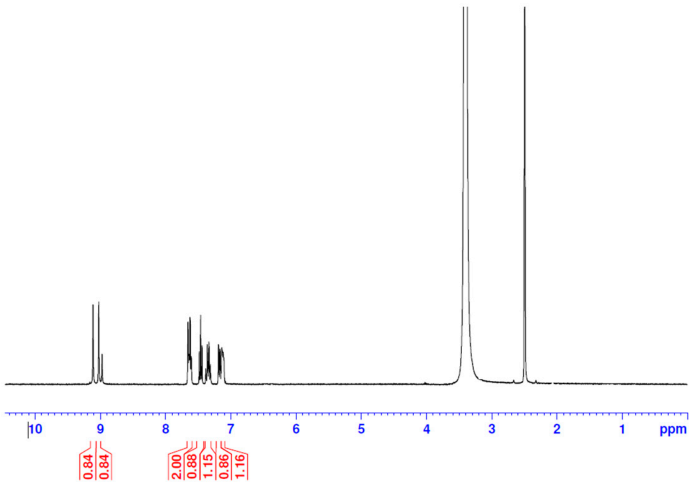

^{19}F NMR (DMSO-d_6)

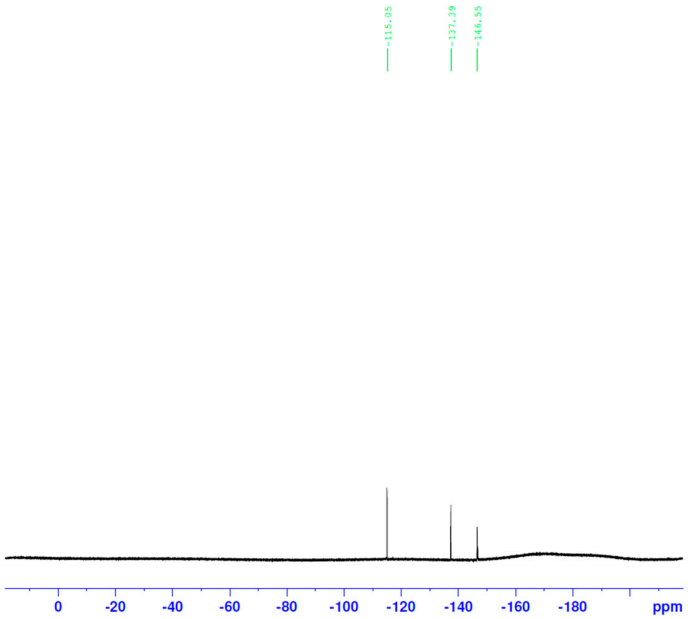

1-(4-Chloro-3-fluorophenyl)-3-(3,4-difluorophenyl)urea (4c)

Calculated HRMS [M + H] + 301.0356

Purity (LC, t min = 3.70) 98.65%

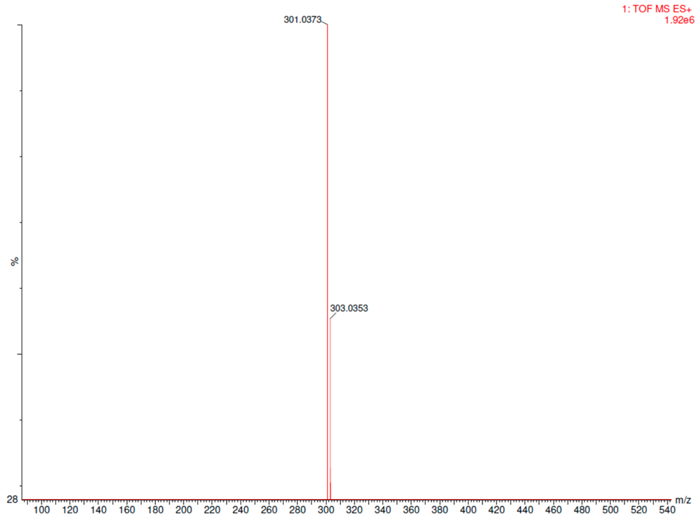

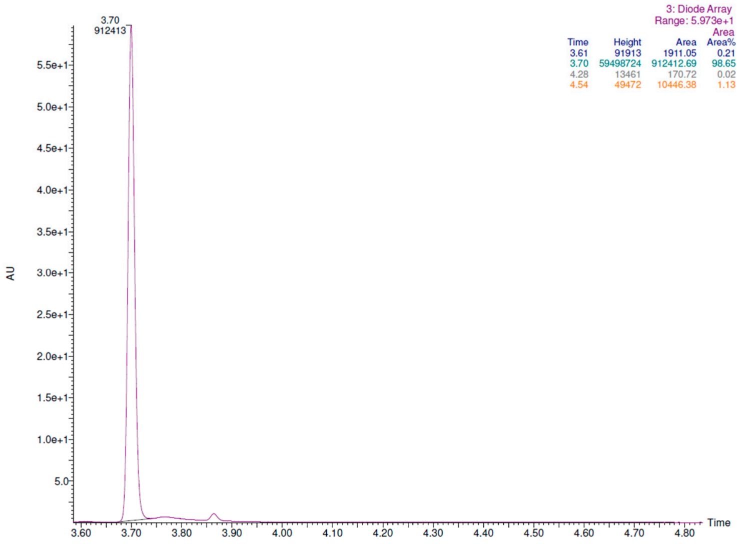

1,3-bis(3,4,5-Trifluorophenyl)urea (4d)

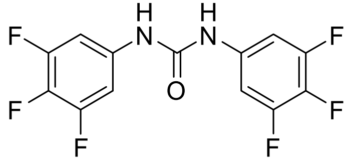

Calculated HRMS [M - H] - 319.0306

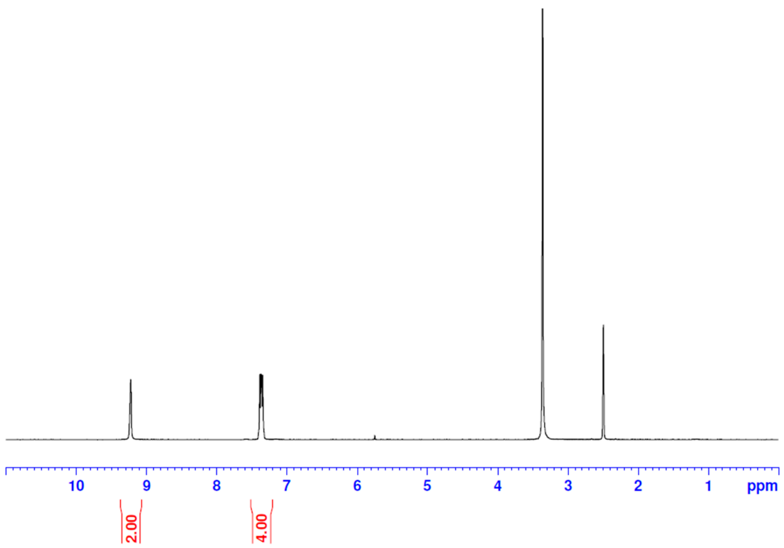

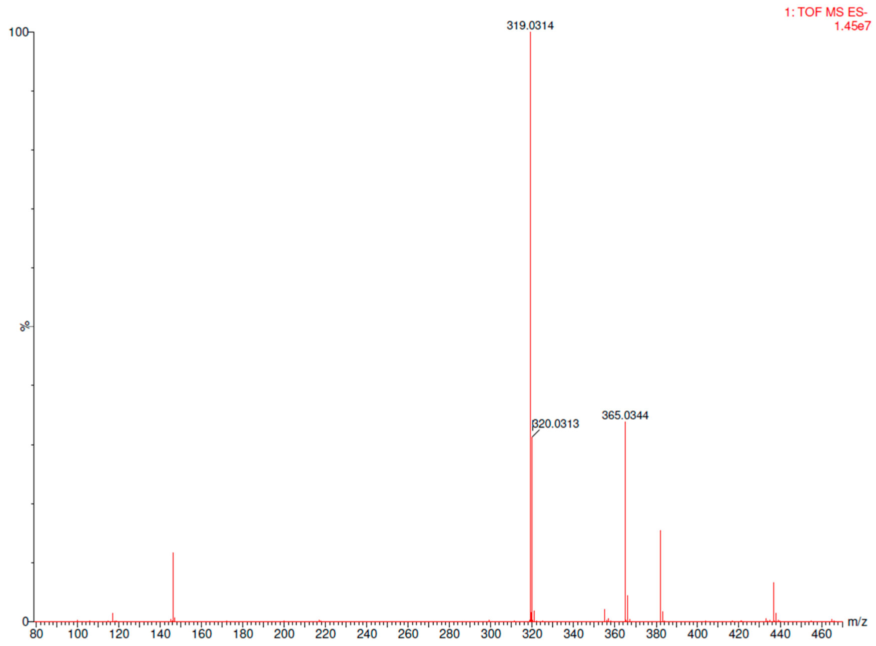

1,3-bis(3,4,5-Trifluorophenyl)urea (4d)

Purity: (LC, t_{min} = 3.62) 97.27%

UV max: 253.45, 190.45

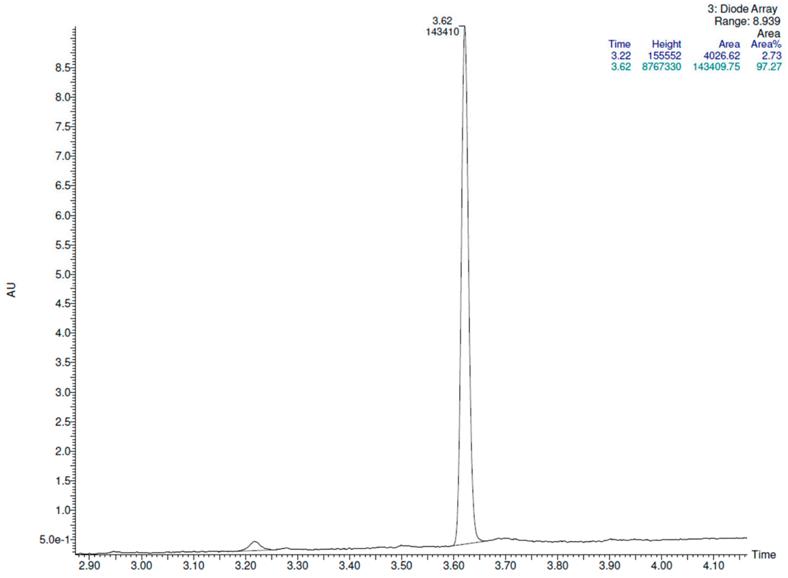

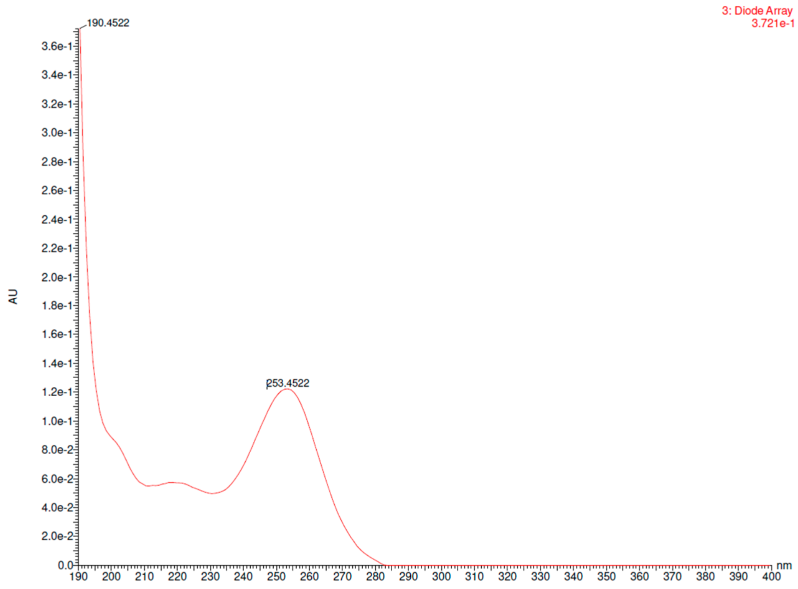

1-(3,4-Difluorophenyl)-3-(4-methoxyphenyl)urea (4e)

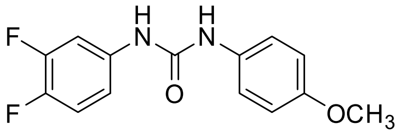

Calculated HRMS [M - H] - 319.0306

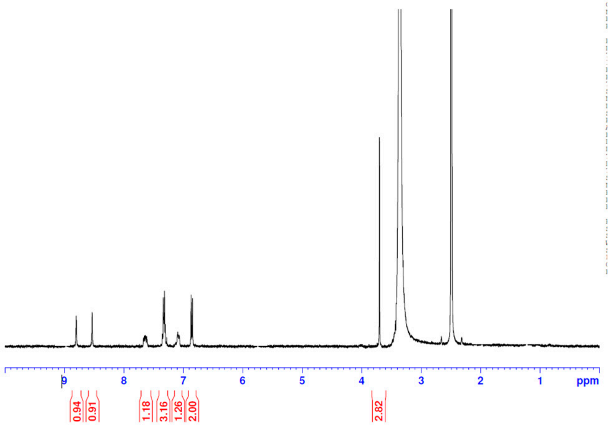

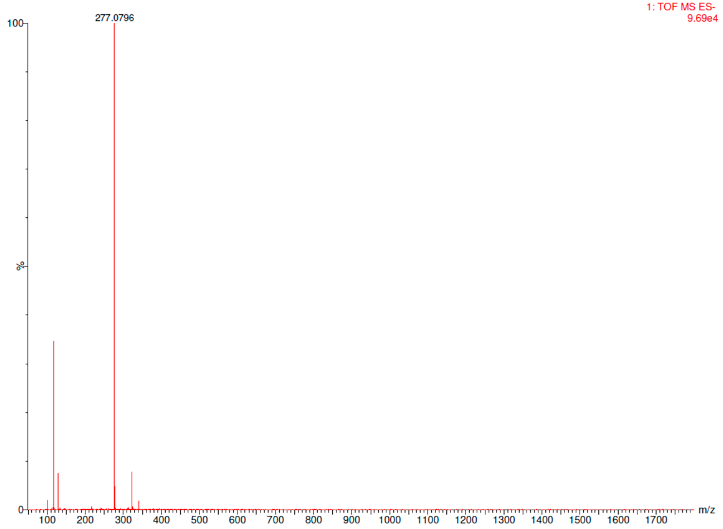

1-(3,4-Difluorophenyl)-3-(4-methoxyphenyl)urea (4e)

MS (ESI) *m/z* 279.17 [M + H]$^+$

UV max: 223.45.

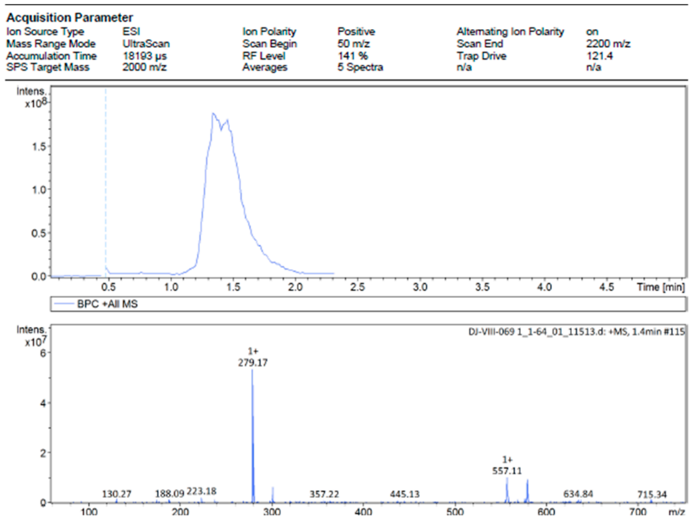

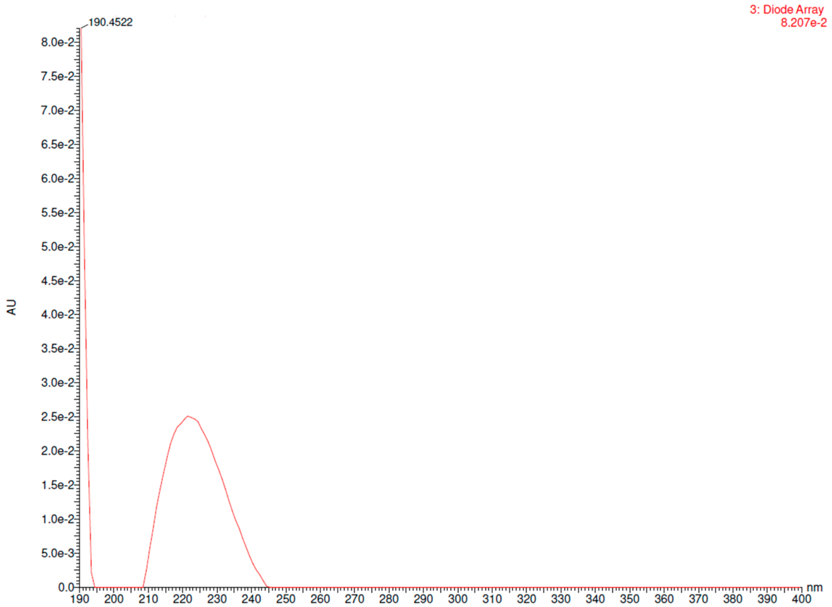

1,3-bis(4-Methoxyphenyl)urea (4f)

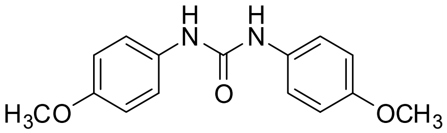

^{1}H NMR ($CDCl_3$)

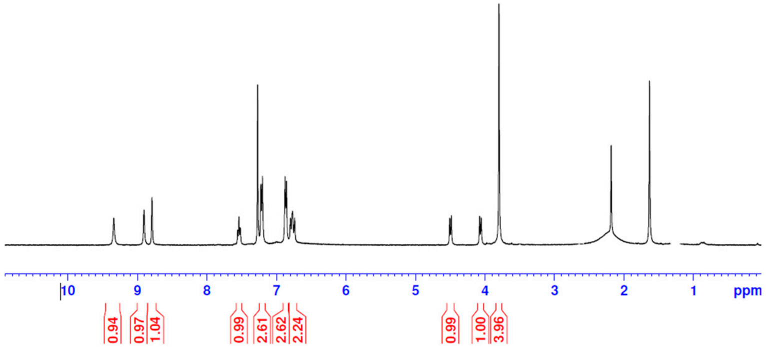

Calculated HRMS [M - H] $^-$ 271.1083

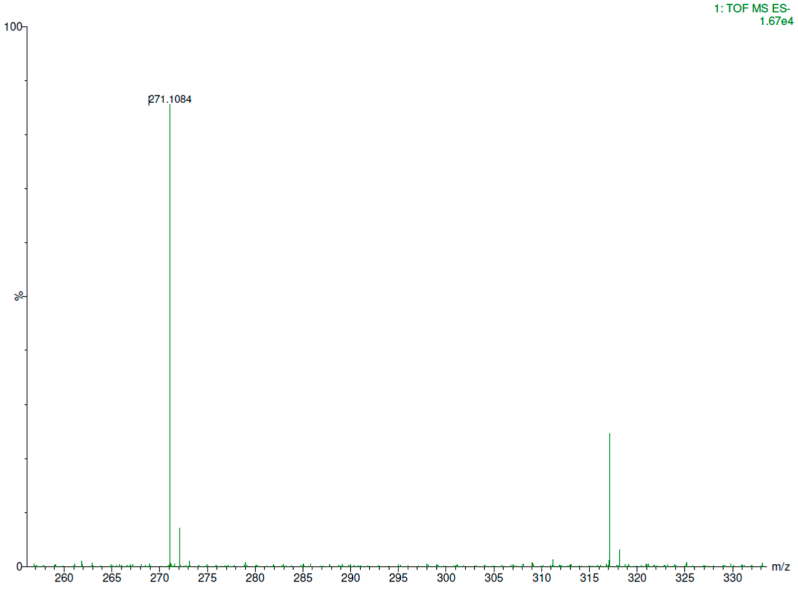

1,3-bis(4-Methoxyphenyl)urea (4f)

2D COSY NMR ($CDCl_3$)

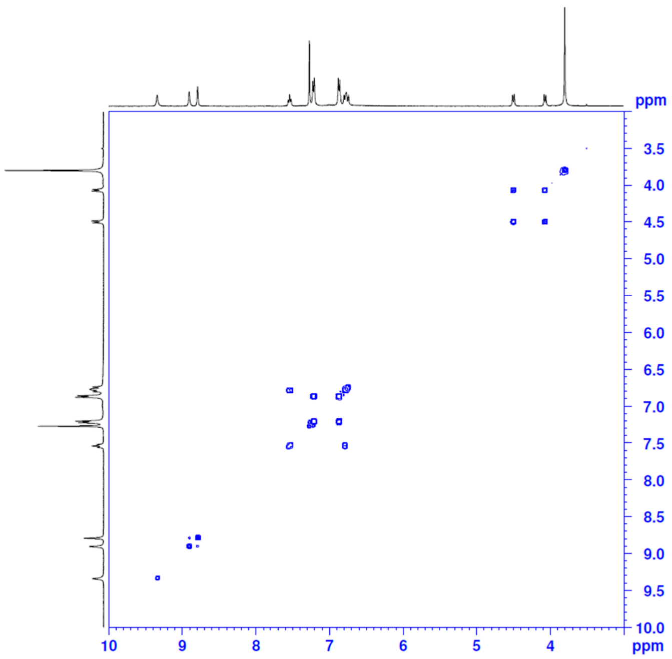

2D NOESY NMR ($CDCl_3$)

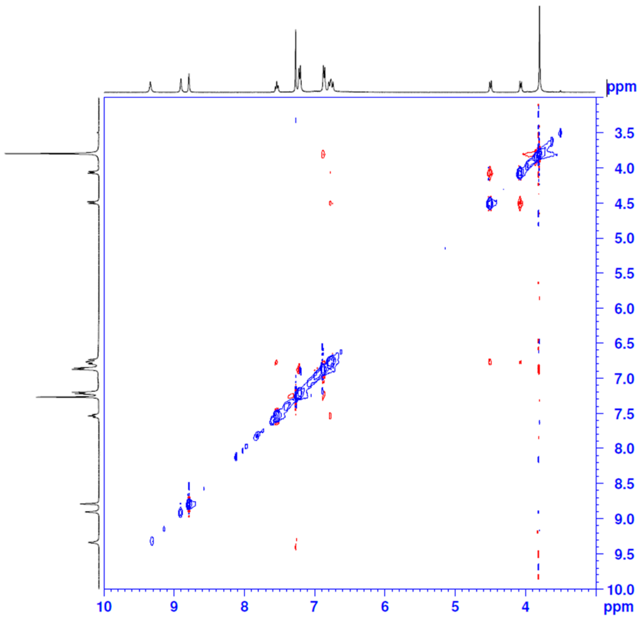

_N_-(3,4-Difluorophenyl)-4-fluoro-1_H_-indole-1-carboxamide (4g)

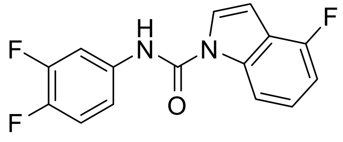

^{1}H NMR ($CDCl_3$)

Calculated HRMS [M - H] - 289.0589

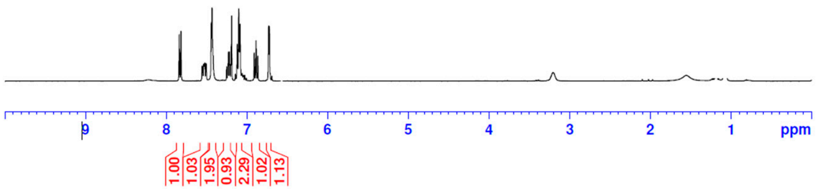

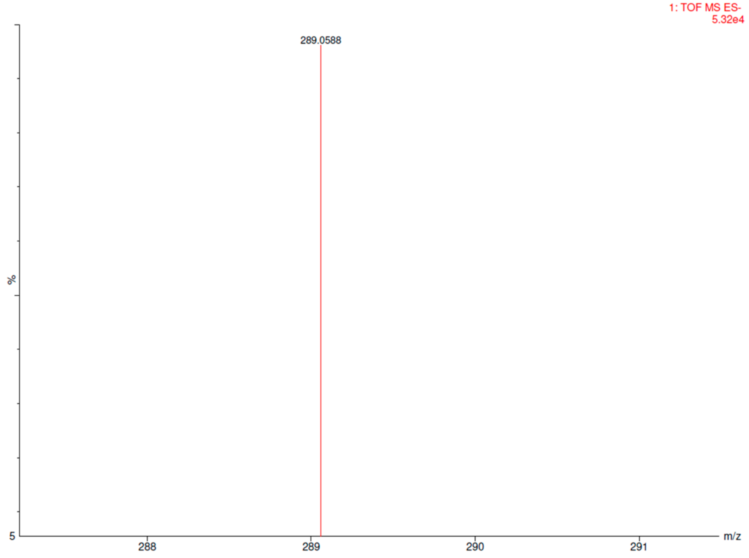

N-(3,4-Difluorophenyl)-4-fluoro-1H-indole-1-carboxamide (4g)

2D COSY NMR ($CDCl_3$)

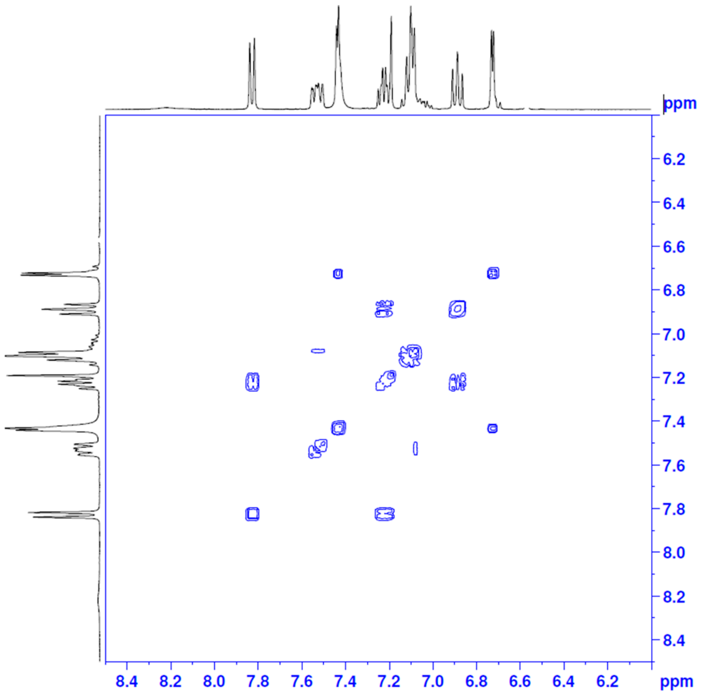

2D NOESY NMR ($CDCl_3$)

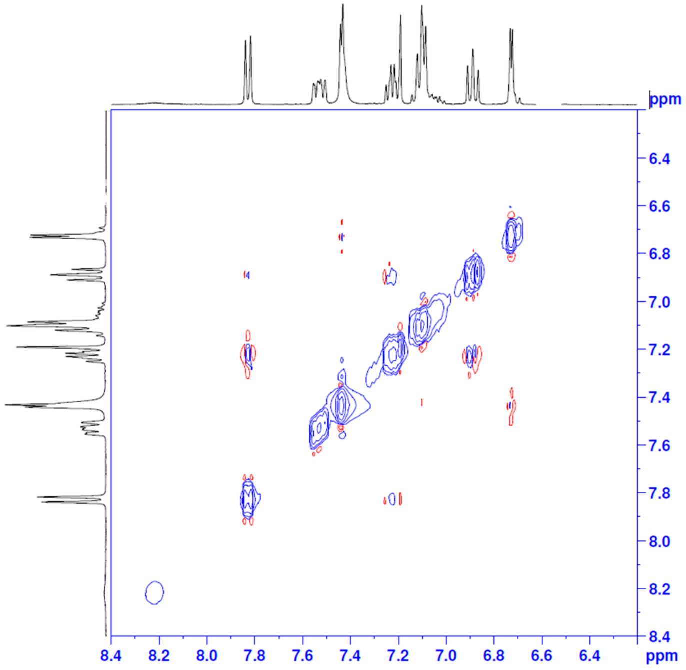

4-Cyano-*N*-(3,4-difluorophenyl)-1*H*-indole-1-carboxamide (4h)

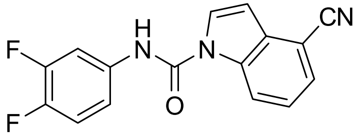

^{1}H NMR (DMSO-d$_6$)

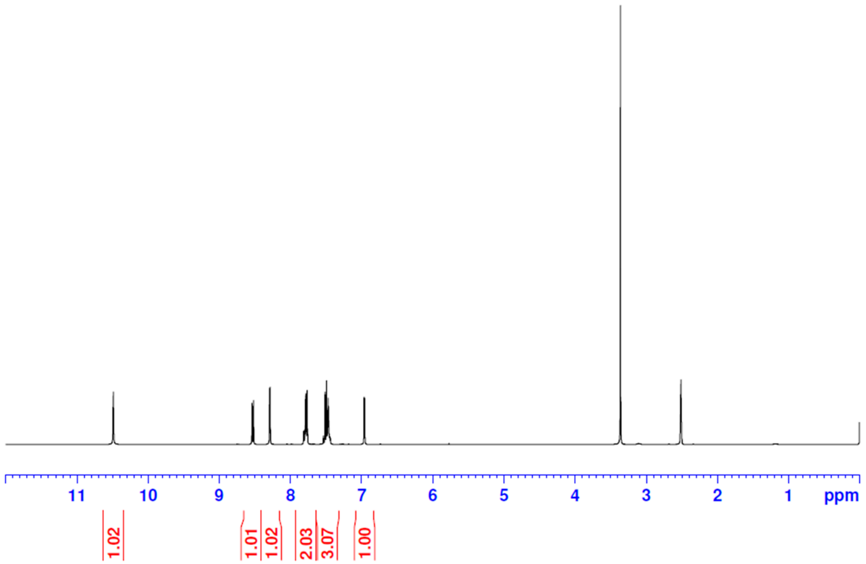

Calculated HRMS [M - H] - 296.0635

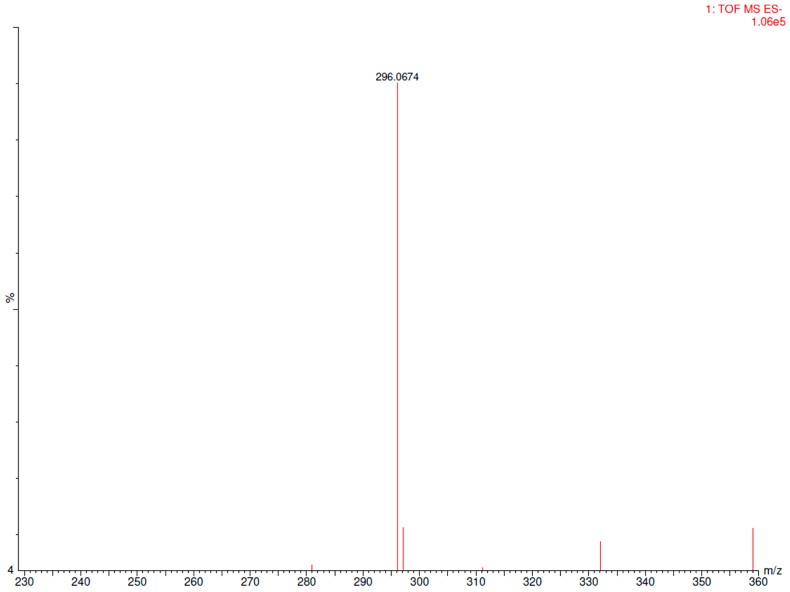

4-Cyano-*N*-(3,4-difluorophenyl)-1*H*-indole-1-carboxamide (4h)

^{19}F NMR (DMSO-d$_6$)

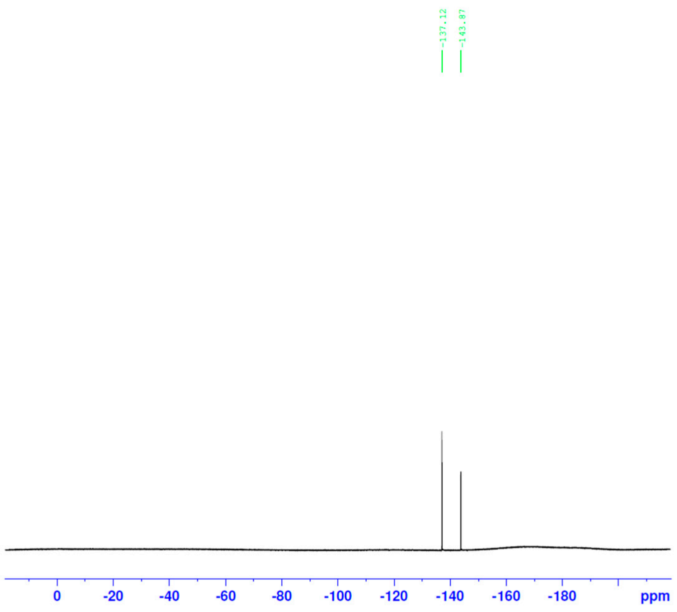

2D COSY NMR (DMSO-d$_6$)

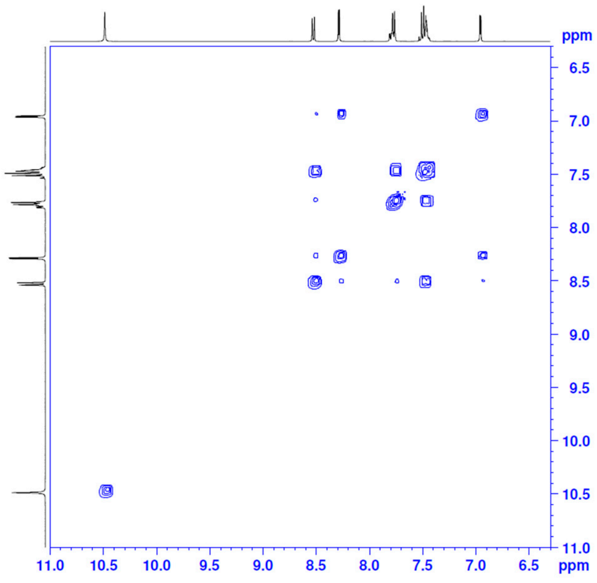

3,4-Difluorophenyl (3,4-difluorophenyl)carbamate (4i)

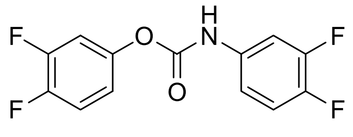

^{1}H NMR ($CDCl_3$)

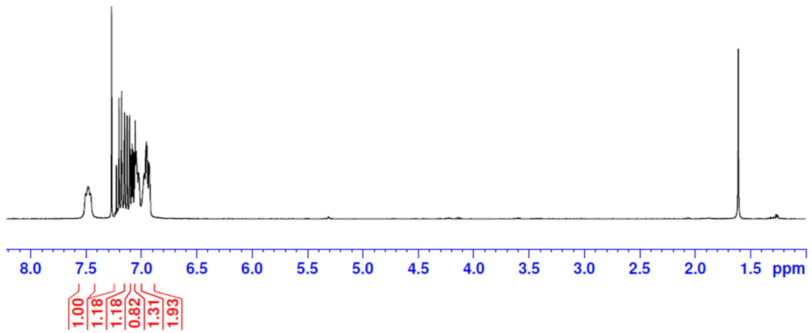

Calculated HRMS [M + H] + 286.0491

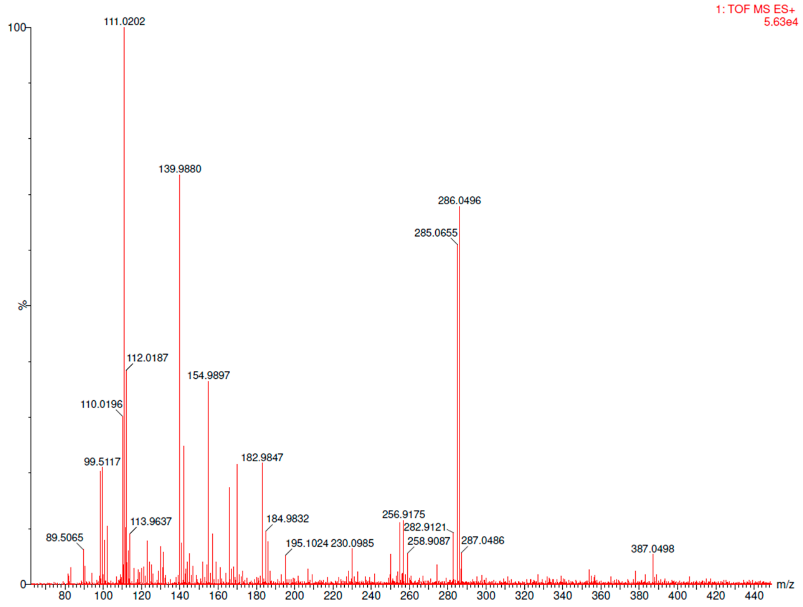

3,4-Difluorophenyl (3,4-difluorophenyl)carbamate (4i)

^{19}F NMR (DMSO-d$_6$)

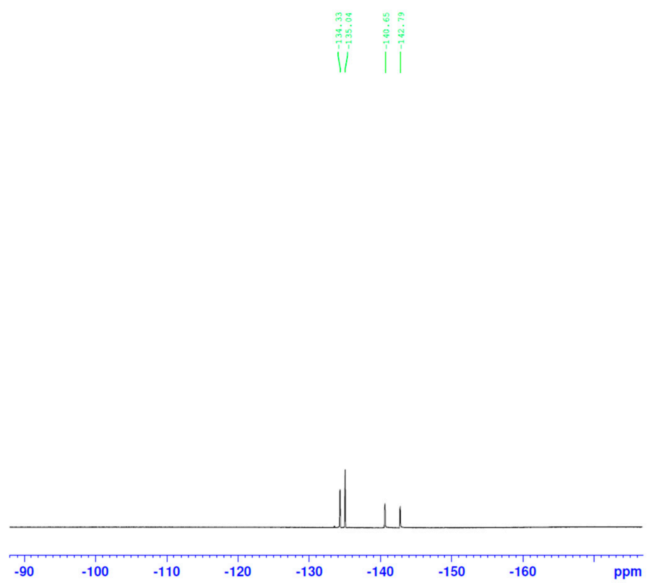

Purity (LC, t_{min} = 3.35): 98.74%

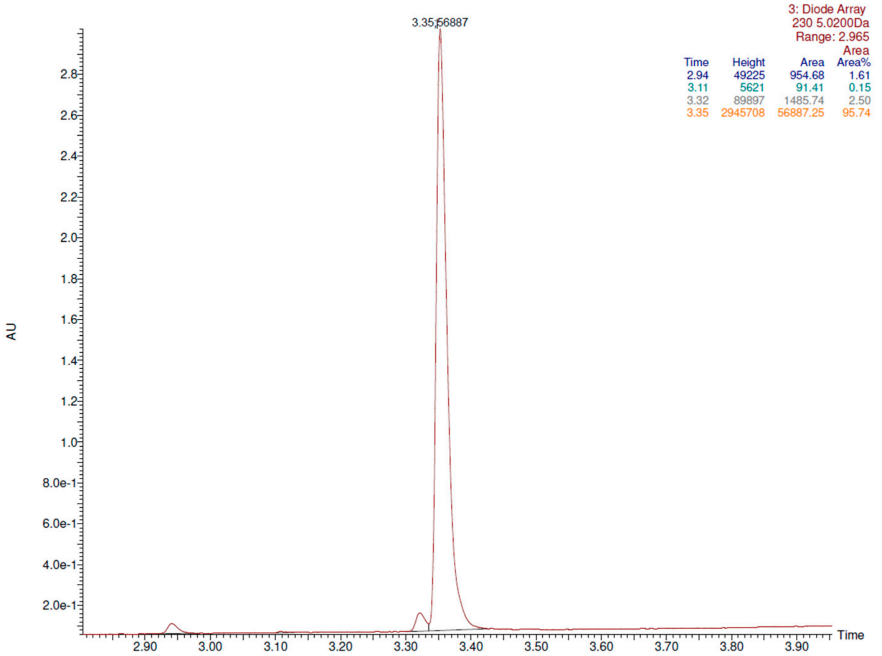

***N*,2-bis(3,4-difluorophenyl)acetamide (4j)**

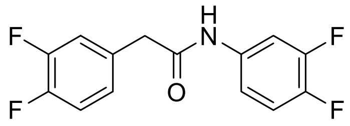

^{1}H NMR (DMSO-d$_6$)

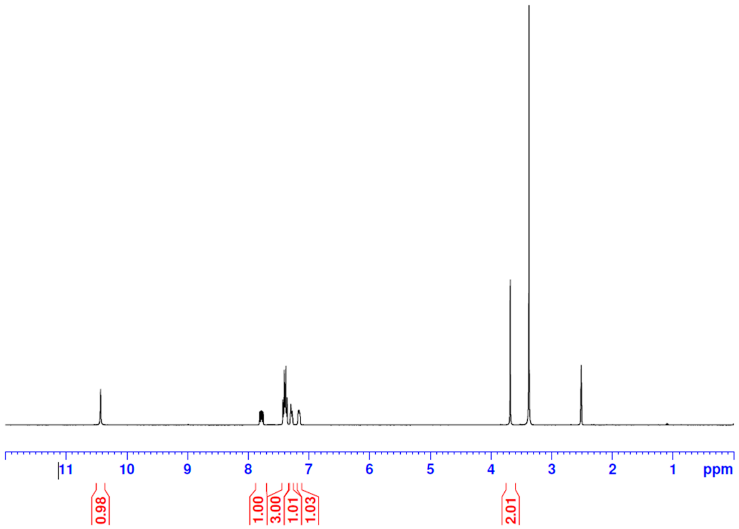

Calculated HRMS [M + H] + 284.0699

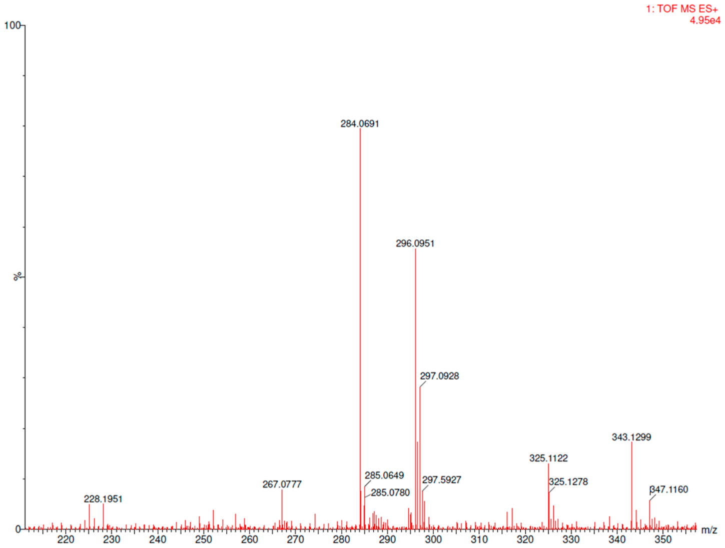

***N*,2-bis(3,4-difluorophenyl)acetamide (4j)**

2D COSY NMR ($CDCl_3$)

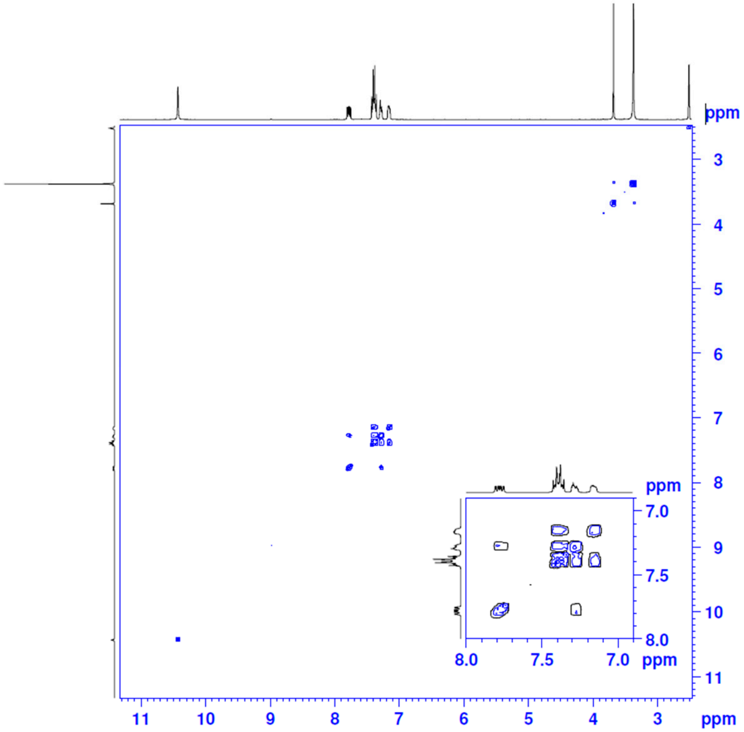

2D NOESY NMR ($CDCl_3$)

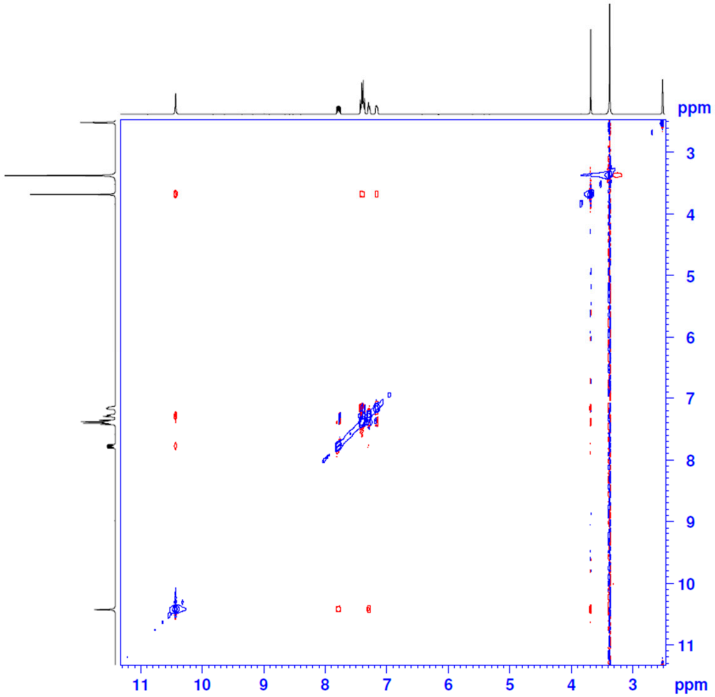

S-(3,4-Difluorophenyl) (3,4-difluorophenyl)carbamothioate (4k)

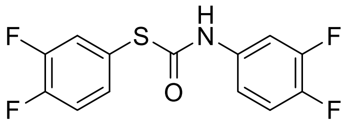

^{1}H NMR (CDCl$_3$)

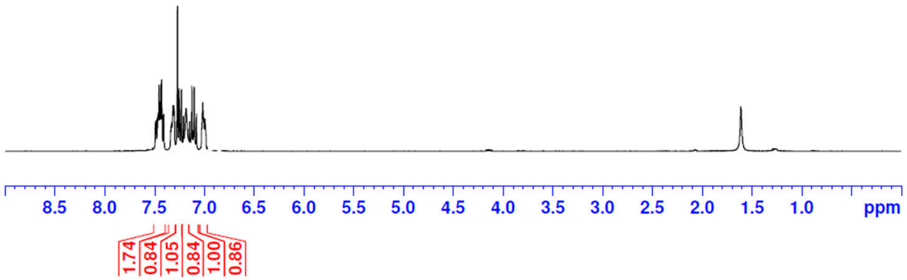

Calculated HRMS [M + H] + 302.0263

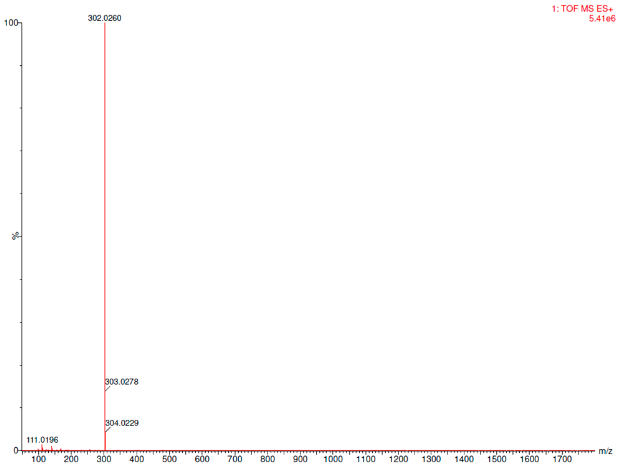

S-(3,4-Difluorophenyl) (3,4-difluorophenyl)carbamothioate (4k)

2D COSY NMR ($CDCl_3$)

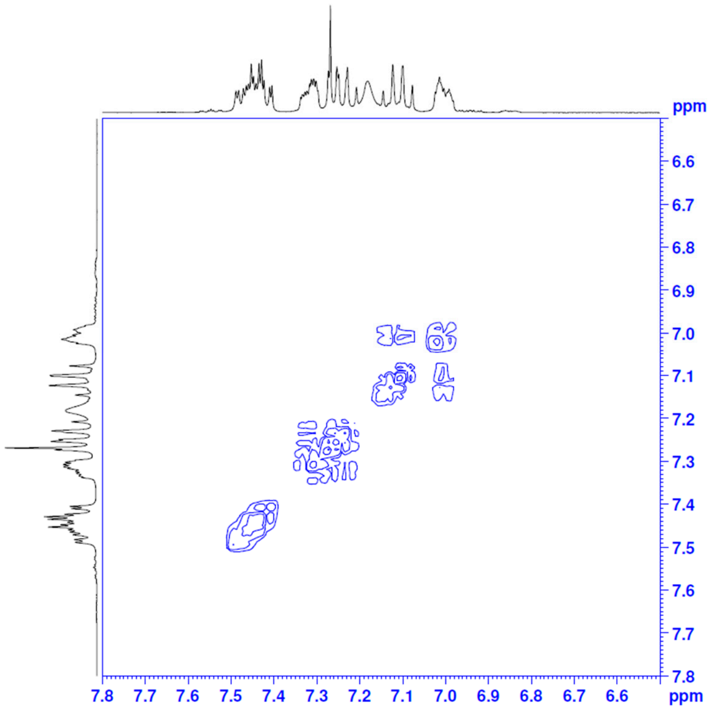

Purity (LC, t_{min} = 3.50): 97.90%

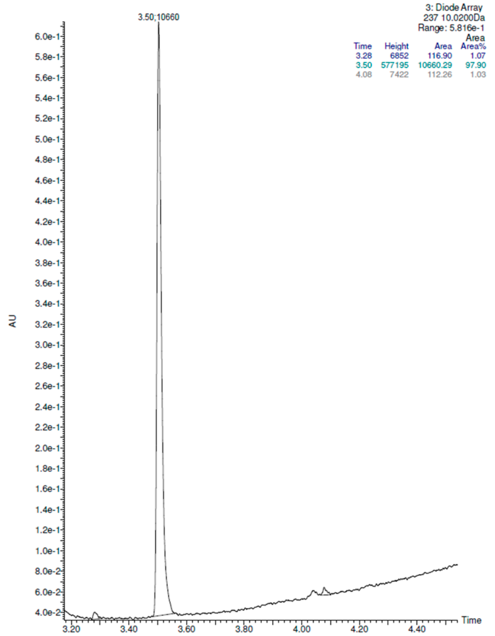

bis((1*R*,4*R*)-5-(4-Methoxyphenyl)-2,5-diazabicyclo[2.2.1]heptan-2-yl)methanone (5)

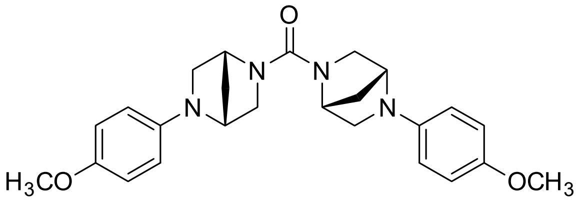

^{1}H NMR (DMSO-d$_6$)

Calculated HRMS [M + H] + 435.2396

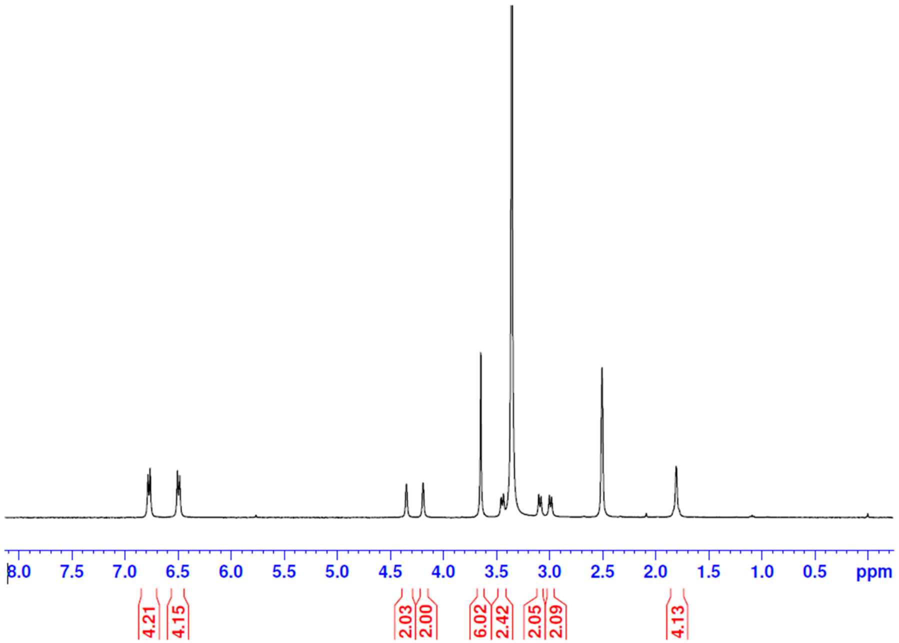

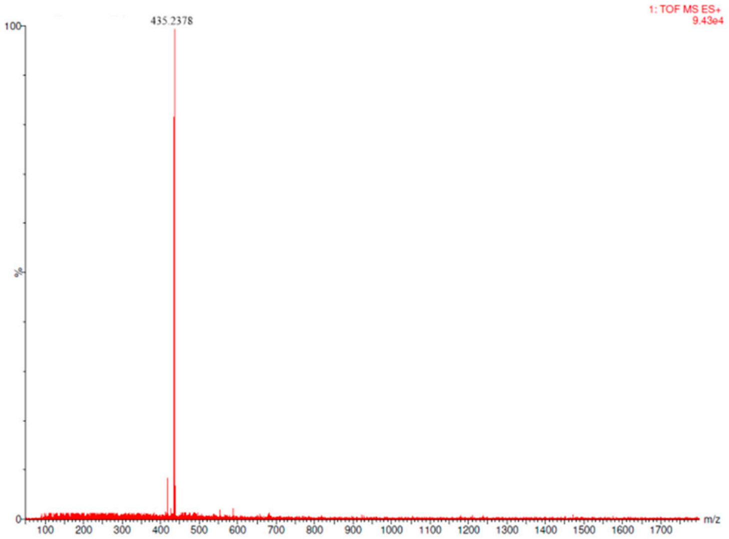

bis((1*R*,4*R*)-5-(4-Methoxyphenyl)-2,5-diazabicyclo[2.2.1]heptan-2-yl)methanone (5)

2D COSY NMR (DMSO-d_6)

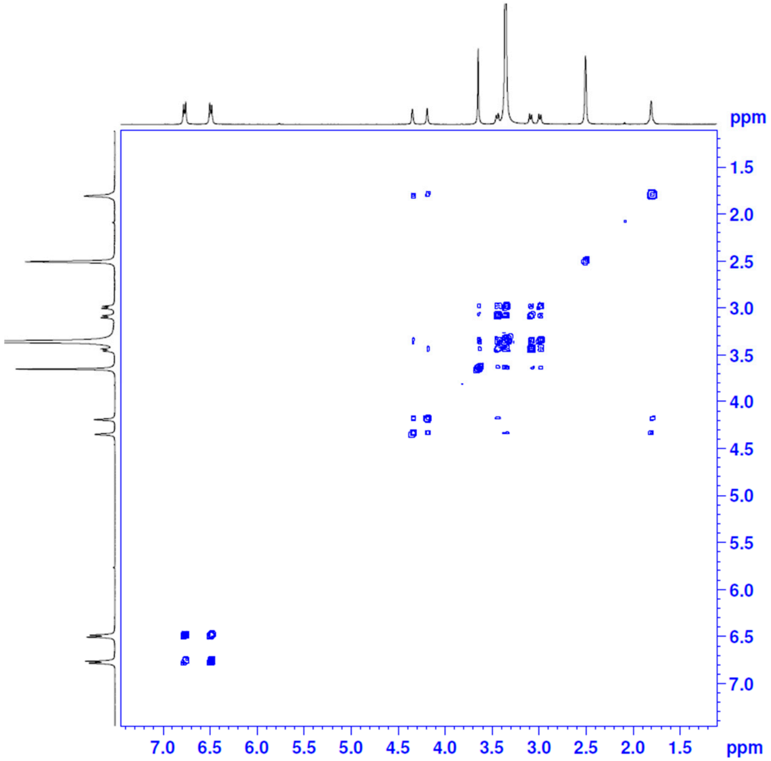

2D NOESY NMR (DMSO-d_6)

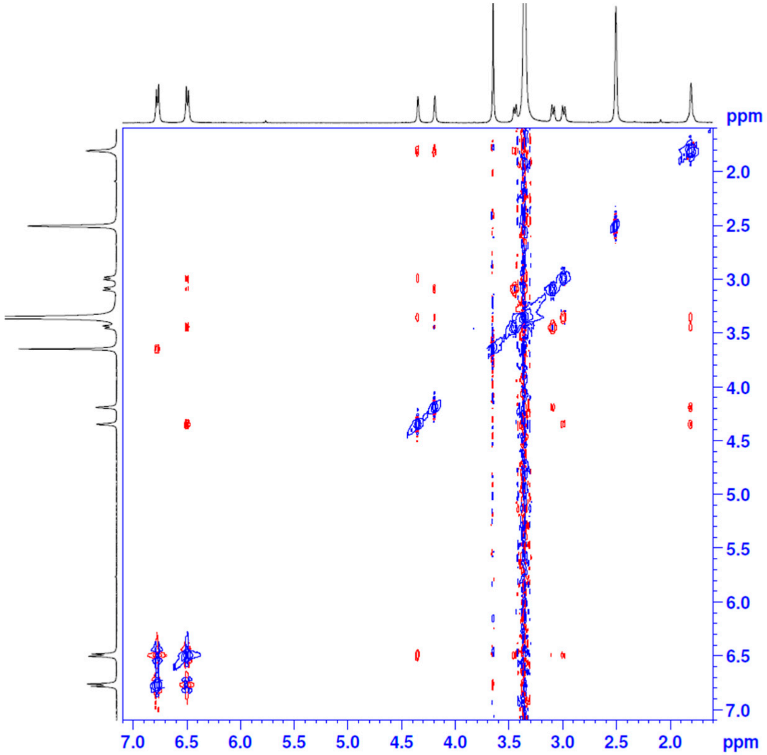

Supplement: Supplementary file 1 [file pharmaceuticals-18-00608-s001.zip › pharmaceuticals-3580731-supplementary.pdf]
